# Genophenotypic Factors and Pharmacogenomics in Adverse Drug Reactions

**DOI:** 10.3390/ijms222413302

**Published:** 2021-12-10

**Authors:** Ramón Cacabelos, Vinogran Naidoo, Lola Corzo, Natalia Cacabelos, Juan C. Carril

**Affiliations:** 1 Department of Genomic Medicine, International Center of Neuroscience and Genomic Medicine, EuroEspes Biomedical Research Center, Bergondo, 15165 Corunna, Spain; 2 Department of Neuroscience, International Center of Neuroscience and Genomic Medicine, EuroEspes Biomedical Research Center, Bergondo, 15165 Corunna, Spain; neurociencias@euroespes.com; 3 Department of Medical Biochemistry, International Center of Neuroscience and Genomic Medicine, EuroEspes Biomedical Research Center, Bergondo, 15165 Corunna, Spain; analisis@euroespes.com; 4 Department of Medical Documentation, International Center of Neuroscience and Genomic Medicine, EuroEspes Biomedical Research Center, Bergondo, 15165 Corunna, Spain; serviciodocumentacion@euroespes.com; 5 Departments of Genomics and Pharmacogenomics, International Center of Neuroscience and Genomic Medicine, EuroEspes Biomedical Research Center, Bergondo, 15165 Corunna, Spain; genomica@euroespes.com

**Keywords:** adverse drug reactions, cancer, cardiovascular disorders, central nervous system disorders, COVID-19, cutaneous ADRs, pharmacogenomics

## Abstract

Adverse drug reactions (ADRs) rank as one of the top 10 leading causes of death and illness in developed countries. ADRs show differential features depending upon genotype, age, sex, race, pathology, drug category, route of administration, and drug–drug interactions. Pharmacogenomics (PGx) provides the physician effective clues for optimizing drug efficacy and safety in major problems of health such as cardiovascular disease and associated disorders, cancer and brain disorders. Important aspects to be considered are also the impact of immunopharmacogenomics in cutaneous ADRs as well as the influence of genomic factors associated with COVID-19 and vaccination strategies. Major limitations for the routine use of PGx procedures for ADRs prevention are the lack of education and training in physicians and pharmacists, poor characterization of drug-related PGx, unspecific biomarkers of drug efficacy and toxicity, cost-effectiveness, administrative problems in health organizations, and insufficient regulation for the generalized use of PGx in the clinical setting. The implementation of PGx requires: (i) education of physicians and all other parties involved in the use and benefits of PGx; (ii) prospective studies to demonstrate the benefits of PGx genotyping; (iii) standardization of PGx procedures and development of clinical guidelines; (iv) NGS and microarrays to cover genes with high PGx potential; and (v) new regulations for PGx-related drug development and PGx drug labelling.

## 1. Introduction

Adverse drug reactions (ADRs) are becoming a major health concern worldwide in subjects undergoing either acute or chronic therapeutic interventions [1]. ADRs rank as one of the top 10 leading causes of death and illness in developed countries. Approximately half a million ADRs are reported in the USA each year, with direct medical costs of over $150 billion/year [2,3]. ADRs increase mortality, hospital admissions, hospitalization periods, health care costs, and withdrawal of drugs from the market [4]. 

Conventional type A (magnified) and type B (bizarre) ADRs show differential features depending upon genotype, age, sex, race, pathology, drug category, route of administration, and drug–drug interactions (DDIs) in polypharmacy [5,6,7]. ADRs of types A and B, and to a lesser extent C and D (Rawlins’ classification system), have been reported with many different categories of drugs post-marketing [8]. In many cases, ADRs are the consequence of DDIs involving pharmacokinetic or pharmacodynamic mechanisms [9]. However, substantial data give support to the idea that the individual genotypic profile is determinant for the manifestation of ADRs; consequently, pharmacogenomics (PGx) provides the physician effective clues for optimizing drug efficacy and safety [10,11,12,13].

ADRs are responsible for 6% of all hospital admissions and 9% of hospital expenditures [14]. The prevalence of ADR-related hospitalization in the general adult population is approximately 6–12% in different countries [15,16]. The most common drug category causing ADRs are cardiovascular drugs; and patients with ADRs need longer hospitalization periods for recovery. Patients in intensive care units are particularly vulnerable, with elevated risk of ADRs associated with specific medications [17].

Cardiovascular disorders (20–30%), cancer (25–30%) and central nervous system (CNS) disorders (10–20%) represent over 80% of morbidity and mortality in developed countries. The pharmacological treatment of these medical conditions represents over 20% of direct costs, and ADRs are present in over 40% of cases, contributing to approximately an additional 10% increase in health expenditures [11,12,13,14,15,16,17,18].

PGx accounts for 20–95% of drug response variability, with a significant role in the incidence and severity of ADRs [2,11,12]. Approximately 30% of ADRs at hospital admission are caused by drugs with PGx annotations [16]. Approximately 50% of current drugs already have an identified PGx profile which is useful for preemptive genotyping and clinical benefit for the patients in terms of improving efficacy and reducing ADRs [19,20,21,22,23].

The PGx machinery integrates the elements involved in a particular PGx-based pathway in which a series of reactions occur between drugs and gene products after drug administration. A PGx-pharmacokinetic pathway recapitulates the drug-gene events in the absorption, distribution, metabolism, and excretion (ADME) process; and a PGx-pharmacodynamic pathway represents the drug-gene events at the drug-target level when the drug displays its mechanism of action to neutralize pathogenic events associated with pathogenic genes [24]. The structural and functional integrity of a PGx-pathway results in drug efficacy, and dysfunctions and/or deficiencies in any PGx-based pathway are responsible for drug safety and toxicity, including ADRs [25]. The categories of genes involved in the PGx machinery include pathogenic, mechanistic, metabolic, transporter and pleiotropic genes under the regulatory control of the epigenome [11,12,26,27]. 

Approximately 80% of oxidative metabolism and 50% of elimination of drugs currently in use are attributed to metabolic enzymes of the CYP superfamily, mainly expressed in key splanchnic organs for drug oxidation and elimination, the liver and intestine. The phenotypic condition of normal metabolizer (NM), intermediate metabolizer (IM), poor metabolizer (PM) and ultra-rapid metabolizer (UM) associated with specific gene variants are determinant for drug efficacy and toxicity [19,28,29]. Quality control studies for 28 genes (*CYP1A1*, *CYP1A2*, *CYP2A6*, *CYP2B6*, *CYP2C8*, *CYP2C9*, *CYP2C19*, *CYP2D6*, *CYP2E1*, *CYP3A4*, *CYP3A5*, *CYP4F2*, *DPYD*, *GSTM1*, *GSTP1*, *GSTT1*, *NAT1*, *NAT2*, *SLC15A2*, *SLC22A2*, *SLCO1B1*, *SLCO2B1*, *TPMT*, *UGT1A1*, *UGT2B7*, *UGT2B15*, *UGT2B17*, and *VKORC1*), through consensus verification, confirmed the presence of at least 108 variant pharmacogenetic alleles [30].

The analysis of 1931 PGx biomarkers in 231 genes in 18 European populations showed significant inter-population PGx biomarker allele frequency differences, especially in seven clinically actionable PGx biomarkers in seven European populations, with effects in drug efficacy and safety associated with 51 medication treatment modalities. Differences in the prevalence of high-risk genotypes involved *CYP2C9*, *CYP2C19*, *CYP3A5*, *VKORC1*, *SLCO1B1* and *TPMT* variants [31]. Genome-wide association studies (GWAS) have identified novel associations for idiosyncratic ADRs, including liver toxicity, hypersensitivity, skin rash, and myotoxicity [32].

The implementation of PGx procedures for the personalized treatment of major problems of health with high cost for society would reduce total costs by 20–30% [33]. PGx-driven medication risk assessment in patients with polypharmacy is useful for identifying inadequate drug regimens and preventing ADRs [34]. Concerning the costs of drug development and clinical trials, when the response rate is raised by only 5%, the pharmaceutical industry would reduce the cost of a clinical trial by approximately 40% compared to that of a conventional trial [35].

In this article, we update recent findings on the PGx of ADRs, with special emphasis on major problems of health (heart, cancer, brain, COVID), and introduce for the first time an extensive catalog with the PGx of drugs for the treatment of cardiovascular diseases and related disorders.

## 2. ADR-Associated Factors

### 2.1. Age-Related ADRs

Over 50% of term and preterm neonates are at high risk for serious ADRs. The most commonly reported system organ classes are injury, poisoning and procedural complications (16%), general disorders and administration site conditions (12.5%) and blood and lymphatic system disorders (12%). Over 70% of neonates with ADRs fully recover and less than 4% die as a consequence of ADRs. The pharmaceutical categories mostly incriminated in neonatal ADRs are anti-infectives, CNS drugs and alimentary tract drugs. The most frequently serious ADRs are caused by zidovudine, ibuprofen and nevirapine [36].

In newborn infants and pediatric age, drug efficacy and safety are influenced by growth and development on drug ADME, and age-specific conditions. Drug distribution in the neonate is influenced by age-dependent factors (total body water content, water distribution and hydration fluctuations, drug transporters, blood-tissue protein binding, blood-tissue pH, blood perfusion). PGx-related metabolic enzymes exhibit age-related expression: (i) enzymes expressed during the fetal period, which are silenced or expressed at low levels within 1–2 years after birth; (ii) enzymes expressed at constant levels throughout fetal development, with postnatal increased expression; and (iii) enzymes with expression after the third trimester of life. ADRs in pediatric patients are 3-fold more frequent than in adults due to DDIs and inappropriate treatments [37]. 

ADRs in children are different from those of adults, and data from clinical studies in adults cannot be extrapolated to children [38]. Although pediatric PGx needs extensive studies with most drugs, PGx screening may help pediatricians to adjust drug dose in order to reduce ADRs in children at risk [39]. Financial barriers (test reimbursement, test cost, and money) are the main obstacles perceived by pediatric institutions attempting clinical implementation [40]. Some countries have created Pharmacogenomics Networks for Drug Safety to develop personalized and genetic-based predictions of pediatric drug response and likelihood of experiencing ADRs [41]. Genotype-guided prescribing in pediatrics prevents ADRs and improve therapeutic response. Actionable genotypes, where a prescribing change may be indicated, provide value to PGx implementation. In a population of 2.9 million pediatric patients, the antiemetic ondansetron (annual prevalence of exposure: 8107 per 100,000 children), the opioids tramadol (295 per 100,000), codeine (571 per 100,000) and oxycodone (2116 per 100,000), and the antidepressants citalopram, escitalopram, and amitriptyline (250 per 100,000 for each) were commonly prescribed, with actionable exposures the highest for oxycodone and ondansetron (>300 per 100,000 patients annually). Most of these drugs are substrates of the CYP2D6 and CYP2C19 enzymes, with easy and cheap screening possibilities [39]. 

Cutaneous ADRs are the most prevalent ADRs (2–3%) in hospitalized children. Approximately 65.6% of the cases are severe and 34.4% are non-severe. L-asparaginase (16%), amoxicillin (8.3%), cotrimoxazole (7.2%), carbamazepine (4.9%) and lamotrigine (3.7%) account for approximately 40% of all suspected medications [42].

Cisplatin is a widely used chemotherapeutic agent in solid tumors. Permanent hearing loss is a serious ADR of cisplatin treatment in children. Genetic variants in *TPMT* (rs12201199) and *ABCC3* (rs1051640) are associated with cisplatin-related hearing loss, with a sensitivity of 50.3% and a specificity of 92.7% [43].

Despite the urgent need for fast development of pediatric PGx, advances in the past two decades have been rather poor. Less than 10% of pediatricians are familiar with PGx and over 80% of pediatricians would request educational opportunities on PGx for data interpretation and therapeutic recommendations [44,45]. To minimize the risk of ADRs, integrating PGx into pediatric care should be a priority for most departments of pediatrics in hospitals and in ambulatory services of primary pediatric care [46,47].

ADRs are more prevalent in the elderly population [48]. Old people with chronic disorders may take over 10–12 different drugs/day and PGx may help optimize interventions aimed at improving the appropriate use of polypharmacy within this fragile population [49]. The risk of polypharmacy-related ADRs correlates with very old age, multiple co-morbidities, dementia, frailty, and limited life expectancy [50]. Multimorbidity and polypharmacy are important risk factors for drug-related hospital admissions in older people. Over 50% of hospitalized older adults under polypharmacy regimes have a higher frequency of defective pharmagenes [51]. The causes of prescribing errors in the elderly are multifaceted and complex, including prescribers’ lack of experience in the management of geriatric disorders, deficient knowledge in pharmacotherapy, overprescribing polypharmacy regimes, and inappropriate prescribing.

Several drugs (included in the Beers Criteria) have higher chances of causing ADRs in the geriatric population. The residents of nursing homes receive a higher number of potentially inappropriate medication (PIM) than their counterparts from tertiary care hospitals. PIMs prescribed to females in nursing homes are higher than in other settings. Approximately 55% of residents of nursing homes receive at least one PIM (27% two PIMs), compared to approximately 26% (2% two PIMs) in other medical institutions. Benzodiazepines are the most commonly prescribed PIMs in nursing homes, and antacids are the most common PIMs in tertiary care centers. The second most common PIM in 15% of nursing home residents are non-steroidal anti-inflammatory drugs [52]. Inappropriate drug exposure, pharmacokinetic and pharmacodynamic changes, reduced homeostatic reserve and DDIs contribute to ADRs in old age [53].

Although many recommendations and guidelines have been proposed from many good-will sources, PGx has not been introduced in geriatric medicine with enough determination to fight the dangerous pandemic of polypharmacy-related ADRs in the elderly [54,55,56,57,58].

### 2.2. Sex-Related ADRs

Pharmacokinetic parameters (oral bioavailability, absorption, intestinal and hepatic metabolism, and renal elimination) are different in women and men; and pharmacodynamic parameters also show gender differences [59]. Sex-related differences in structural genomics, gene expression, epigenetics, cellular regulatory pathways, and physiological functions (sex steroids) may directly and indirectly affect drug absorption, distribution, metabolism, and elimination, contributing to ADRs [60].

Sex-related PGx may account for differences in the onset of ADRs between females and males.

There are differences in CYP-related enzyme activity between females and males; for instance, CYP3A4 activity is higher in women than men, whereas CYP1A2 activity tends to be lower in women than in men. UGT-related glucuronidation is slower in women.

Women tend to manifest 30–50% more ADRs than men. Main reasons for this sex difference are attributed to polypharmacy, DDIs, sex differences in pharmacokinetics or pharmacodynamics, and PGx profile variation. In general, women tend to be overmedicated due to sex-related pharmacokinetic profiles. Most FDA-approved drugs show higher blood concentrations and longer elimination times in women, in parallel with ADRs. The evaluation of 86 drugs revealed that (i) 76 drugs had higher pharmacokinetic values in women; (ii) 59 drugs showed sex-biased ADRs in 88% of the cases; and (iii) 96% of drugs with female-biased pharmacokinetic values were associated with a higher incidence of ADRs in women than in men [59,61].

Sex-related ADRs have been found in patients treated with medications for heart failure (HF). ADRs are more frequent in females and in older patients with HF. A higher incidence of ADRs has been reported in women treated with angiotensin-converting enzyme (ACE) inhibitors, digoxin, or mineralocorticoid receptor antagonists. No sex-related ADRs have been found in patients treated with angiotensin II receptor blockers and β-blockers [62]. Following treatment with several drugs, there is a higher incidence of women with drug-induced QT prolongation and a potentially fatal arrhythmia such as torsades de pointes [63]. Women have a longer baseline QT interval, a higher mortality rate after myocardial infarction, and a higher rate of hemorrhagic stroke after receiving thrombolytic therapy than men. Pain relief with opioids is also more effective in women than men. Both sexes differ in their capacity for metabolizing drugs via phase I and phase II enzymes. Substrates of CYP2C9, CYP2C19, CYP2D6 and CYP3A4/5 enzymes and/or ABCB1 transporter elicit differential effects in females and males [19]. The levels of thiopurine methyltransferase (TPMT), the enzyme responsible for the metabolism of 6-mercaptopurine, are approximately 14% higher in the liver of males who require higher doses of the drug [19,64].

Males have worse overall survival and a higher risk of death for acute lymphoblastic leukemia, ependymoma, neuroblastoma, osteosarcoma, thyroid carcinoma, and malignant melanoma among children with 18 different types of cancer [65]. Sex effects are seen on interventions for depression, type 2 diabetes mellitus, and chronic pain conditions [66]. Sex also influences the incidence and progression of a wide variety of diseases and conditions related to transplantation probably due to effects in the pharmacokinetics and pharmacodynamics of immunosuppressive and anti-infective drugs in transplant recipients [67].

ADRs are also more frequent in women than in men treated with selective serotonin reuptake inhibitors (SSRIs) [68]. Psychotic symptoms in women respond to neuroleptics at lower doses than in men, indicating that, at conventional doses, women are overmedicated and, consequently, more exposed to overdose-related ADRs [69]. 

ADRs are responsible for 5% of unplanned hospital admissions, especially in women who are 1.5–1.7-fold more likely to develop ADRs. Anticoagulants show a lower risk of admission with persistent hematuria, hemoptysis and subdural hemorrhage in women than in men and a higher risk of rectal bleeding in women. Women treated with diuretic thiazides also have a higher risk of admission than men in conditions of hypokalemia and hyponatremia [70].

Sex-related ADRs are in part associated with variants in metabolic (CYPs) and transporter genes (ABCs) which determine the condition of NMs, IMs, PMs and UMs for substrates, inhibitors and inducers in females and males [11]. 

### 2.3. Ethnic Differences in ADRs

Ethnic differences should be taken into account in drug prescription. Extensive data document the impact of ethnic variation on drug efficacy and safety [19], especially in Asian, African, Jewish and Arab populations [71].

The function and expression of CYP enzymes are highly variable, influencing drug exposure and therapeutic outcomes. The expression of CYP enzymes is under the control of many transcription factors (TFs) in the liver, where master regulators for the expression of several CYP enzymes show racial differences [72,73]. 

The frequencies and multiethnic distribution of the three most common *CYP2C19* alleles (*2, *3, and *17) in 2.29 million Americans revealed overall frequencies of CYP2C19*2, *3, and *17 of 15.2%, 0.3%, and 20.4%, respectively, with high ethnic variation. The most common diplotypes are CYP2C19*1/*17 (26%) and CYP2C19*1/*2 (19.4%), and the less common diplotypes are CYP2C19*2/*17 (6.0%), CYP2C19*17/*17 (4.4%) and CYP2C19*2/*2 (2.5%). Approximately 15% of patients are prescribed one or more high-PGx-risk CYP2C19 medications (e.g., anti-ulcer, proton pump inhibitors; antidepressants, selective serotonin reuptake inhibitors; anticoagulants, clopidogrel; and antifungals, voriconazole) [74].

CYP3A4 is involved in the metabolism of hundreds of xenobiotic agents. *CYP3A4* mutants encoding enzymes with loss of activity in PM phenotypes can cause severe alterations in the metabolism of specific drugs, inducing severe ADRs [75]. CYP3A5 enzymes (CYP3A5*1, functional allele; CYP3A5*3 (rs776746), non-functional variant) metabolize a large number of endogenous substrates and xenobiotics. The frequency of CYP3A5*3 is the highest in Europeans. In indigenous populations from Mexico, higher frequencies of CYP3A5*1 and *3 are observed in groups with higher (>90%) and lower (<90%) Amerindian ancestry, respectively. The CYP3A5*3/*3 genotype is more frequent in women with hypertension. The CYP3A5*1 allele shows protection against hypertension [76]. In the Spanish population, the loss-of-function variant CYP3A4*20 (1.2%) is associated with the increased frequency of ADRs [77].

The first study of genetic anthropology in 56 Arab populations from the Middle East and North African regions distinguishes 4 major groups: (i) North Africans (Algerians, Tunisians, Moroccans, and Libyans), as well as the first Arabian Peninsula cluster (Saudis, Kuwaitis, Yemenis), all related to Western Mediterraneans and Iberians; (ii) Levantine Arabs (Palestinians, Jordanians, Lebanese, and Syrians), Iraqi and Egyptians, related to Eastern Mediterraneans; (iii) Sudanese and Comorians, in clusters with Sub-Saharans; and (iv) the second Arabian Peninsula cluster, integrated by Omanis, Emiratis, and Bahrainis. The Berbers and Kurdish minorities are indigenous and genetically similar to host and neighboring populations. Concerning HLA class I (-*A*, -*B*) and class II (-*DRB1*, -*DQB1*) genes, the most frequent HLA class I alleles in Arabs are A*01, A*02, B*35, B*51, DRB1*03:*01*, DRB1*07:*01*, DQB1*02:*01*, and DQB1*03:*01*, whereas DRB1*03:*01*-DQB1*02:*01* and DRB1*07:*01*-DQB1*02:*02* are the most prevalent class II haplotypes [78]. Egyptians and Jordanians share a similar distribution and frequency of *CYP1A1*, *CYP3A4*, *CYP3A5*, *CYP2C9*, *CYP2C19*, *CCYP2E1* and *DPYD* variants with other Caucasian populations [79]. *CYP2C9*, *CYP2C19*, *CYP2D6*, *CYP1A1*, *NAT2* and *ABCB1* variants in the Russian population are also similar to Caucasians [80]. The frequency of the *CYP2C9* and *CYP2C19* variants in Iranians is similar to that in other Caucasian populations, except the CYP2C9*3 allele which differs significantly [81]. In Albanians, Romani and Macedonians, *CYP2D6* variants do not differ from other European populations [82]. *CYP2C9* allele and genotype variation in the Sistani ethnic group from Gorgan, South East of Caspian Sea and North East of Iran, shows important differences when compared to other populations (CYP2C9*1/*1, 53.9%; CYP2C9*1/*2, 22.1%; CYP2C9*1/*3, 11.4%; CYP2C9*2/*2, 2.9%; CYP2C9*2/*3, 4.3%; CYP2C9*3/*3, 0%) [83].

*CYP2C9* and *VKORC1* variants involved in warfarin PGx show important differences in Greek-Cypriots and Hellenes of Greece as compared to Caucasians, Asians and Africans. Approximately 18% of the Greek-Cypriots are carriers of more than three risk alleles, and approximately 50% of the members of this population are carriers of at least two independent risk alleles associated with warfarin sensitivity, with potential high risk for bleeding at conventional doses of anticoagulants [84]. In the Croatian population, *CYP2C9*, *CYP2C19* and *CYP2D6* variants show frequencies similar to other European population with intermediate values between mid-Europeans and Mediterranean populations [85].

In the Italian population, *CYP2C9*, *CYP2C19* and *CYP2D6* variants also show similar frequencies to those of other Caucasian populations; however, in Spain and Italy, as well as in other Mediterranean populations, the frequency of *CYP2D6*-UMs is higher than in North Europeans, showing a decreasing gradient of *CYP2D6*-UM from African to North European populations [1,86]. The distribution and frequency of *CYP2C19* and *CYP2D6* alleles across Europe show clear South–North gradients. The frequencies of *CYP2D6* gene duplications decrease from South-East to North-West, with <1% in Sweden and Denmark to 6% in Greece and Turkey, and an inverse distribution of the loss-of-function alleles CYP2D6*4 and CYP2D6*5. The inactive CYP2C19*2 allele also shows a North-West to South-East gradient. The CYP2C19*17 allele is prevalent in Central Europe (25–33%) with decreasing prevalence in Mediterranean-South Europeans (11–24%) [87]. In the Ashkenazi Jewish (AJ) population of New York, *CYP2C9* geno-phenotypes do not differ from those of other North American Caucasian populations; however, the frequency of CYP2C19*4 is different; and the frequency of *CYP2D6*-UMs is 2-fold higher than that in other North American Caucasians, mimicking the numbers observed in Mediterranean populations [88]. The prevalence of *CYP2D6*-PMs in the Yoruba Nigerians from Africa is similar to that reported in blacks [89].

In the Vietnamese Kinh population, a major ethnic group in Vietnam, no subjects with the CYP2C9*2 allele were detected, similarly to other Asians populations. CYP2C9*3 is the major allelic variant in the Kinh population [90]. Novel single-nucleotide variants (SNVs) and structural variations (SVs) in the *CYP2D6* gene have been identified in the Kinh population. Some of the novel SNVs (3157G>T (R329L), 3851G>A (W358X), 2988G>A) affect enzyme activity. The most common alleles in this Vietnamese population are CYP2D6*10 (43.75%), *1 (18.75%), and the tandem arrangement *36-*10 (12.13%) [91]. The frequencies of *CYP2C9* variants in the Chinese Li minority population are similar to those reported in East Asians and Africans [92].

Well-established differences among ethnic groups in drug responses are primarily due to genetic diversity of pharmagene variants. The study of 85 Very Important Pharmacogenes (VIP), according to PharmGKB criteria, in the Lhoba population of Southwest China, showed that 23, 28, 16, 10, 20, 16, 24, 19, 22, 21 and 36 of the selected VIP variant genotype frequencies differed from those of the African Americans from the Southwestern United States (ASW), Utah residents with ancestry from Northern and Western Europe (CEU), Han Chinese in Beijing, China (CHB), Chinese in Metropolitan Denver, Colorado (CHD), Gujarati Indians in Houston, Texas (GIH), Japanese in Tokyo, Japan (JPT), Luhya in Webuye, Kenya (LWK), MEX, Maasai in Kinyawa, Kenya (MKK), Toscani in Italy (TSI), and Yoruba in Ibadan, Nigeria (YRI) of the 11 major HapMap populations [93].

A meta-analysis of 336,000 subjects from different ethnic groups showed that the probability of having a *CYP2D6* non-NM predicted phenotype was highest in Algeria (61%) and lowest in Gambia (2.7%), and the same probability for *CYP2C19* was highest in India (80%) and lowest in Mexico (32%). The probability of having a non-NM predicted phenotype worldwide is 36.4% for *CYP2D6* and 61.9% for *CYP2C19* [94].

Combined deletion/duplication allele frequencies in *CYP2A6*, *CYP2B6* and *CYP2E1* range from 2 to 10% in African American, Asian, Caucasian, Hispanic and Ashkenazi Jewish populations. Common *CYP450* CNV formation might be mediated by non-allelic homologous recombination resulting in both full gene and gene-fusion copy number imbalances that affect drug efficacy and toxicity [95].

In Native American and Ibero American populations, classified as Native Americans, Admixed Latin Americans, Afro-Latin Americans, white Latin Americans from Cuba, Iberians, and Argentinean Ashkenazi Jews, ang geographically differentiated as North-, Central-, and South Amerindians (from Mexico, Costa Rica, and Peru, respectively), the distribution and frequency of *CYP2D6*, *CYP2C9*, and *CYP2C19* variants show ethnic differences. Native Americans harbor more wild-type alleles and lower CYP2D6*41, CYP2C9*2, CYP2C19*17 variants, with a smaller number of *CYP2C19*-UMs [96].

The human leukocyte antigen (HLA) genotypes associated with cutaneous ADRs include *HLA*-B*57:*01* for abacavir in Caucasians, *HLA*-B*58:*01* for allopurinol in Asians, *HLA*-B*15:*02* in Han Chinese and *HLA*-A*31:*01* in Europeans and Koreans for carbamazepine, *HLA*-B*59:*01* for methazolamide in Koreans and Japanese, and *HLA*-B*13:*01* for dapsone in Asians [97].

According to these ethnic differences in drug efficacy and safety, predictive PGx genotyping is recommended for patients treated with common drugs; furthermore, caution should be taken when extrapolating data from clinical trials performed in Caucasians to other populations [94,98].

## 3. Cardiovascular Disorders

PGx holds promise for optimizing personalized treatments in cardiovascular disease management, in which multiple categories of drugs are currently used [99] (Table 1, Table 2 and Table 3). The cost-effectiveness of implementing PGx in cardiovascular disease care is unclear, depending upon drug category and risk of ADRs. Most studies (78%) examine warfarin-*CYP2C9*/*VKORC1* or clopidogrel-*CYP2C19*, with supportive evidence for cost-effectiveness. Over 65% of the studies found PGx testing to be cost-effective in heart disease care; however, cost-efficiency varies across drugs and conditions [100].

Over 50% of people older than 60 years of age are regular consumers of drugs for the treatment of cardiovascular and related disorders (i.e., myocardial infarct, atrial fibrillation, hypertension, hypercholesterolemia, dyslipidemia, atherothrombosis, and stroke). Table 1 shows the most recent information on the PGx and pharmacological properties of agents acting on the renin–angiotensin system (ACE inhibitors, angiotensin II antagonists) and other antihypertensive drugs, β-blocking agents, calcium-channel blockers, cardiotonic agents, and diuretics. Table 2 shows the PGx of lipid-modifying agents; and Table 3 shows the PGx of antithrombotic drugs, including vitamin K antagonists, heparins, platelet aggregation inhibitors, direct thrombin inhibitors, and direct factor Xa inhibitors. Most of these drugs are taken in a polypharmacy fashion (>6 drugs/day), with the consequent increase in DDIs and ADRs [101]. In patients with these health conditions, PGx pre-emptive testing is highly recommended to improve efficacy and minimize toxicity [102]; however, the PGx of cardiovascular and related disorders in practice is still in a very primitive stage, despite the abundance of information available for the personalized use of cardiovascular drugs [23,103] (Table 1, Table 2 and Table 3).

Over 20% of cardiac catheterization patients undergoing angiography might benefit from pre-emptive polygenic PGx testing. Common gene–drug pairs for these interventions include *CYP2C19* for clopidogrel and antidepressants, *CYP2D6* for antidepressants and codeine, *SLCO1B1* for simvastatin, and *VKORC1*/*CYP2C9* for warfarin [104].

Hypertension is a leading cause of cardiovascular morbidity and mortality. Heritability accounts for 30%–50% genetic variation in blood pressure levels, and blood pressure control is very uneven in patients treated with different antihypertensive agents (diuretics, angiotensin-converting enzyme inhibitors, angiotensin II receptor blockers, beta-blockers, and calcium-channel blockers) (Table 1). Genetic factors affect both the risk for hypertension and the therapeutic response to antihypertensive drugs. Sex- and age-related differences in the prevalence of hypertension might be partly induced by estrogen. European Americans with hypertension have a better response to β-blockers than African Americans. *FGD5* rs294610 associates with increased diastolic blood pressure response to β-blockers, and *SLC4A1* rs45545233 associates with a poor response to β-blockers [105].

Screening of catechol-O-methyltransferase gene (*COMT*) variants (rs4680, rs737865, rs165599), uridine-diphospho-glucuronosyltransferase 1A gene family (*UGT1A*) variants (rs2070959, rs887829), and the *CYP19A1* (aromatase) rs10046 variant showed that all *COMT* variants are associated with the effect of diuretics, calcium-channel blockers and angiotensin-receptor blockers on systolic and diastolic blood pressure. The *COMT*-*Val*/*Met* (rs4680) variant is associated with lower systolic blood pressure in patients treated with calcium-channel blockers [106]. Although the PGx of many antihypertensive drugs is relatively well defined (Table 1), only a small percentage of the genetic variability on response to antihypertensive drugs has been explained due to insufficient application of PGx procedures to treat hypertension [19,107,108].

PGx studies with aspirin identified 38 aspirin-response-related variants with clinical significance in 33 genes in the Chinese population. The frequency of seven variants (rs1050891, rs6065, rs7862221, rs1065776, rs3818822, rs3775291 and rs1126643) did not differ from European and East Asian populations; ten variants (rs2228079, rs1613662, rs4523, rs28360521, rs1131882, rs1047626, rs3856806, rs2768759, rs7572857 and rs1126510) showed frequencies similar to the East Asian population and different from the European population; the frequency of one variant (rs2075797) was identical to that found in Europeans and different with East Asians; and the frequencies of five variants (rs10279545, rs730012, rs16851030, rs1353411, rs1800469) differed from those of both East Asians and Europeans [109].

Congenital long QT syndrome (LQTS) is one of the most extensively investigated cardiac ion channelopathies in cardiology. However, the electrophysiological mechanisms for the onset of torsade de pointes induced by many different drugs remains obscure [110]. Drug-induced prolonged QT interval is a risk factor for ventricular arrhythmias and sudden cardiac death. PGx variation is one of the multiple risk factors for drug-induced QT prolongation. Pharmacokinetic and pharmacodynamic pathways are involved in QT prolongation. Ethnic-related variation in CYP enzymes and ABC transporters influence drug-induced QT prolongation, especially in Caucasians [111].

Anticancer drugs may induce proarrhythmic effects, including drug-induced QT interval prolongation, leading to a fatal polymorphic ventricular tachycardia. QT interval prolongation and torsade de pointes may be life-threatening effects of arsenic trioxides, anthracyclines, and other tumor-specific drugs [112].

Statins are the most widely used lipid-lowering drugs, with potential effects in the prevention of cardiovascular disease, by reducing plasma low-density lipoprotein cholesterol levels and modulating other pleiotropic effects. Statins are a source of ADRs associated with PGx factors (Table 2) [113]. Many studies have investigated the association between *SLCO1B1* -521T>C and -388A>G SNPs and the risk of statin-induced ADRs. One meta-analysis suggests that the *SLCO1B1* -521T>C polymorphism is a risk factor for statin-induced ADRs, especially for simvastatin users; however, no significant association for the -388A>G polymorphism is apparent [114]. Cholesterol-lowering response to statins show ethnic differences [115,116]. In white patients, *SLCO1B1* 521C is associated with smaller reductions in total cholesterol and low-density lipoprotein cholesterol in patients treated with simvastatin. No associations between *SLCO1B1* 521C and cholesterol response are detected in African Americans, and no associations between CYP3A4*22 or CYP3A5*3 and cholesterol response are observed in other populations [116]. Most studies convey that PGx testing prior to treatment with statins is effective in reducing ADRs in patients with mutant *SLCO1B1* [117]. Fibrates are a drug class with lipid-modifying properties used to lower blood triglyceride levels and raise high-density lipoprotein cholesterol levels. The response to fibrate treatment is also PGx related [118] (Table 2).

## 4. Cancer

It has been postulated that the tumor’s (somatic) genome dictates the effectiveness of anticancer therapy, and that the patient’s (germline) genome influences drug exposure and ADRs. Germline PGx associations (equivalent to pathogenic genes) are currently used to guide cancer treatment decisions [119]. The other components of the PGx machinery (mechanistic, metabolic, transporter, and pleiotropic genes) are common to any drug category acting in a promiscuous and redundant fashion [11,19,120].

A major goal of PGx in cancer chemotherapy is the avoidance of severe, potentially life-threatening drug toxicity. Polymorphic variants in drug-metabolizing enzymes, proteins transporters and epigenetic dysregulation are responsible for much of the interindividual differences in the toxicity of most chemotherapeutic agents [11,27,121].

Antineoplastic agents are the most common class of drugs causing ADRs [11,19]. The narrow therapeutic window of most anticancer therapies and the use of polypharmacy make DDIs a prevalent problem in the cancer population. Another important problem is cardiotoxicity associated with anticancer drugs in which PGx and epigenetic factors are involved [11,19,122].

The prevalence of pharmacogenetically actionable medications in advanced cancer patients is conservatively estimated to be approximately 7%, with 101 ADRs potentially preventable in 10,000 patients genotyped [123]. Molecular-targeted drugs may interfere with molecules that are overexpressed in target cancer cells. These drugs may cause life-threatening ADRs which deserve particular PGx attention [11,124].

In cancer treatment, although many drug-gene associations have been reported, the strength of evidence supporting each association can vary significantly. There are pharmacoeconomic studies for fluoropyrimidine, 6-mercaptopurine, irinotecan, carboplatin, cisplatin, erlotinib, gefitinib, cetuximab, panitumumab, and trastuzumab. When cost per life-year or cost per quality-adjusted life-year are reported, PGx testing is dominant in Asia (42%), Europe (21%), Canada (17%), and the US (5%), and PGx testing is cost saving in 17 of 19 cost-minimization comparisons and is favored when compared with genetically indiscriminate strategies [125].

**Tamoxifen**. Tamoxifen (TAM) is the standard of care for women with estrogen receptor positive breast cancer. Wide variability in the response of breast cancer to TAM is mainly due to interindividual genetic differences. TAM is metabolized to its active metabolites N-desmethyl TAM and 4-hydroxytamoxifen by CYP2D6, CYP3A4, CYP2C9, CYP2C19 and CYP2B6 enzymes; and N-desmethyl TAM is further metabolized to endoxifen by CYP2D6. Endoxifen is 30–100-fold more potent than TAM in suppressing estrogen-dependent cell proliferation. Both ADRs and lack of efficacy are highly influenced by *CYP2D6* variants [126]. Inter-ethnic ADRs are found in Asian women with breast cancer treated with tamoxifen. CYP2D6*4, 2D6*10, 2D6*41, 2D6*10/*10, CYP2C9*2, *ABCB1* C3435T and SLCO1B1*5 are the most common genotypes correlating with ADRs [127].

**Anthracyclines**. Anthracycline-induced cardiotoxicity occurs in up to 65% of treated patients. PGx intervention may reduce the incidence of congestive heart failure in pediatric cancer patients taking anthracyclines [128].

**Irinotecan**. Irinotecan (CPT-11) has been approved for the treatment of advanced or metastatic colorectal cancer. Irinotecan causes severe toxicity (e.g., neutropenia and diarrhea) which contributes to non-compliance and treatment cessation. Irinotecan bioactivation into its metabolite SN-38 and further detoxification are mediated by uridine diphosphate-glucuronosyl transferases (UGT1A1), and several ABC transporters (ABCB1, ABCC1-ABCC6, and ABCG2) are responsible for drug efflux into bile and urine, whereas SLCO1B1 enables its influx from blood into hepatocytes. SNPs in these enzymes and transporters result in increased systemic SN-38 level and toxicity [129]. Thymidylate synthase (*TYMS*) and *UGT1A* germline polymorphisms influence the therapeutic outcome of colorectal cancer (CRC) patients treated with irinotecan plus 5-fluorouracil (irinotecan/5FU). Genotyping of *TYMS* (5′TRP and 3′UTR), UGT1A1*28, UGT1A9*22 and UGT1A7*3 in metastatic CRC patients treated with irinotecan/5FU shows that the *TYMS* 3TRP/3TRP genotype is the only independent predictor of tumor response. UGT1A1*28/*28 predicts hematologic toxicity and UGT1A9*1/*1 associates with non-hematologic toxicity [130].

In the Thai population, treated with irinotecan for colorectal cancer, genotyped for *ABCB1* c.1236C > T, *ABCB1* c.3435C > T, *ABCC2* c.3972C > T, *ABCG2* c.421C > A, CYP3A4*1B, CYP3A4*18, CYP3A5*3, DPYD*5, UGT1A1*28, and UGT1A1*6 variants, *ABCC2* c.3972C > T was associated with grade 1–4 neutropenia; and patients carrying both UGT1A1*28 and *6 were associated with severe neutropenia [131]. Similar results were found in the Chinese population [132].

**Platinum**. Gastrointestinal (GI) toxicity is a common side effect following platinum-based chemotherapy, with variable incidence and severity in similar chemotherapeutic conditions. The interindividual differences in platinum-related GI toxicity are associated with genetic factors involved in platinum transport, metabolism, detoxification, DNA repair, cell cycle control, and apoptotic pathways. Sample size, ethnicity, design, treatment schedule, dosing, endpoint definition, and assessment of GI toxicity add complications to the interpretation of PGx-related ADRs [133].

Platinum-based chemotherapy is the first-line treatment for non-small-cell lung cancer (NSCLC). Platinum resistance and ADRs are major problems in platinum therapy. Variation in the *ATP7A* and *ATP7B* genes plays an essential role in the transport of platinum. 

The assessment of *ATP7A* (rs2227291 and rs6622665) and *ATP7B* (rs1061472 and rs9535826) polymorphisms revealed that *ATP7B* rs9535826 is associated with GI toxicity, and that carriers of the GG genotype show lower GI toxicity in patients with NSCLC [134].

The *ABCC4* gene encodes the multidrug resistance-associated protein-4 (MRP4), an ATP-binding cassette (ABC) efflux transporter which is highly expressed in bone marrow cells (BMCs). MRP4 protects cells against xenobiotic agents and chemotherapeutic drugs. MRP4 is responsible for the extrusion of oxaliplatin, a platinum-based chemotherapeutic drug, from BMCs and the diurnal expression of MRP4 in BMCs is associated with the dosing time-dependent changes in oxaliplatin-induced myelotoxicity [135].

**Vincristine**. Vincristine is an effective chemotherapeutic agent for several types of cancer, including acute lymphoblastic leukemia (ALL); however, vincristine-induced peripheral neuropathies restrict its clinical utility. *CEP72* rs924607 is associated with neuropathies, and ADME analyses show associations between neuropathies and *ABCC1* rs3784867, and *SLC5A7* rs1013940. *ABCC1* variation is involved in vincristine transport; and *SLC5A7* and *TTPA* rs10504361 may affect toxic neuropathies [136].

**6-Mercaptopurine**. 6-Mercaptopurine (6-MP) improves survival in children with acute lymphoblastic leukemia (ALL). A major dose-limiting toxicity of 6-MP is life-threatening myelosuppression associated with SNPs in genes coding for enzymes involved in 6-MP metabolism, such as *NUDT15*, *TPMT* and *ITPA*. The minor allele frequencies (MAFs) of *NUDT15* rs116855232, *TPMT* rs1142345 and *ITPA* rs11273540 in Chinese children with ALL are 15.7, 2.9, and 18.1%, respectively. *NUDT15* and *TPMT* variants associate with 6-MP dose intensity. *NUDT15-T/T* carriers are highly sensitive to 6-MP, with a dose intensity of 60.27% (T/C, 83.83%; C/C, 94.24%). The *NUDT15* variant is a predictor of early-onset leukopenia [137]. The genotyping of ITPA 94C > A (rs1127354) and *IVS2*+21A > C (rs7270101), TPMT*2 238G > C (rs1800462), TPMT*3B 460G > A (rs1800460) and *3C 719A > G (rs1142345), and *NUDT15* 415C > T (rs116855232) in Middle Eastern children with ALL treated with 6-MP showed that (i) *TPMT* and *NUDT15* variants are uncommon in the Middle East; (ii) ITPA variants are more common in Lebanese than in Kurdish children; and (iii) the most significant ADRs were found in carriers of *NUDT15*, TPMT*3A, and *ITPA* risk variants [138].

**Methotrexate**. Methotrexate (MTX) is used in the treatment of pediatric ALL. Since MTX targets the folate metabolic pathway, enzyme dysfunctions in this pathway associated with specific gene mutations may lead to ADRs. The analysis of variants in the thymidylate synthetase (TYMS), methylenetetrahydrofolate reductase (MTHFR), dihydrofolate reductase (DHFR), *SLC19A1* and *SLCO1B* genes showed that the A allele of *SLC19A1* c.80 variant contributes to slow MTX elimination and that the AA genotype is a predictor of MTX-related hepatotoxicity. Carriers of the homozygous *TYMS* 6bp deletion experience gastrointestinal toxicity [139].

**Aromatase inhibitors**. In breast cancer patients treated with aromatase inhibitors, the screening of *CYP19A1*, *CYP17A1*, *CYP27B1*, *TCLA1*, *RANK*/*RANKL*/*OPG*, and *ESR1*/*ESR2* variants may be of some utility to optimize positive effects and reduce ADRs [140]. *CYP19A1* variants are associated with clinical response and ADRs. Time to progression (TTP) in metastatic patients is increased in carriers of the rs4646 variant. Musculoskeletal ADRs associate with rs934635, rs60271534, rs700518rs, and haplotype M_3_5, and vasomotor symptoms are more prevalent in rs934635, rs1694189, rs7176005, and haplotype M_5_3 carriers. No ADRs have been associated with other SNPs (rs4646, rs10046, rs727479, rs1062033) [141]. The CYP3A4*1B, CYP3A5*3 and UGT1A4*2 variants may influence the onset of ADRs in women with estrogen receptor (ER)-positive breast cancer treated with anastrozole [142].

**Bevacizumab and Paclitaxel**. In patients with metastatic breast cancer receiving bevacizumab and paclitaxel as first-line chemotherapy, the triple-negative cancer phenotype, combined with specific SNPs in *VEGFA* (rs833061), *VEGFR1* (rs9582036) and *VEGFR2* (rs1870377), show predictive value for survival [143].

Paclitaxel resistance in ovarian cancer is a common clinical problem. Multiple epigenetic mechanisms are involved in drug resistance [27]. *RAB17* exerts regulatory effects on the non-coding RNA network of the paclitaxel-resistant ovarian cancer cell A2780/PTX. *RAB17* is highly expressed in A2780/PTX cells; and *RAB17* knockdown increases cell sensitivity to paclitaxel, inhibits proliferation, and causes cell cycle arrest in the G1 phase. *RAB17* is a new target of miR-370-3p, and Hsa_circ_0000714 acts as a sponge for miR-370-3p, regulating *RAB17* expression through the CDK6/RB signaling pathway [144].

**Thiopurines**. Thiopurine S-methyltransferase (TPMT, EC 2.1.1.67) deficiency increases the risk of ADRs in patients receiving thiopurines. Sensitivity and specificity of phenotyping for deficient patients are 75.9% and 98.9%, respectively, and for TPMT*2 and TPMT*3 genotypes 90.4% and 100.0%, respectively. In cases with deficient or intermediate activity, phenotype sensitivity and specificity are 91.3% and 92.6%, respectively, and for TPMT*2 and TPMT*3 88.9% and 99.2%, respectively [145]. TPMT testing shows that approximately 90% of patients receiving thiopurines are wild-type and that nearly 30% of patients may benefit with TPMT testing and consequent dosing modification to reduce the incidence of myelosuppression [146]. Approximately 0.1% of patients are carriers of novel *TPMT* alleles (TPMT*42, *43, and *44) recently identified in Sweden [147].

Loss-of-function alleles in the *NUDT15* gene are common in Asians and Hispanics who show reduced degradation of active thiopurine nucleotide metabolites and higher predisposition to myelosuppression. Based on *TPMT* and *NUDT15* genotypes, starting doses of azathioprine, mercaptopurine, and thioguanine should be adjusted [148].

The first study in Korea on genetic polymorphisms affecting thiopurine metabolism pathways and toxicity in children with acute lymphoblastic leukemia showed that *ABCC4*, *AHCY*, *ATIC*, *FAM8A6P*, *GART*, *GNG2*, *GSTA1*, *MTHFD1*, *MTHFR*, *NUDT15*, *PACSIN2*, *TPMT*, *TYMS* and *XDH* variants, as well as an intronic polymorphism between *HIVEP2* and *AIG1*, are associated with thiopurine metabolism, and that *ABCC4*, *ADK*, *ATIC*, *GART*, *GMPS*, *GSTP1*, *IMPDH1*, *ITPA*, *KCNMA1*, *MOCOS*, *MTRR*, *NUDT15*, *SLC19A1*, *SLC28A3*, *SLC29A1*, *SLCO1B1*, *TYMP* and *XDH* variants are associated with thiopurine-related ADRs (neutropenia, hepatotoxicity, and treatment interruption) [149].

**Tyrosine kinase inhibitors**. Most tyrosine kinase inhibitors (imatinib, gefitinib, erlotinib, dasatinib, sunitinib, adavosertib, and lapatinib) induce PGx-related severe ADRs [19]. Two new standard-of-care first-line treatments (lenvatinib and atezolizumab + bevacizumab) and several new agents in second line (regorafenib, ramucirumab, and cabozantinib) are contributing to improving the treatment of advanced hepatocellular carcinoma (HCC). However, these treatments are not devoid of ADRs [150].

Sunitinib is a tyrosine kinase inhibitor for the treatment of metastatic renal cell carcinoma. Sunitinib ADRs (thrombocytopenia, leukopenia, mucosal inflammation, hand-foot syndrome, and any toxicity according to National Cancer Institute Common Toxicity Criteria higher than grade 2) are highly influenced by PGx factors. Leukopenia is increased in carriers of the G allele in *CYP1A1* 2455A/G and the T allele in *FLT3* 738T/C, or when CAG is absent in the *NR1I3* (5719C/T, 7738A/C, 7837T/G) haplotype. Sunitinib toxicity is increased in carriers of the T allele in the 1191C/T genotype of vascular endothelial growth factor receptor 2 or in carriers of a copy of TT in the *ABCG2* (-15622C/T, 1143C/T) haplotype. Mucosal inflammation is increased in carriers of the G allele in *CYP1A1* 2455A/G. Hand-foot syndrome is prevalent in carriers of a copy of TTT in the *ABCB1* (3435C/T, 1236C/T, 2677G/T) haplotype [151]. Asians show lower clearance and frequent ADRs with sunitinib treatment. At least seven SNPs in *FLT3*, *ABCB1*, *VEGFR2*, *ABCG2* and *BIM* are associated with ADRs, therapeutic response, and survival in Asians. There is a strong association of the *FLT3* 738T genotype with leucopenia; *FLT3* 738T, *ABCB1* 1236T, *ABCB1* 3435T, *ABCB1* 2677T, *ABCG2* 421A alleles and the *ABCB1*-3435-1236-2677 TTT haplotype with neutropenia; and primary resistance and inferior survival with the *ABCB1*-3435-1236-2677 TTT haplotype [152]. Recent studies to re-evaluate the associations of *ABCG2* and *ABCB1* polymorphisms with sunitinib-induced toxicity and efficacy in renal cell carcinoma indicate that the *ABCG2* rs2231142 A allele is associated with increased risk of thrombocytopenia and hand-foot syndrome (HFS) in Asians, with no apparent effect on sunitinib-induced hypertension or neutropenia. The *ABCB1* rs1128503 T allele is associated with a decreased risk of sunitinib-induced hypertension and worse progression-free survival (PFS). The *ABCB1* rs2032582 T allele is associated with worse PFS, with no effect on hypertension, HFS, or survival in Asians [153]. Sunitinib is hepatotoxic due to the presence of reactive metabolites and impaired clearance of sunitinib in the liver, together with disruption of several metabolic pathways (mitochondrial fatty acid β-oxidation, bile acids, lipids, amino acids, nucleotides, and tricarboxylic acid cycle intermediates) [154]. CYP1A2 and CYP3A4 enzymes contribute to oxidative defluorination of sunitinib to generate a reactive, hepatotoxic quinoneimine. The quinoneimine–GSH conjugate (M5) of sunitinib is formed in an NADPH-dependent manner. CYP1A2 enzymes generate the highest levels of defluorinated sunitinib (M3) and M5, with small participation of CYP3A4 and CYP2D6; and CYP3A4 is the major enzyme forming the primary active metabolite N-desethylsunitinib (M1). The CYP3A4 inhibitor ketoconazole reduces M1 formation by 88%, and the CYP1A2 inhibitor furafylline decreases M5 generation by 62%. CYP2D6 and CYP3A4/5 inhibitors also decrease M5 by approximately 50%. CYP1A2 enzymes are more efficient than CYP3A4 and CYP2D6 enzymes in the generation of M3 and M5 metabolites [155].

Imatinib is the first-line treatment of gastrointestinal stromal tumor (GIST). Interindividual variation in the pharmacokinetics and pharmacodynamics of imatinib has been associated with metabolic enzymes and transporters. SNPs in the *NR2A1* gene that encodes hepatocyte nuclear factor 4 alpha (HNF4α), a transcriptional regulator of genes involved in drug disposition, affect dose-adjusted imatinib-free plasma levels and ADRs. Free plasma levels show a wide interpatient variation, with sex-related differences; and dose-adjusted imatinib-free plasma levels correlate with body surface area. Of the five *NR2A1* variants (rs3818247, rs1884613, rs2071197, rs2425640, and rs736824), only rs736824 showed association with dose-adjusted imatinib-free plasma levels in males, and rs3818247 with ADRs [156].

**Fluoropyrimidins**. Fluoropyrimidine drugs are used for the treatment of solid cancers. Fluoropyrimidine toxicity in the first four cycles of 5-fluorouracil or capecitabine-based chemotherapy is associated with four *DPYD* variants (c.1905+1G>A, c.2846A>T, c.1601G>A and c.1679T>G) (6%), the *TYMS* 3′-untranslated region del/del genotype, the *MTHFR* c.1298CC homozygous variant, and *CDA* c.-92A>G and *CDA* c.-451C>T variants [157].

Capecitabine is used for breast cancer treatment. Frequent ADRs include diarrhea (52%) and hand-foot syndrome (52%). PGx predictors of capecitabine ADRs are SNPs in DPYD*5, *MTHFR* and near *TNFSF4* (*OX40L*), a gene implicated in autoimmunity. Genotype-gene expression analyses of skin identified rs11158568 with expression of *CHURC1*, a transcriptional activator controlling fibroblast growth factor, associated with hand-foot syndrome [158].

## 5. Central Nervous System Disorders

ADRs reported by consumers for CNS drugs from 2007 to 2011 in the European ADR database, (EudraVigilance) show the presence of 5% of ADRs in children, 58% in women and 42% in men. The most relevant ADRs were reported in consumers of antiepileptics (36%), parasympathomimetics (22%), and antidepressants (9%). Serious ADRs occur in 60% of antiepileptic users (especially pregabalin and varenicline), and in 15% of patients treated with antidepressants [159].

Since few CNS drugs have been approved for the past decade, classical PGx studies with psychotropic drugs remain valuable for drug use optimization [11,12,19,27,160]. Pretreatment PGx testing is highly recommended in major neuropsychiatric disorders such as schizophrenia, depression, bipolar disorders, panic disorder, autism spectrum disorder, epilepsy, and neurodegenerative disorders (Alzheimer’s disease, Parkinson’s disease), which require long-term, toxic treatments [11,12,19,160,161,162,163,164,165,166,167]. CNS drugs can act as substrates, inhibitors or inducers of enzymes encoded by metabolic genes. Among the 307 most frequently-used CNS drugs, antiepileptics represent 14.66%, antiparkinsonians 10.42%, antipsychotics 21.82%, anxiolytics 11.40%, hypnotics and sedatives 21.17%, and antidepressants 20.53%. Approximately 90% of these drugs use CYP enzymes as major metabolic pathways. CNS drugs are substrates, inhibitors or inducers of 58, 37 and 42 enzyme/protein gene products, and are transported by 40 different protein transporters [12].

Most clinically relevant genetic variation is associated with phase I drug metabolism [168]. CNS drugs are major substrates of CYP3A4 (71%), CYP3A5 (37%), CYP2D6 (60%), CYP2C19 (45%), and CYP1A2 enzymes (44%); inhibitors of CYP3A4 (22%), CYP2D6 (23%), CYP2C19 (20%), CYP1A2 (17%) and CYP2C9 (15%); and inducers of CYP2C9 (9%), CYP2D6 (7%), CYP3A4 (5%), CYP1A2 (4.5%), CYP2A6 (4.5%) and CYP2B6 (3.7%). Major transporters of CNS drugs are ABCB1 (29%), SLCA1 (20%), SLC6A4 (20%), CLCNs (15%), SLC6A3 (12%) and SLC6A2 (11%). Over 80% of patients are deficient metabolizers for the *CYP2D6*/*2C19*/*2C9*/*3A4* tetragenic cluster [12].

Late-life depression (LLD) affects up to 15% of older adults, with clinical features and ADR risk distinct from depression in younger adults. Predictive genetic testing in LLD is a challenge to improve treatment in this vulnerable population, and the PGx of antidepressants is common to any form of depression [11,12,19,163,169,170,171].

Antidepressants reduce the symptom burden in Major Depressive Disorder (MDD) in over 50% of the cases, but ADRs and treatment resistance still present major challenges in over 30% of the cases [19,172]. Some studies did not identify significant predictive effect of PGx in depressive patients [173,174], whereas others found that over 40% of depressive patients receive inappropriate medication, and that with the aid of PGx testing it is possible to reverse inefficacy and unwanted effects after treatment rectification [19,169,175,176]. Polygenic Risk Scores (PRS) may allow MDD patients to be stratified for antidepressant response with modest reliability [177]. GWAS to analyze the genetic basis of treatment-resistant depression and non-treatment-resistant depression with SSRIs, serotonin-norepinephrine reuptake inhibitors (SNRIs), and norepinephrine-dopamine reuptake inhibitors (NDRIs) identified three genomic regions (rs4884091, rs4955665, rs150245813) which associate with response to SSRIs and SNRIs [178]. *NRG1*, *DOCK10*, *GLS*, *PRPS1*, *TMEM161B*, *GLO1*, *FANCF*, *HNRNPDL*, *CD47*, *OLFM1*, *SMAD7*, and *SLC6A4* variants are major pathogenic genes of interest in depression [179]. Several SNPs located at candidate genes influence response to antidepressants (*COMT*, *HTR2A*, *HTR1A*, *CNR1*, *SLC6A4*, *NPY*, *MAOA*, *IL1B*, *GRIK4*, *BDNF*, *GNB3*, *FKBP5*, *CYP2D6*, *CYP2C19*, and *ABCB1*) and mood stabilizers (lithium) (*5-HTT*, *TPH*, *DRD1*, *FYN*, *INPP1*, *CREB1*, *BDNF*, *GSK3β*, *ARNTL*, *TIM*, *DPB*, *NR3C1*, *BCR*, *XBP1*, and *CACNG2*) [19,180]. 

Sertraline is a major substrate of CYP2C19 enzymes. In dose-harmonized serum concentrations of sertraline and N-desmethylsertraline-to-sertraline metabolic ratio, the sertraline serum concentration is 1.38-fold higher in IMs and 2.68-fold in CYP2C19-PMs, with 10% lower serum concentration in CYP2C19-UMs. According to these results, dose reductions of 60% and 25% should be considered in PMs and IMs, respectively, to reduce sertraline overexposure [181].

The study of the two most common *CYP2C19* functional SNPs (rs4244285 and rs12248560) in the Caucasian population, determining the phenotypic conditions of NMs, IMs, PMs and UMs, helps to predict the therapeutic response and risk of ADRs in patients treated with citalopram or escitalopram. PMs show higher improved symptomatology symptom improvement and higher remission rates compared to NMs. Gastro-intestinal, neurological and sexual ADRs are also more frequent in PMs than in NMs [175,176]. Compared to CYP2C19*1/*1 NMs, the exposure to citalopram increases by 95% in PMs (CYP2C19*2, *3/*2, *3), by 30% in NM/PM (CYP2C19*1/*2, *3), and by 25 % in UM/PM (CYP2C19*17/*2, *3). In UMs (CYP2C19*17/*17), the exposure to citalopram decreases by 36%, and in UM/NMs (CYP2C19*17/*1) by 14% [182]. Consequent dose adjustment is recommended in PMs and UMs treated with citalopram or escitalopram [19]. 

*CYP2C19* variants (*2, *3, *4, *6, *8, and *17) affect the risk of ADRs in children treated with antidepressants. Surprisingly, sertraline- and citalopram-related ADRs appear to be more common in CYP2C19-NMs than in IMs or PMs [183].

PGx of psychotropic drugs is relatively well studied; however, its practical application is still deficient, due to poor understanding of PGx testing and direct benefits for the patients in terms of accurate prescription and reduction in potential ADRs [19,184,185,186].

## 6. Immunopharmacogenomics and Cutaneous ADRs

ADRs can be differentiated into on-target or off-target ADRs. On-target ADRs (>80%) are the direct consequence of drug-related properties and, consequently, are dose-dependent, genotype-related, and potentially predictable. In contrast, off-target ADRs are the result of pharmacological interactions with specific host cell factors which elicit an adaptive immune response in which a particular immunogenetic profile is potentially involved. Immune-mediated ADRs (IM-ADRs) can be classified according to the primary immune cell involved in (i) B-cell-mediated reactions (Gell-Coombs type I-III reactions), and (ii) T-cell-mediated reactions (Gell-Coombs type IV or delayed hypersensitivity). Skin IM-ADRs are mediated by T cells and can lead to a life-threatening cutaneous, systemic or organ reaction, such as drug-induced liver disease. T-cell-mediated ADRs are linked to specific HLA genotypes. Off-target ADRs may be associated with immunological memory, showing diverse phenotypes (maculopapular exanthema, severe skin ADRs, angioedema, pruritus, and bronchospasm) [187,188].

Immunopharmacogenomics integrates the disciplines of immunogenomics and pharmacogenomics with special focus in immune-specific variation on drug disposition and IM-ADRs, which represent one-fifth of ADRs [189,190]. Immune-mediated ADRs are responsible for over 20% of ADRs. *HLA* gene variation contributes to immune-specific variation on drug disposition and ADRs and it is useful in predictive immunopharmacogenomics [189,190].

Severe cutaneous adverse reactions (SCAR) (maculopapular eruptions; Stevens-Johnson syndrome, SJS; toxic epidermal necrolysis, TEN; drug rash with eosinophilia and systemic symptoms, DRESS) are unpredictable and idiosyncratic drug-hypersensitivity skin reactions with a high-mortality rate (10–40%) [14,191,192]. SJS and TEN are life-threatening disorders with detachment of the epidermis and mucous membrane. SJS/TEN may also cause liver, kidney, and lung complications. Predictive biomarkers of SJS/TEN include *HLA* genotyping prior to drug administration (e.g., carbamazepine, allopurinol, abacavir, and oseltamivir), apoptotic markers of the Fas-FasL or perforin/granzyme pathways, and necroptosis markers (CCL-27, IL-15, galectin-7, RIP3) [193].

Drug-related allergy is a hypersensitivity reaction activated by an IgE or T-cell-mediated mechanism. *HLA* gene variants influence the recognition of the drug by B and T cells. IgE-mediated mechanisms are associated with human leukocyte antigen presentation *(HLA A2*, *DRw52*), *TNFA* -308G>A, *IL-13* and *IL-4RA* variants, and expression of IgE receptors on target cells. HLA alleles also influence delayed T-cell-mediated reactions. Stevens-Johnson syndrome or toxic epidermal necrolysis in patients treated with carbamazepine are associated with *HLA*-B*1502 and HLA-B*5801; HLA-B*5701 associates with abacavir hypersensitivity in white HIV patients, but not in Hispanics or Africans [194].

Cutaneous ADRs exhibit an ethnic-related pattern, with higher prevalence in Asian countries (Chinese, Malays, and Indians) [195]. HLA-B*15:*02* and HLA-B*58:*01* are highly recommended for patients to be treated with carbamazepine and allopurinol, respectively. The *HLA*-B*58:*01* allele is associated with high risk of severe skin ADRs in the Han Chinese, Korean, Thai, Japanese and European populations treated with allopurinol [196,197]. Over 95% of patients with allopurinol-related cutaneous ADRs are carriers of the *HLA*-*B* (*) *58*:*01* allele [198].

Allopurinol-induced SCARs, including DRESS, SJS and TEN, are life-threatening autoimmune reactions influenced by DNA methylation. A genome-wide DNA methylation profiling study conducted in patients with allopurinol-induced SCARs identified 41 differentially methylated CpG loci annotated to 26 genes with altered DNA methylation. Significant DNA hypomethylation is observed in *PSORS1C1* (cg24926791) between allopurinol-tolerant and allopurinol-SCAR patients [199].

HLA tests show high specificity and negative predictive value (NPV) for predicting hypersensitivity reactions. The sensitivity of HLA tests ranges from 0–33% for *HLA*-B*1502 associated with lamotrigine-induced SJS/TEN to 100% for *HLA*-B*5701 associated with abacavir hypersensitivity syndrome (ABC-HSR) [200].

Although most studies have identified high-risk HLA allotypes associated with cutaneous ADRs, the presence of the HLA allotype at risk is not sufficient to elicit drug hypersensitivity. Insufficient regulation by regulatory T cells (Tregs) may play a role in severe hypersensitivity reactions. Immune checkpoint inhibitors (anti-CTLA-4 and anti-PD-1) in cancer treatment may also induce SJS/TEN and DRESS/DIHS and other hypersensitivity reactions [201].

SJS and TEN are rare ADRs with a polygenic background due to additive/epistatic effects caused by multilocative genetic variation. The first whole-exome sequencing (WES) of SJS-TEN patients identified combinations of frequently occurring and rare variants that contribute to the disease’s pathogenesis. *NAT2*, *CYP2D8*, *CYP2B6*, *ABCC2*, *UGT2B7* and *TCF3* variants are potential PGx markers of drug-specific SJS-TEN [202].

## 7. COVID-19

In a study of three genome-wide association meta-analyses with 49,562 coronavirus disease-19 (COVID-19) patients from 46 studies across 19 countries, 13 genome-wide significant loci associated with severe manifestations of COVID-19 have been identified. Nine genome-wide loci are related to moderate-severe hospitalized COVID-19. Loci (and proximity genes) associated with susceptibility to SARS-CoV-2 infection and severe COVID-19 phenotypes include the following: (i) rs2271616 (*SLC6A20* and *CCR3*), rs10490770 (*LZTFL1* and *CXCR6*) and rs11919389 (*RPL24*, *NXPE3*, *ZBTB11*, and *CEP97*) in chromosome 3; (ii) rs1886814 (*FOXP4*) in chromosome 6; (iii) rs72711165 (*TMEM65*) in chromosome 8; (iv) rs912805253 (*ABO*) in chromosome 9; (v) rs10774671 (*OAS1*, *OAS2*, and *OAS3*) in chromosome 12; (vi) rs77534576 (*TAC4*, *DLX3*, *FLJ45513*, and *KAT7*) and rs1819040 (*KANSL1*, *ARHGAP27*, *PLEKHM1*, *LINC02210*-*CRHR1*, *CRHR1*, *SPPL2C*, *MAPT*, *STH*, *LRRC37A*, *ARL17B*, *LRRC37A2*, *ARL17A*, *NSF*, and *WNT3*) in chromosome 17; (vii) rs4801778 (*PLEKHA4*, *TULP2*, *HSD17B14*, *PPP1R15A*, and *NUCB1*), rs109069 (*DPP9*) and rs74956615 (*RAVER1*, *TYK2*, *ICAM5*, *ICAM1*, *ICAM4*, *ZGLP1*, *FDX2*, and *ICAM3*) in chromosome 19; and (viii) rs13050728 (*IFNAR2*) in chromosome 21. The strongest and most robust finding for severity is at locus 3p21.31 [203].

Simulation studies with multigene PGx testing in individuals hospitalized with COVID-19 in the United States identified 14 genes (*CYP2C19*, *CYP2C9*, *CYP2D6*, *CYP3A5*, *DPYD*, *G6PD*, *HLA-A*, *HLA-B*, *IFNL3*, *NUDT15*, *SLCO1B1*, *TPMT*, *UGT1A1*, and *VKORC1*) with potential utility for optimizing therapeutics in patients with coronavirus infection. Nearly 20% of the patients would receive safer medication if PGx interventions had been applied. *CYP2D6* and *CYP2C19* variants are responsible for the majority of treatment modifications [204] (Table 4).

Older-aged adults are at highest risk for severe COVID-19-associated outcomes. Extremes of age are associated with increased expression of selected pattern recognition receptors (PRR) genes (*TLR3*, *TLR4*, and *IHIF1*), *ACE2* and four genes (*DAM9*, *FBLN5*, *FAM8A1*, and *CLIP4*) that encode proteins that interact with SAR2-CoV-2 proteins [205].

SARS-CoV-2 uses ACE2 as host receptor and host proteases for cell surface binding and internalization. Some *ACE2* variants [p.(Asn720Asp), p.(Lys26Arg), and p.(Gly211Arg)] interfere with protein structure and stabilization and other rare *ACE2* variants [p.(Leu351Val) and p.(Pro389His)] interfere with SARS-CoV-2 spike protein binding. *ACE2* variants contribute to interindividual variability (predisposition and clinical features) associated with COVID-19 [206]. *ACE2* c.2158A>G p.(Asn720Asp), c.77A>G p.(Lys26Arg), c.631G>A p.(Gly211Arg) are the three more common variants. Since the *ACE2* gene localizes on the X chromosome, most women are heterozygotes for the c.2158A>G p.(Asn720Asp) variant, which is absent in the Eastern Asia population. In the European population, homozygous women for the c.77A>G p.(Lys26Arg) variant are very rare and no homozygous females were reported for the c.631G>A p.(Gly211Arg). Other 28 rare missense variants were identified [206]. Profound differences in the distribution and frequency of *ACE2* variants have been demonstrated in different continents and ethnic groups [207,208]. Sex differences are also relevant, probably justifying a higher mortality rate in men than in women. The sex discordance in COVID-19 outcomes is potentially link to androgen-induced expression of *TMPRSS2* and/or *ACE2* in pulmonary tissues which increases susceptibility or severity in males [208,209,210]. Lung *TMPRSS2* expression is higher in males than females, and *ACE2* and *AR* expression is sexually dimorphic and higher in males than females [211]. The level and expression pattern of *ACE2* in tissues and cells are critical to the susceptibility and symptoms resulting from SARS-CoV-2 infection. *ACE2* and *TMPRSS2* are key players on SARS-CoV-2 entry into host cells. *ACE2* and *TMPRSS2* levels correlate with age and are strongly associated with respiratory distress. Increased nasopharyngeal *ACE2* levels are protective and the *TMPRSS2*/*ACE2* ratio is associated with risk of COVID-19 with prevalent symptomatology (cough, 70.42%; fever, 63.85%; headache, 61.50%; anosmia, 57.28%; myalgia, 55.87%) [212]. The imbalance between *ACE1* and *ACE2* activity is implicated in the pathogenesis of respiratory disorders and in COVID-19 severity. Severe COVID-19 is associated with hypertension, hypercholesterolemia, and the *ACE1*-*DD* genotype [213]. SARS-CoV-2 infection is relatively frequent in the elderly with co-morbidities (metabolic, cardiovascular, neoplastic, brain disorders) in whom low *ACE2* levels may reduce the potential risk of COVID-19 [214].

At the EuroEspes International Center of Neurosciences and Genomic Medicine, we have developed the COVID-19 GenoPredictor for (i) prediction of risk for COVID-19 and COVID-19-related pulmonary disorder, and (ii) personalized treatments in hospitalized and ambulatory patients treated with conventional drugs and polypharmacy when affected by COVID-19 (Table 4). Current therapeutic strategies used in different countries include hydroxychloroquine, ceftriaxone, moxifloxacin/ciprofloxacin, levofloxacin, ondansetron, enoxaparin and corticoids [206]. The COVID-19 GenoPredictor includes several categories of genes: (i) genes associated with COVID-19 and respiratory disease (*ACE2* rs2285666; *TPMRSS2* rs2070788), (ii) genes associated with vascular risk (*ACE1* rs4332; *ACE2* rs2285666; *AGT* rs4762, rs699), thromboembolic risk (*F2* rs1799963; *F5* rs6025), immune response (*IL1B* rs1143634; *IL6* rs1800795; *IL6R* rs693; *TNF* rs708272), and metabolism (*MTHFR* rs1801133); and (iv) genes involved in the metabolism (Phase-I (*CYP2C9*, *CYP2C19*, *CYP2D6*, *CYP3A4*, and *CYP3A5*) and Phase-II reactions (*NAT2*)) and transport (*ABCB1*, *ABCC2*, and *SLCO1B1*) of drugs commonly used in patients with COVID-19. 

In the Spanish population, subjects with risk (*ACE2*-G) represent more than 90% of the population, with higher risk in males than in females (X^2^ = 0.225; *p* = 0.61). The frequency of *ACE2* genotypes in females are the following: *ACE2*-A/A 8.33%; *ACE2*-A/G 22.22%; and *ACE2*-G/G 69.45%; and in males, *ACE2*-A 12.20% and *ACE2*-G 87.80%. 

The frequency of the *TMPRSS2* genotypes in the population is *TMPRSS2*-C/C 27.27%, *TMPRSS2*-C/T 51.95% and *TMPRSS2*-T/T 20.78%, with higher risk (*TMPRSS2*-C) in males than in females (X^2^ = 0.211, *p* = 0.64). By sexes, in females *TMPRSS2*-C/C 22.22%, *TMPRSS2*-C/T 52.95% and *TMPRSS2*-T/T 20%; and in males, *TMPRSS2*-C/C 31.71%, *TMPRSS2*-C/T 51.22%, and *TMPRSS2*-T/T 17.07%.

*ACE1* variants in the population are *ACE1*-C/C 33.77%, *ACE1*-C/T 37.66% and *ACE1*-T/T 28.57%, with higher frequency of the allele of risk (*ACE*1-T) in males than in females (F-*ACE1*-C/C 38.89%; F-*ACE1*-C/T 44.44%; F-*ACE1*-T/T 16.67%; M-*ACE1*-C/C 29.27%; M-*ACE1*-C/T 31.71%; M-*ACE1*-T/T 30.02%; X^2^ = 1.92, *p* = 0.16).

Small differences in the frequency of *IL1B*, *IL6* and *IL6R* are seen between females and males without apparent significance. Homozygous males for the genotype of risk *IL6*-C573C are very rare and heterozygous C573G cases are more frequent in women (25%) than in males (7%). In contrast, the wild-type genotype (*IL6*-G573G) is more frequent in males (93%) than in females (72%). This variant is associated with brain thromboembolic infarcts and cerebral hemorrhage [215].

In COVID-19 patients with severe respiratory syndrome and co-morbidities caution should be taken with polypharmacy. Drug-induced interstitial lung disease is the consequence of specific chemical properties of a given drug, DDIs and PGx factors. The principles of dose adjustment according to the condition of NM, IM, PM and UM are similar to those adopted to optimize therapeutics in any other circumstance with similar drugs [19,216].

PGx data are relevant for nearly all individuals hospitalized with COVID-19, providing the opportunity to improve clinical care in these patients as well as for the appropriate selection of specific SARS-CoV-2 vaccines to minimize potential ADRs. Historically, the occurrence of ADRs in people inoculated with identical vaccines was interpreted as unpredictable stochastic processes. However, PGx studies showed that host response to immunization is highly influenced by genomic factors opening the avenue of personalized vaccinology [217].

## 8. Future Trends

Major limitations for the routine use of PGx procedures are the lack of education and training in physicians and pharmacists (<5% of physicians are familiar with PGx), poor characterization of drug-related PGx (<30% of drugs are appropriately studied), unspecific biomarkers of drug efficacy and safety, cost-effectiveness, administrative problems in health organizations, and insufficient regulation for the generalized use of PGx in the clinical setting [11,12,19,27,218,219,220]. Studies exploring the views of the general public regarding use of medications, side effects and PGx indicate that the major concerns of the public for the implementation of PGx are poor level of information and ineffective communication from their health-care professionals, lack of PGx education of clinicians and pharmacists, storage and privacy of genetic information, costs, insurance coverage and employment discrimination [221,222,223,224,225]. Although the attitude of the public differs from country to country, in general, the vast majority shows a positive attitude towards PGx (>80%), especially those patients who experience ADRs [226]. PGx education is quite variable depending upon country, institutions, medical specialty and age among physicians. Pediatric clinicians (60%) are more confident than adult clinicians (18%) with the use of genetic information and PGx in medical care [227]. Clinician attitudes toward PGx are vital to the success of translational programs. Although, on a theoretical basis, most clinicians might agree on the convenience and value of PGx testing, clinicians do not agree on how to assign clinical responsibility for actionable results from a PGx panel [228]. A multidisciplinary, cultural approach would be convenient for the gradual introduction of PGx into daily clinical practice in order to overcome resistance in a sector of the medical community unfamiliar with genomics.

In terms of cost-effectiveness, there is evidence supporting the predictive testing of *HLA*-B*57:*01* for abacavir, *HLA*-B*15:*02* and *HLA*-A*31:*01* for carbamazepine, *HLA*-B*58:*01* for allopurinol and CYP2C19 for clopidogrel treatment. Other potential markers are *TPMT* genotyping prior to 6-mercaptoputine, azathioprine and cisplatin therapy, *CYP2C9* and *VKORC1* for dosing of coumarin derivatives, *MTHFR* prior to methotrexate treatment, *factor V Leiden* prior to oral contraception, and *CYP2D6* variation for optimizing psychotropic drug prescription [185,229,230,231]. Despite these apparently cost-effective geno-markers for a reduced number of drugs, there are clear data demonstrating that the characterization of pathogenic, mechanistic, metabolic, transporter and pleiotropic genes are undoubtedly beneficial for optimizing the treatment of chronic disorders, helping physicians in decision-making for drug selection, dosing accuracy, and ADRs minimization [11,12,19,27,232,233]. However, predictive genetic testing is rare (4.4% in Medicaid and 10.5% in Medicare cohorts) despite FDA pharmacogenomic labeling information [234]. In terms of PGx recommendations, discrepancies remain among the U.S. Food and Drug Administration (FDA), the European Medicine Agency (EMA), and the Clinical Pharmacogenetics Implementation Consortium (CPIC) [235]. Clinically validated PGx information is included in some drug labels; however, drug label information differs from country to country. This lack of harmonization is attributed to differences in population allele frequencies, variable genetic test availability and diversity in insurance coverage [236]. The annual proportion of new FDA drug approvals with PGx labeling increased by nearly 3-fold from 10.3% in 2000 to 28.2% in 2020, especially in cancer drugs (75.5%) [237].

The current regulatory framework applicable to PGx tests is different in Europe, USA, Canada, and some Asian countries (China, Japan) [19,238,239]. The US FDA has supported PGx for nearly two decades by providing regulatory advice and reviewing applications, developing policies on genomics and individualized therapeutics for rational drug development, and informing the public of clinically relevant PGx issues, including drug labeling [240]. However, other regulatory agencies in different countries did not follow a similar strategy, with the consequent delay in the implementation of PGx procedures both in the development of new drugs and in the optimization of the use of current drugs to reduce ADRs [19].

PGx is probably the optimum approach to optimize pharmacotherapy by reducing ADRs and improving drug efficacy and safety. Pre-emptive PGx profiling may be highly relevant for in-hospital prescribing in order to reduce ADRs, especially in elderly patients and in those with polypharmacy [241]. PGx testing of polypharmacy patients (>50 yrs) may considerably reduce re-hospitalizations and ADRs resulting in health resource utilization savings and improved health care [242].

Important steps for the implementation of PGx procedures in the clinical setting include: (i) education of physicians and all other parties involved in the use and benefits of PGx [243,244]; (ii) performance of prospective studies to demonstrate the benefits of PGx genotyping [245,246]; (iii) standardization of PGx procedures and development of clinical guidelines [243,247]; (iv) incorporation of commercially available genome-wide association sequencing chips and microarrays to cover genes with high PGx potential (ADME genes, pathogenic genes) [248,249]; (v) implantation of new regulations for PGx-related drug development and PGx drug labelling [240,250,251]; and (vi) creation of national PGx boards for supervision of the correct application of personalized treatments, considering ethical, legal, ethnic, and age- and sex-related issues [252,253].

To begin this process of changing habits and prescriptive mindset, it would not hurt to remember what George Bernard Shaw wrote in his *Maxims for Revolutionaries* in *Man and Superman* (1903): “The reasonable man adapts himself to the world; the unreasonable one persists in trying to adapt the world to himself. Therefore, all progress depends on the unreasonable man”; and, above all, not to forget what Norbert Wiener said in *The Human Use of Human Beings* (1954) “Progress imposes not only new possibilities for the future but new restrictions”.

## 9. Conclusions

ADRs rank as one of the top 10 leading causes of death and illness in developed countries, with direct medical costs of over $150 billion/year only in the USA. ADRs show differential features depending upon genotype, age, sex, race, pathology, drug category, route of administration, and drug–drug interactions. PGx accounts for 20–95% of drug response variability, with a significant role in the incidence and severity of ADRs. Differential PGx features in children, aged patients, and women deserve special attention for preventing ADRs; and caution should be taken when extrapolating data from clinical trials performed in Caucasians to other populations due to ethnic-related PGx differences. Novel data on the PGx of drugs for the treatment of cardiovascular disease and related disorders, cancer and CNS disorders are of help in the clinical setting for optimizing therapeutics and reducing ADRs. The PGx and pharmacological properties of agents acting on the renin–angiotensin system (ACE inhibitors, angiotensin II antagonists) and other antihypertensive drugs, β-blocking agents, calcium-channel blockers, cardiotonic agents, and diuretics (Table 1), lipid-modifying agents (Table 2), antithrombotic drugs, including vitamin K antagonists, heparins, platelet aggregation inhibitors, direct thrombin inhibitors, and direct factor Xa inhibitors (Table 3), provide information on pathogenic, mechanistic, metabolic, transporter and pleiotropic genes of interest for the correct use of these drugs. Over 100 ADRs are preventable in patients with cancer treated with pharmacogenetically actionable medications. Predictive testing of *HLA*-B*57:*01* for abacavir, *HLA*-B*15:*02* and *HLA*-A*31:*01* for carbamazepine, *HLA*-B*58:*01* for allopurinol, *CYP2C19* for clopidogrel, *TPMT* for 6-mercaptoputine, azathioprine and cisplatin therapy, *CYP2C9* and *VKORC1* for coumarin derivatives, *MTHFR* for methotrexate treatment, *factor V Leiden* for oral contraception, and *CYP2D6* for psychotropic drug prescription is cost-effective. Thirteen genome-wide significant loci associated with severe manifestations of COVID-19 have been identified. Multigene PGx testing in individuals hospitalized with COVID-19 identified fourteen genes with potential utility for optimizing therapeutics in patients with coronavirus infection. PGx is relevant for nearly all individuals with COVID-19, and for the appropriate selection of specific SARS-CoV-2 vaccines to minimize potential ADRs. Clinician attitudes toward PGx are vital to the success of translational programs. PGx provides the physician effective clues for optimizing drug efficacy and safety in the treatment of major problems of health.

## Figures and Tables

**Table 1:** Pharmacogenetics of selected drug categories with effects on the cardiovascular system.

Agents Acting on the Renin–Angiotensin System		
ACE Inhibitors		
Drug	Properties	Pharmacogenetics
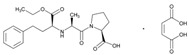	**Name: ENALAPRIL** **IUPAC Name:** L-proline, 1-[N-[1-(ethoxycarbonyl)-3-phenylpropyl]-L-alanyl]-, (S)-, (Z)-2-butenedioate (1:1); 1-[N-[(S)-1-carboxy-3-phenylpropyl]-L-alanyl]-L-proline 1′-ethyl ester, maleate (1:1) **Molecular Formula:** C_20_H_28_N_2_O_5_ C_4_H_4_O_4_ **Molecular Weight:** 492.52 **Mechanism:** Competitive inhibitor of angiotensin-converting enzyme (ACE). Prevents conversion of angiotensin I to angiotensin II, a potent vasoconstrictor. Results in lower levels of angiotensin II, which causes an increase in plasma renin activity and a reduction in aldosterone secretion. **Effect:** Treatment of hypertension, symptomatic heart failure, or asymptomatic left ventricular dysfunction.	**Mechanistic genes:***ACE1*, *ADRB2*, *AGTR1*, *AGT*, *BDKRB2*, *NOS3* **Metabolic genes** **Substrate:***CYP3A4*, *CYP3A5* **Inhibitor:***ACE1* **Transporter genes:***SLC22A8* **Pleiotropic genes:***IL6*
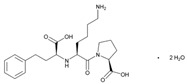	**Name: LISINOPRIL** **IUPAC Name:** L-proline, 1-[N2-(1-carboxy-3-phenylpropyl)-L-lysyl]-, dihydrate, (S)-; 1-[N2-[(S)-1-carboxy-3-phenylpropyl]-L-lysyl]-L-proline dihydrate **Molecular Formula:** C_21_H_31_N_3_O_5_ 2H_2_O **Molecular Weight:** 441.52 **Mechanism:** Competitive inhibitor of angiotensin-converting enzyme (ACE). Prevents conversion of angiotensin I to angiotensin II, a potent vasoconstrictor. **Effect:** Treatment of hypertension, either alone or in combination with other antihypertensive agents. Adjunctive therapy in treatment of heart failure. Treatment of acute MI within 24 h in hemodynamically stable patients to improve survival.	**Mechanistic genes:***ACE1*, *ADD1*, *AGTR1*, *AGT* **Metabolic genes** **Substrate:***ACE1*, *ADD1*, *AGTR1*, *AGT* **Inhibitor:***ACE1*, *ACE2*
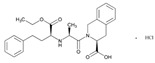	**Name: QUINAPRIL** **IUPAC Name:** 3-Isoquinolinecarboxylic acid, 2-[2-[[1-(ethoxycarbonyl)-3-phenylpropyl]amino]-1-oxopropyl]-1,2,3,4-tetrahydro-, monohydrochloride, [3S-[2[R*(R*)],3R*]]; (S)-2-[(S)-N-[(S)-1-carboxy-3-phenylpropyl]alanyl]-1,2,3,4-tetrahydro-3-isoquinolinecarboxylic acid, 1-ethyl ester, monohydrochloride **Molecular Formula:** C_25_H_30_N_2_O_5_ HCl **Molecular Weight:** 474.98 **Mechanism:** Treatment of hypertension. Reduction in cardiovascular mortality or non-fatal myocardial infarction in stable coronary artery disease. **Effect:** Hypertension. Heart failure.	**Mechanistic genes:** *ACE1*, *AGT*, *AGTR1*, *BDKBR2*, *NR1I21 TGFB1* **Metabolic genes** **Substrate:** *CYP11B2* **Inhibitor:** *ACE1*
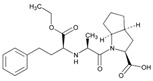	**Name: RAMIPRIL** **IUPAC Name:** [1,1′-Biphenyl]-2-carboxylic acid, 4′-[(1,4′-dimethyl-2′-propyl[2,6′-bi-1H-benzimidazol]-1′-yl)methyl]-; 4′-[[4-methyl-6-(1-methyl-2-benzimidazolyl)-2-propyl-1-benzimidazolyl]methyl]-2-biphenylcarboxylic acid **Molecular Formula:** C_23_H_32_N_2_O_5_ **Molecular Weight:** 514.62 **Mechanism:** A non-peptide AT1 angiotensin II receptor antagonist. This binding prevents angiotensin II from binding to receptor thereby blocking vasoconstriction and aldosterone-secreting effects of angiotensin II. **Effect:** Treatment of hypertension, alone or in combination with other antihypertensive agents.	**Mechanistic genes:** *ACE1*, *AGT*, *AGTR1*, *BDKRB2*, *ERAP1*, *PPARG* **Metabolic genes** **Substrate:** *CYP2C9*, *CYP11B2*, *UGT1A1* **Inhibitor:** *ABCB1*, *ABCG2*, *CYP2C9*, *CYP2C19*
**Angiotensin II Antagonists**		
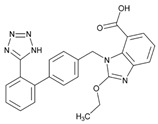	**Name: CANDESARTAN** **IUPAC Name:** 1H-benzimidazole-7-carboxylic acid, 2-ethoxy-1-[[2′-(1H-tetrazol-5-yl)[1,1′-biphenyl]-4-yl]methyl]-; 2-ethoxy-1-[p-(o-1H-tetrazol-5-ylphenyl)benzyl]-7-benzimidazolecarboxylic acid **Molecular Formula:** C24H20N6O3 **Molecular Weight:** 440.45 **Mechanism:** Candesartan is an angiotensin receptor antagonist, blocking vasoconstriction and the aldosterone-secreting effects (reabsorption of sodium and water) of angiotensin II. **Effect:** Essential hypertension. Heart failure.	**Metabolic genes** **Substrate:** *CYP1A1*, *CYP2C9*, *CYP11B2*, *UGT1A3*, *UGT1A5*, *UGT2B7* **Inhibitor:** *ABCG2*, *CYP2C8*, *CYP2C9* **Transporter genes:***ABCB1*, *ABCG2*
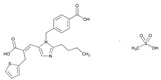	**Name: EPROSARTAN** **IUPAC Name:** 2-Thiophenepropanoic acid, α-[[2-butyl-1-[(4-carboxyphenyl)methyl]-1H-imidazol-5-yl]methylene]-, €-, monomethanesulfonat€(E)-2-butyl-1-(p-carboxybenzyl)-α-2-thenylimidazole-5-acrylic acid, monomethanesulfonate **Molecular Formula:** C_23_H_24_N_2_O_4_S CH_4_O_3_S **Molecular Weight:** 520.62 **Mechanism:** A non-biphenyl, non-tetrazole angiotensin II receptor (AT1) antagonist. Blocks the vasoconstrictor and aldosterone-secreting effects of angiotensin II by selectively blocking the binding of angiotensin II to the AT1 receptor in many tissues, such as vascular smooth muscle and adrenal gland. Does not bind to or block other hormone receptors or ion channels known to be important in cardiovascular regulation. **Effect:** Used alone or in combination with other classes of antihypertensive agents in the management of hypertension.	**Mechanistic genes:** *ACE1*, *AGTR1* **Metabolic genes** **Inhibitor:** *CYP2C9* **Inducer:** *ABCC2* **Transporter genes:** *ABCB1*, *ABCG2*
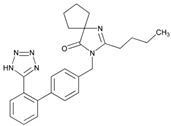	**Name: IRBESARTAN** **IUPAC Name:** 1,3-Diazaspiro[4.4]non-1-en-4-one, 2-butyl-3-[[2′-(1H-tetrazol-5-yl)[1,1′-biphenyl]-4-yl]methyl]-; 2-butyl-3-[p-(o-1H-tetrazol-5-ylphenyl)benzyl]-1,3-diazaspiro[4.4]non-1-en-4-one **Molecular Formula:** C_25_H_28_N_6_O **Molecular Weight:** 428.53 **Mechanism:** Irbesartan binds to AT1 angiotensin II receptor. This binding prevents angiotensin II from binding to receptor, thereby blocking the vasoconstriction and aldosterone-secreting effects of angiotensin II. **Effect:** Treatment of hypertension alone or in combination with other antihypertensives. Treatment of diabetic nephropathy in type 2 diabetes mellitus (non-insulin-dependent, NIDDM) and hypertension.	**Mechanistic genes:** *ADRA1A*, *AGTR1*, *APOB*, *BDKRB2*, *ERAP1*, *EDN1*, *NPPA*, *AGT*, *APOE*, *LDLR*, *NOS3*, *TGFB1* **Metabolic genes** **Substrate:** *CYP2C9*, *CYP3A4*, *CYP3A5*, *CYP11B2* **Inhibitor:** *CYP1A2*, *CYP2C8*, *CYP2C9*, *CYP2D6*, *CYP3A4*, *CYP3A5* **Transporter genes:** *ABCB1*, *ABCG2*
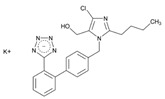	**Name: LOSARTAN** **IUPAC Name:** 1H-Imidazole-5-methanol, 2-butyl-4-chloro-1-[[2′-(1H-tetrazol-5-yl)[1,1′-biphenyl]-4-yl]methyl]-, monopotassium salt; 2-butyl-4-chloro-1-[p-(o-1H-tetrazol-5-ylphenyl)benzyl]imidazole-5-methanol, monopotassium salt **Molecular Formula:** C_22_H_22_ClKN_6_O **Molecular Weight:** 461.00 **Mechanism:** As a selective and competitive non-peptide angiotensin II receptor antagonist, losartan blocks vasoconstrictor and aldosterone-secreting effects of angiotensin II. Losartan increases urinary flow rate and in addition to being natriuretic and kaliuretic, increases excretion of chloride, magnesium, uric acid, calcium, and phosphate. **Effect:** Treatment of hypertension. Treatment of diabetic nephropathy in type 2 diabetes mellitus (non-insulin-dependent) and history of hypertension. Stroke risk reduction in hypertension and left ventricular hypertrophy.	**Mechanistic genes:***ACE1*, *ADD1*, *AGT*, *AGTR1*, *AGTR2*, *BDKRB2*, *EDN1*, *FOS*, *MMP2*, *NOS3*, *PDGFRB*, *REN*, *TGFB1* **Metabolic genes** **Substrate:***CYP1A2*, *CYP2C8*, *CYP2C9*, *CYP2C19*, *CYP2D6*, *CYP3A4*, *CYP3A5*, *UGT1A1*, *UGT1A3*, *UGT1A10*, *UGT2B7*, *UGT2B17* **Inhibitor:***CYP1A2*, *CYP2C8*, *CYP2C9*, *CYP2C19*, *CYP3A4*, *CYP3A5*, *CYP11B2* **Transporter genes:***ABCB1*, *ABCG2* **Pleiotropic genes:***TNF*
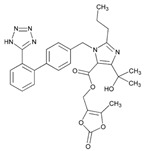	**Name: OLMESARTAN** **IUPAC Name:** 1H-Imidazole-5-carboxylic acid, 4-(1-hydroxy-1-methylethyl)-2-propyl-1-[[2′-(1H-tetrazol-5-yl) [1,1′-biphenyl]-4-yl]methyl]-, (5-methyl-2-oxo-1,3-dioxol-4-yl) methyl ester **Molecular Formula:** C_29_H_30_N_6_O_6_ **Molecular Weight:** 558.59 **Mechanism:** Blocks vasoconstrictor and aldosterone-secreting effects of angiotensin II. Interacts reversibly at AT1 and AT2 receptors and has slow dissociation kinetics (has greater affinity for AT1 receptor). Olmesartan increases urinary flow rate and, besides being natriuretic and kaliuretic, increases excretion of chloride, magnesium, uric acid, calcium, and phosphate. **Effect:** Hypertension.	**Mechanistic genes:***ACE2*, *AGTR1*, *EDN1*, *TGFB1* **Metabolic genes** **Substrate:***CMBL*, *CYP2C9* **Inducer:***ABCC2* **Transporter genes:***ABCB1*, *ABCC2*, *ABCG2*, *SLC22A8*, *SLCO1A2*, *SLCO1B1* **Pleiotropic genes:***APOE*
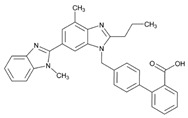	**Name: TELMISARTAN** **IUPAC Name:** [1,1′-Biphenyl]-2-carboxylic acid, 4′-[(1,4′-dimethyl-2′-propyl[2,6′-bi-1H-benzimidazol]-1′-yl)methyl]-; 4′-[[4-methyl-6-(1-methyl-2-benzimidazolyl)-2-propyl-1-benzimidazolyl]methyl]-2-biphenylcarboxylic acid **Molecular Formula:** C_33_H_30_N_4_O_2_ **Molecular Weight:** 514.62 **Mechanism:** A non-peptide AT1 angiotensin II receptor antagonist. This binding prevents angiotensin II from binding to receptor thereby blocking vasoconstriction and aldosterone-secreting effects of angiotensin II. **Effect:** Treatment of hypertension, alone or in combination with other antihypertensive agents.	**Mechanistic genes:***ACE1*, *AGT*, *AGTR1*, *BDKRB2*, *ERAP1*, *PPARG* **Metabolic genes** **Substrate:***CYP2C9*, *CYP11B2*, *UGT1A1* **Inhibitor:***ABCB1*, *ABCG2*, *CYP2C9*, *CYP2C19*
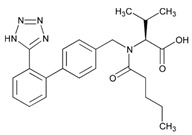	**Name: VALSARTAN** **IUPAC Name:** L-valine, N-(1-oxopentyl)-N-[[2′-(1H-tetrazol-5-yl)[1,1′-biphenyl]-4-yl]methyl]-; N-[p-(o-1H-tetrazol-5-ylphenyl)benzyl]-N-valeryl-L-valine **Molecular Formula:** C_24_H_29_N_5_O_3_ **Molecular Weight:** 435.52 **Mechanism:** Displaces angiotensin II from AT1 receptor and produces its blood pressure-lowering effects by antagonizing AT1-induced vasoconstriction, aldosterone release, catecholamine release, arginine vasopressin release, water intake, and hypertrophic responses. **Effect:** Treatment of essential hypertension (alone or in combination with other antihypertensive agents). Reduction in cardiovascular mortality in left ventricular dysfunction postmyocardial infarction. Treatment of heart failure.	**Mechanistic genes:***ACE1*, *AGT*, *AGTR1*, *AGT2R1*, *BDKRB2*, *ERAP1*, *GNB3*, *STAT3*, *TGFB1* **Metabolic genes** **Substrate:***CYP2C9*, *CYP2C19*, *CYP2D6*, *CYP3A4*, *CYP3A5*, *CYP11B2* **Inhibitor:***CYP2C9* **Transporter genes:***ABCC2*, *SLCO1B1*, *SLCO1B3*
**Other Agents acting on the Renin–Angiotensin system**		
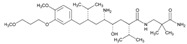	**Name: ALISKIREN** **IUPAC Name:** Benzeneoctanamide, δ-amino-N-(3-amino-2,2-dimethyl-3-oxopropyl)-γ-hydroxy-4-methoxy-3-(3-methoxypropoxy)-α,ζ-bis(1-methylethyl)-, (αS, γS, δS, ζS)-; (2) (2S,4S,5S,7S)-5-amino-N-(2-carbamoyl-2-methylpropyl)-4-hydroxy-2-isopropyl-7-[4-methoxy-3-(3-methoxypropoxy)benzyl]-8-methylnonamide **Molecular Formula:** C_30_H_53_N_3_O_6_ **Molecular Weight:** 551.76 **Mechanism:** Blocks conversion of angiotensinogen to angiotensin I. **Effect:** Treatment of hypertension.	**Mechanistic genes:***REN* **Metabolic genes** **Substrate:***CYP3A4*, *CYP3A5* **Inhibitor:***CYP3A4*, *CYP3A5*, *REN* **Transporter genes:***ABCB1* **Mechanistic genes:***ACE1*, *ACE2*, *ADD1*, *ADRB2*, *AGT*, *AGTR1*, *MTHFR*, *MTR*
**Antihypertensives**		
**Drug**	**Properties**	**Pharmacogenetics**
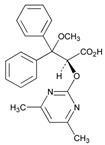	**Name: AMBRISENTAN** **IUPAC Name:** (+)-(2S)-2-[(4,6-dimethylpyrimidin-2-yl)oxy]-3-methoxy-3,3-diphenylpropanoic acid **Molecular Formula:** C_22_H_22_N_2_O_4_ **Molecular Weight:** 378.42 **Mechanism:** Blocks endothelin receptor ETA and ETB on vascular endothelium and smooth muscle. **Effect:** Treatment of pulmonary artery hypertension.	**Mechanistic genes:***EDN1*, *EDNRA*, *NOS3* **Metabolic genes** **Substrate:***CYP2C9*, *CYP2C19*, *CYP3A4*, *CYP3A5*, *GSTs*, *UGT1A3*, *UGT1A9*, *UGT2B7* **Transporter genes:***ABCB1*, *SLCO1A2* **Pleiotropic genes:***IL1B*, *IL6*
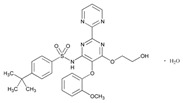	**Name: BOSENTAN** **IUPAC Name:** Benzenesulfonamide, 4-(1,1-dimethylethyl)-N-[6-(2-hydroxyethoxy)-5-(2-methoxyphenoxy)[2,2′-bipyrimidin]-4-yl]-, monohydrate; (2) p-tert-butyl-N-[6-(2-hydroxyethoxy)-5-(o-methoxyphenoxy)-2-(2-pyrimidinyl)-4-pyrimidinyl]benzenesulfonamide monohydrate **Molecular Formula:** C_27_H_29_N_5_O_6_S.H_2_O **Molecular Weight:** 569.63 **Mechanism:** Acts as a competitive antagonist and blocks endothelin receptors on vascular endothelium and smooth muscle. Stimulation of endothelin receptors is associated with vasoconstriction and proliferation. Although bosentan blocks both ETA and ETB receptors, the affinity is slightly higher for ETA. **Effect:** Adjunctive therapy for the treatment of pulmonary arterial hypertension (WHO group I), in patients with WHO class III or IV symptoms.	**Metabolic genes** **Substrate:***ACVRL1*, *BMPR2*, *EDNRA*, *EDNRB*, *TGFBR1* **Inhibitor:***CYP2B6*, *CYP2C9*, *CYP3A4*, *CYP3A5* **Inducer:***CYP2C9*, *CYP2C19*, *CYP3A4*, *CYP3A5* **Transporter genes:***ABCB1*, *ABCB11*, *SLCO1B1*, *SLCO1B3* **Pleiotropic genes:***TNF*
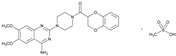	**Name: DOXAZOSIN** **IUPAC Name:** Piperazine, 1-(4-amino-6,7-dimethoxy-2-quinazolinyl)-4-[(2,3-dihydro-1,4-benzodioxin-2-yl)carbonyl]-, monomethanesulfonate; 1-(4-amino-6,7-dimethoxy-2-quinazolinyl)-4-(1,4-benzodioxan-2-ylcarbonyl)piperazine monomethanesulfonate **Molecular Formula:** C_23_H_25_N_5_O_5_.CH_4_O_3_S **Molecular Weight:** 547.58 **Mechanism:** Doxazosin is a quinazoline-derivative postsynaptic α1-adrenergic blocking agent. It reduces peripheral vascular resistance and blood pressure as a result of its vasodilating effects. The drug produces both arterial and venous dilation. Effects of doxazosin on the cardiovascular system are mediated by the drug’s activity at α1-receptor sites on vascular smooth muscle. Because of the prevalence of α receptors on the prostate capsule, prostate adenoma, and the bladder trigone and the relative absence of these receptors on the bladder body, α-blockers decrease urinary outflow resistance in men. Doxazosin may improve to a limited extent the serum lipid profile and can reduce blood glucose and serum insulin concentrations. The drug does not appear to affect plasma renin activity appreciably. **Effect:** Treatment of hypertension alone or in conjunction with diuretics, ACE inhibitors, β-blockers, or calcium antagonists. Treatment of urinary outflow obstruction and/or obstructive and irritative symptoms associated with BPH; can be used in combination with finasteride.	**Mechanistic genes:***ACE1*, *ADD1*, *ADRA1A* **Metabolic genes** **Substrate:***CYP2C19*, *CYP2D6*, *CYP3A4*, *CYP3A5* **Transporter genes:***ABCB1*
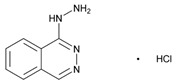	**Name: HYDRALAZINE** **IUPAC Name:** Phthalazine, 1-hydrazino-, monohydrochloride; 1-hydrazinophthalazine monohydrochloride **Molecular Formula:** C_8_H_8_N_4_ HCl **Molecular Weight:** 196.64 **Mechanism:** Direct vasodilation of arterioles (with little effect on veins) with decreased systemic resistance. **Effect:** Management of moderate-to-severe hypertension, congestive heart failure, hypertension secondary to pre-eclampsia/eclampsia. Treatment of primary pulmonary hypertension.	**Mechanistic genes:***AGPAT2*, *AGT*, *AKR1C4*, *CHRNA1*, *COL1A1*, *ESR1*, *GSTP1*, *HBB*, *HFE*, *HIF1A*, *MAOA*, *MGMT*, *NR3C1*, *PDGFRB* **Metabolic genes** **Substrate:***NAT2* **Inhibitor:***CEL*, *CYP3A4*, *CYP3A5* **Transporter genes:***SLC6A2*, *SLC12A3*, *SLC22A16* **Pleiotropic genes:***APC*, *HLA-A*, *HLA-B*, *IL6*, *IL10*, *TNF*, *TP53*
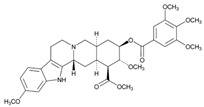	**Name: RESERPINE** **IUPAC Name:** Yohimban-16-carboxylic acid, 11,17-dimethoxy-18-[(3,4,5-trimethoxybenzoyl)oxy]-, methyl ester, (3β,16β,17α,18β,20α)-; methyl 18β-hydroxy-11,17α-dimethoxy-3β,20α-yohimban-16β-carboxylate 3,4,5-trimethoxybenzoate (ester) **Molecular Formula:** C_33_H_40_N_2_O_9_ **Molecular Weight:** 608.68 **Mechanism:** Reduces blood pressure via depletion of sympathetic biogenic amines (norepinephrine and dopamine). This also commonly results in sedative effects. **Effect:** Management of mild-to-moderate hypertension. Treatment of agitated psychotic states (schizophrenia).	**Mechanistic genes:***COMT*, *ERBB2*, *LDLR*, *MAOA*, *MAOB*, *NR1I2* **Metabolic genes** **Substrate:***CYP1A1*, *CYP3A4*, *CYP3A5*, *CYP7A1*, *UGT1A1* **Inhibitor:***ABCB1*, *ABCG2* **Inducer:***ABCB1* **Transporter genes:***ABCB11*, *SLC18A2*
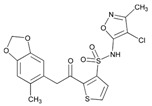	**Name: SITAXENTAN** **IUPAC Name:** N-(4-chloro-3-methyl-5-isoxazolyl)-2-[[4,5-(methylenedioxy)-o-toly]acetyl]-3-thiophenesulfonamide **Molecular Formula:** C_18_H_15_ClN_2_O_6_S_2_ **Molecular Weight:** 454.90 **Mechanism:** A selective antagonist of A subtype of endothelin-1 receptors (ETA) located in pulmonary smooth muscle. Stimulation of these receptors by endogenous endothelin-1 causes vasoconstriction. Sitaxsentan exhibits 6500-fold greater selectivity for ETA over the ETB subtype; the latter predominates on vascular endothelial cells. Thus, preferential antagonism of ETA reduces vasoconstriction, without compromising vasodilatory/antiproliferative actions mediated through endothelin-1 binding to ETB subtype. **Effect:** Primary pulmonary arterial hypertension or pulmonary hypertension secondary to connective tissue disease.	**Mechanistic genes:***EDNRA*, *EDNRB* **Metabolic genes** **Substrate:***CYP2C9*, *CYP3A4*, *CYP3A5* **Inhibitor:***CYP2C9*, *CYP2C19*, *CYP3A4*, *CYP3A5* **Transporter genes:***ABCB1*
**Beta Blocking Agents**		
**Drug**	**Properties**	**Pharmacogenetics**
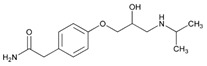	**Name: ATENOLOL** **IUPAC Name:** Benzeneacetamide, 4-[2-hydroxy-3-[(1-methylethyl)amino]propoxy]-; 2-[p-[2-hydroxy-3-(isopropylamino)propoxy]phenyl]acetamide **Molecular Formula:** C_14_H_22_N_2_O_3_ **Molecular Weight:** 266.34 **Mechanism:** Competitively blocks response to β-adrenergic stimulation, selectively blocks β1 receptors with little or no effect on β2 receptors except at high doses. **Effect:** Treatment of hypertension. Management of angina pectoris, postmyocardial infarction.	**Mechanistic genes:***ACE1*, *ACE2*, *ADRB1*, *ADRB2*, *AGT*, *APOB*, *BDKRB2*, *EDN1*, *ERAP1*, *GNAS*, *GNB3*, *GRK5*, *LDLR* **Metabolic genes** **Substrate:***CYP2C9*
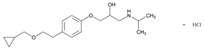	**Name: BETAXOLOL** **IUPAC Name:** 2-Propanol, 1-[4-[2-(cyclopropylmethoxy)ethyl]phenoxy]-3-[(1-methylethyl)amino]-, hydrochloride, (±)-; (2)(±)-1-[p-[2-(cyclopropylmethoxy)ethyl]phenoxy]-3-(isopropylamino)-2-propanol hydrochloride **Molecular Formula:** C_18_H_29_NO_3_ HCl **Molecular Weight:** 343.89 **Mechanism:** Competitively blocks β1 receptors, with little or no effect on β2 receptors (bronchial and vascular smooth muscle; only at high doses). No intrinsic sympathomimetic activity and little or no membrane-stabilizing effect (local anesthetic) on the heart. Reduces blood pressure by decreasing cardiac output, decreasing sympathetic outflow from the CNS, and/or suppressing renin release. Reduces intraocular pressure by reducing the production of aqueous humor (may block endogenous catecholamine-stimulated increases in cyclic adenosine monophosphate concentrations within the ciliary processes and subsequent formation of aqueous humor). **Effect:** Reduction in elevated intraocular pressure in chronic open-angle glaucoma and ocular hypertension (has been used effectively in glaucoma following laser trabeculoplasty). Reduction in systemic hypertension. Initial management of hypertension in heart failure, postmyocardial infarction, high coronary disease risk, and/or diabetes mellitus.	**Mechanistic genes:***ADRB1*, *ADRB2*, *AGT*, *BDKRB2*, *GNAS* **Metabolic genes** **Substrate:***CYP1A2*, *CYP2D6* **Inhibitor:***CYP2D6*
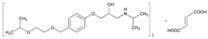	**Name: BISOPROLOL** **IUPAC Name:** 2-Propanol, 1-[4-[[2-(1-methylethoxy)ethoxy]methyl]phenoxy]-3-[(1-methylethyl)amino]-, (±)-, I-2-butenedioate (2:1); (2)(±)-1-[[α-(2-Isopropoxyethoxy)-p-tolyl]oxy]-3-(isopropylamino)-2-propanol fumarate (2:1) **Molecular Formula:** (C_18_H_31_NO_4_)_2_ C_4_H_4_O_4_ **Molecular Weight:** 766.96 **Mechanism:** Selective inhibitor of β1-adrenergic receptors (competitively blocks β1 receptors in myocardium), with little or no effect on β2 receptors at doses ≤20 mg (at high doses may block β2-adrenergic receptors within the bronchial and vascular smooth muscle). Decreases resting and exercise-stimulated heart rate and cardiac output, decreases isoproterenol-induced tachycardia, prolongs sinus node recovery time, refractory period of the AV node, and AV nodal conduction (with rapid atrial stimulation). No intrinsic sympathomimetic activity or membrane-stabilizing effect on the heart. Reduces blood pressure by decreasing cardiac output, decreasing sympathetic outflow from the CNS, and/or suppressing renin release. **Effect:** Treatment of hypertension, alone or in combination with other agents. Management of mild to moderately severe heart failure of ischemic or cardiomyopathic origin in conjunction with other agents (do not use in patients with acutely decompensated heart failure requiring I.V. inotropic therapy, those with substantial fluid retention requiring intensive diuresis, and those who require hospitalization for heart failure).	**Mechanistic genes:***ACE1*, *ADRB1*, *AGT*, *BDKRB2*, *GNAS* **Metabolic genes** **Substrate:***CYP2D6*, *CYP3A4*, *CYP3A5*
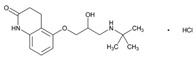	**Name: CARTEOLOL** **IUPAC Name:** 2(1H)-quinolinone, 5-[3-[(1,1-dimethylethyl)amino]-2-hydroxypropoxy]-3,4-dihydro-, monohydrochloride; 5-[3-(tert-Butylamino)-2-hydroxypropoxy]-3,4-dihydrocarbostyril monohydrochloride **Molecular Formula:** C_16_H_24_N_2_O_3_ HCl **Molecular Weight:** 328.83 **Mechanism:** Blocks both β1 and β2 receptors and has mild intrinsic sympathomimetic activity. Reduces intraocular pressure by decreasing aqueous humor production. Has negative inotropic and chronotropic effects and can significantly slow AV nodal conduction. **Effect:** Chronic open-angle glaucoma and intraocular hypertension.	**Mechanistic genes:***ADRB1*, *ADRB2* **Metabolic genes** **Substrate:***CYP2D6*, *CYP3A4*, *CYP3A5*
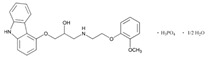	**Name: CARVEDILOL** **IUPAC Name:** 2-Propanol, 1-(9H-carbazol-4-yloxy)-3-[[2-(2-methoxyphenoxy)ethyl]amino]-, phosphate (salt), hydrate (2:2:1) **Molecular Formula:** C_24_H_26_N_2_O_4_ H_3_O_4_P ½H_2_O **Molecular Weight:** 513.48 **Mechanism:** Non-selective β-adrenergic blocker with α-adrenergic blocking activity. Available as a racemic mixture. Does not possess intrinsic sympathomimetic activity. Has calcium channel blocking activity at higher dose (30-fold the normal dose). In hypertensive patients, reduces cardiac output, exercise- or β-agonist-induced tachycardia, reflex orthostatic tachycardia, vasodilatation, peripheral vascular resistance (especially in standing position), renal vascular resistance, plasma renin activity, and increases levels of atrial natriuretic peptide. In congestive heart failure, decreases systemic blood pressure, pulmonary capillary wedge pressure, pulmonary artery pressure, heart rate, systemic vascular resistance, right arterial pressure, and increases stroke volume index and left ventricular ejection fraction. **Effect:** Mild-to-severe heart failure of ischemic or cardiomyopathic origin (usually in addition to standard therapy). Left ventricular dysfunction following myocardial infarction (clinically stable with LVEF ≤40%). Management of hypertension.	**Mechanistic genes:***ADRA1A*, *ADRB1*, *ADRB2*, *GRK5*, *MMP2*, *NOX1* **Metabolic genes** **Substrate:***CYP1A2*, *CYP2C9*, *CYP2C19*, *CYP2D6*, *CYP2E1*, *CYP3A4*, *CYP3A5*, *UGT1A1*, *UGT1A4*, *UGT1A6*, *UGT2B7* **Inhibitor:***ABCB1* **Transporter genes:***ABCB1*
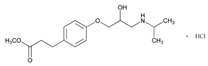	**Name: ESMOLOL** **IUPAC Name:** Benzenepropanoic acid, 4-[2-hydroxy-3-[(1-methylethyl)amino]propoxy]-, methyl ester, hydrochloride, (±)-; (±)-methyl p-[2-hydroxy-3-(isopropylamino)propoxy]hydrocinnamate hydrochloride **Molecular Formula:** C_16_H_25_NO_4_ HCl **Molecular Weight:** 331.83 **Mechanism:** A short-acting β1-selective adrenergic blocking agent. Competitively blocks response to β1-adrenergic stimulation with little or no effect on β2 receptors except at high doses, no intrinsic sympathomimetic activity, no membrane-stabilizing activity. **Effect:** Used in management of supraventricular tachyarrhythmias (e.g., atrial flutter and/or fibrillation, and sinus tachycardia). Used to prevent or treat increases in blood pressure associated with surgical events, including hypertensive crises (i.e., emergencies and urgencies). Treatment of non-compensatory sinus tachycardia.	**Mechanistic genes:** *ADRB1* **Metabolic genes** **Substrate:** *CYP2D6*
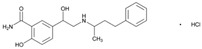	**Name: LABETALOL** **IUPAC Name:** Benzamide, 2-hydroxy-5-[1-hydroxy-2-[(1-methyl-3-phenylpropyl)amino]ethyl]-, monohydrochloride; 5-[1-hydroxy-2-[(1-methyl-3-phenylpropyl)amino]ethyl]salicylamide monohydrochloride **Molecular Formula:** C_19_H_24_N_2_O_3_ HCl **Molecular Weight:** 364.87 **Mechanism:** Blocks α-, β1-, and β2-adrenergic receptor sites. Elevated renins reduced. **Effect:** Treatment of mild to severe hypertension. Used intravenously for hypertensive emergencies.	**Mechanistic genes:***ADRA*, *ADRB1*, *ADRB2* **Metabolic genes** **Substrate:***UGT1A1*, *UGT2B7*
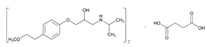	**Name: METOPROLOL** **IUPAC Name:** 2-Propanol, 1-[4-(2-methoxyethyl)phenoxy]-3-[(1-methylethyl)amino]-, (±)-, butanedioate (2:1); (±)-1-(isopropylamino)-3-[p-(2-methoxyethyl)phenoxy]-2-propanol succinate (2:1) **Molecular Formula:** (C_15_H_25_NO_3_)_2_ C_4_H_6_O_4_ **Molecular Weight:** 652.82 **Mechanism:** Competitively blocks β1 receptors, with little or no effect on β2 receptors at doses <100 mg. **Effect:** Treatment of angina pectoris, hypertension, or hemodynamically stable acute myocardial infarction. Treatment of angina pectoris or hypertension. To reduce mortality/hospitalization in heart failure patients already receiving ACEIs, diuretics, and/or digoxin.	**Mechanistic genes:***ADRB1*, *ADRA2C*, *ACE*, *GRK5*, *KCNH2* **Metabolic genes** **Substrate:***CYP2C19*, *CYP2D6* **Inhibitor:***CYP2D6* **Transporter genes:***ABCB1*
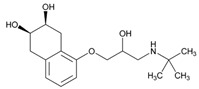	**Name: NADOLOL** **IUPAC Name:** 2,3-Naphthalenediol, 5-[3-[(1,1-dimethylethyl)amino]-2-hydroxypropoxy]-1,2,3,4-tetrahydro-, cis-; 1-(tert-butylamino)-3-[(5,6,7,8-tetrahydro-cis-6,7-dihydroxy-1-naphthyl)oxy]-2-propanol **Molecular Formula:** C_17_H_27_NO_4_ **Molecular Weight:** 309.40 **Mechanism:** Competitively blocks response to β1- and β2-adrenergic stimulation. **Effect:** Treatment of hypertension and angina pectoris. Prophylaxis of migraine headaches.	**Mechanistic genes:***ADRB1*, *ADRB2*, *ADRB3* **Transporter genes:***ABCB1* **Pleiotropic genes:***IL10*, *IL12B*
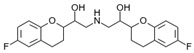	**Name: NEBIVOLOL** **IUPAC Name:** 2-H-1-benzopyran-2-methanol, α,α’-[iminobis(methylene)bis[6-fluoro-3,4-dihydro-; α,α’-(iminodimethylene)bis[6-fluoro-2-chromanmethanol] **Molecular Formula:** C_22_H_25_F_2_NO_4_ **Molecular Weight:** 405.44 **Mechanism:** Highly-selective inhibitor of β1-adrenergic receptors. Nebivolol, unlike other β-blockers, also produces an endothelium-derived nitric oxide-dependent vasodilation resulting in a reduction in systemic vascular resistance. **Effect:** Treatment of hypertension, alone or in combination with other agents.	**Mechanistic genes:***ADRB1* **Metabolic genes** **Substrate:***CYP2D6*, *UGTs* **Inducer:***NOS3*
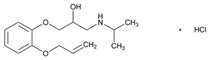	**Name: OXPRENOLOL** **IUPAC Name:** 2-Propanol, 1-(o-allyloxyphenoxy)-3-isopropylamino-, hydrochloride **Molecular Formula:** C_15_H_23_NO_3_ HCl **Molecular Weight:** 301.81 **Mechanism:** A competitive and non-selective antagonist of β-adrenergic receptors. Antagonizes catecholamine-induced tachycardia, thus decreasing cardiac output. Inhibits renin release by kidneys and inhibits vasomotor centers. **Effect:** Mild or moderate hypertension.	**Mechanistic genes:***ADRB1*, *ADRB2*, *ADRB3* **Metabolic genes** **Inhibitor:***CYP2D6*
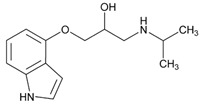	**Name: PINDOLOL** **IUPAC Name:** 2-Propanol, 1-(1H-indol-4-yloxy)-3-(1-methylethyl)amino-; 1-(indol-4-yloxy)-3-(isopropylamino)-2-propanol **Molecular Formula:** C_14_H_20_N_2_O_2_ **Molecular Weight:** 248.32 **Mechanism:** Blocks both β1 and β2 receptors and has mild intrinsic sympathomimetic activity. Has negative inotropic and chronotropic effects and can significantly slow AV nodal conduction. Augmentative action of antidepressants thought to be mediated via serotonin 1A autoreceptor antagonism. **Effect:** Treatment of hypertension, alone or in combination with other agents.	**Mechanistic genes:***ADRB1*, *ADRB2*, *ADRB3*, *GRK5*, *HTR1A*, *HTR1B* **Metabolic genes** **Substrate:***CYP2D6* **Inhibitor:***CYP2D6*
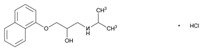	**Name: PROPRANOLOL** **IUPAC Name:** 2-Propanol, 1-[(1-methylethyl)amino]-3-(1-naphthalenyloxy)-, hydrochloride, (±)-; (±)-1-(isopropylamino)-3-(1-naphthyloxy)-2-propanol hydrochloride **Molecular Formula:** C_16_H_21_NO_2_ HCl **Molecular Weight:** 295.80 **Mechanism:** Competitively blocks response to β1- and β2-adrenergic stimulation, which results in decreases in heart rate, myocardial contractility, blood pressure, and myocardial oxygen demand. Reduces portal pressure by producing splanchnic vasoconstriction (β2-effect) thereby reducing portal blood flow. **Effect:** Management of hypertension. Angina pectoris. Pheochromocytoma. Essential tremor. Supraventricular arrhythmias (such as atrial fibrillation and flutter, AV nodal re-entrant tachycardias), ventricular tachycardias (catecholamine-induced arrhythmias, digoxin toxicity). Prevention of myocardial infarction. Migraine headache prophylaxis. Symptomatic treatment of hypertrophic subaortic stenosis (hypertrophic obstructive cardiomyopathy).	**Mechanistic genes:***ADRB1*, *ADRB2*, *ADRB3*, *ALOX5*, *CFTR*, *COMT*, *FOS*, *GNAS*, *GRK5*, *HTR1B*, *HTR3B*, *KCNE2; KCNH2*, *KCNQ1*, *NPY*, *PPARGC1A*, *PTGS2* **Metabolic genes** **Substrate:***CYP1A1*, *CYP1A2*, *CYP2C9*, *CYP2C19*, *CYP2D6*, *CYP3A4*, *CYP3A5*, *UGT2B7* **Inhibitor:***ABCB1*, *CYP1A2*, *CYP2D6* **Transporter genes:***ABCB1* **Pleiotropic genes:***IL1B*, *TNF*
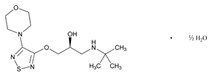	**Name: TIMOLOL** **IUPAC Name:** 2-Propanol, 1-[(1,1-dimethylethyl)amino]-3-[[4-(4-morpholinyl)-1,2,5-thiadiazol-3-yl]oxy]-, hemihydrate, (S)-; (S)-1-(tert-butylamino)-3-[(4-morpholino-1,2,5-thiadiazol-3-yl)oxy]-2-propanol hemihydrate **Molecular Formula:** C_13_H_24_N_4_O_3_S ½H_2_O **Molecular Weight:** 325.43 **Mechanism:** Blocks both β1- and β2-adrenergic receptors, reduces intraocular pressure by reducing aqueous humor production or possibly outflow. Reduces blood pressure by blocking adrenergic receptors and decreasing sympathetic outflow, produces negative chronotropic and inotropic activity through unknown mechanism. **Effect:** Treatment of elevated intraocular pressure such as glaucoma or ocular hypertension. Treatment of hypertension and angina, to reduce mortality following myocardial infarction. Prophylaxis of migraine.	**Mechanistic genes:***ADRB1*, *GNAS* **Metabolic genes** **Substrate:***CYP2C19*, *CYP2D6* **Inhibitor:***CYP2D6*
**Calcium-Channel Blockers**		
**Drug**	**Properties**	**Pharmacogenetics**
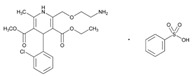	Name: AMLODIPINE **IUPAC Name:** 3,5-Pyridinedicarboxylic acid, 2-[(2-aminoethoxy)methyl]-4-(2-chlorophenyl)-1,4-dihydro-6-methyl-, 3-ethyl 5-methyl ester, (±)-, monobenzenesulfonate; 3-ethyl 5-methyl (±)-2-[(2-aminoethoxy)methyl]-4-(o-chlorophenyl)-1,4-dihydro-6-methyl-3,5-pyridinedicarboxylate, monobenzenesulfonate **Molecular Formula:** C_20_H_25_ClN_2_O_5_ C_6_H_6_O_3_S **Molecular Weight:** 567.05 **Mechanism:** Inhibits calcium ion from entering the voltage-sensitive channels of vascular smooth muscle and myocardium during depolarization, producing a relaxation of coronary vascular smooth muscle and coronary vasodilation. Increases myocardial oxygen delivery in vasospastic angina. **Effect:** Treatment of hypertension, symptomatic chronic stable angina, vasospastic (Prinzmetal’s) angina. Prevention of hospitalization due to angina with documented coronary artery disease.	**Mechanistic genes:***ADD1*, *AGT*, *CACNs*, *NPPA* **Metabolic genes** **Substrate:***CYP3A4*, *CYP3A5* **Inhibitor:***ABCB1*, *CYP1A1*, *CYP1A2*, *CYP2A6*, *CYP2B6*, *CYP2C8*, *CYP2C9*, *CYP2D6*, *CYP3A4*, *CYP3A5* **Transporter genes:***ABCB1*
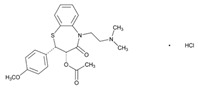	**Name: DILTIAZEM** **IUPAC Name:** 1,5-Benzothiazepin-4(5H)-one, 3-(acetyloxy)-5-[2-(dimethylamino)ethyl]-2,3-dihydro-2-(4-methoxyphenyl)-, monohydrochloride, (+)-cis-; (+)-5-[2-(dimethylamino)ethyl]-cis-2,3-dihydro-3-hydroxy-2-(p-methoxyphenyl)-1,5-benzothiazepin-4(5H)-one acetate (ester) monohydrochloride **Molecular Formula:** C_22_H_26_N_2_O_4_S HCl **Molecular Weight:** 450.98 **Mechanism:** Inhibits calcium ions from entering the “slow channels” or select voltage-sensitive areas of vascular smooth muscle and myocardium during depolarization, producing a relaxation of coronary vascular smooth muscle and coronary vasodilation. Increases myocardial oxygen delivery in vasospastic angina. **Effect:** Essential hypertension, chronic stable angina or angina from coronary artery spasm. Temporary control of rapid ventricular rate in atrial fibrillation or flutter. Management of supraventricular tachyarrhythmias, including rapid conversion to sinus rhythm of paroxysmal supraventricular tachycardias (e.g., those associated with Wolff–Parkinson–White or Lown–Ganong–Levine syndrome).	**Mechanistic genes:***CACNA* **Metabolic genes** **Substrate:***CYB5s*, *CYP2C8*, *CYP2C9*, *CYP2D6*, *CYP3A4*, *CYP3A5* **Inhibitor:***ABCB1*, *CYB5s*, *CYP2C9*, *CYP2D6*, *CYP3A4*, *CYP3A5* **Transporter genes:***ABCB1*, *SLCO1B1* **Pleiotropic genes:***IL12B*
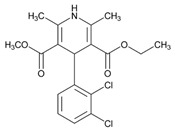	**Name: FELODIPINE** **IUPAC Name:** 3,5-Pyridinedicarboxylic acid 4-(2,3-dichlorophenyl)-1,4-dihydro-2,6-dimethyl-, ethyl methyl ester, (±)-; (±)-ethyl methyl 4-(2,3-dichlorophenyl)-1,4-dihydro-2,6-dimethyl-3,5-pyridinedicarboxylate **Molecular Formula:** C_18_H_19_Cl_2_NO_4_ **Molecular Weight:** 384.25 **Mechanism:** Inhibits calcium ions from entering the “slow channels” or select voltage-sensitive areas of vascular smooth muscle and myocardium during depolarization, producing a relaxation of coronary vascular smooth muscle and coronary vasodilation. Increases myocardial oxygen delivery in vasospastic angina. **Effect:** Treatment of hypertension.	**Mechanistic genes:***CACNA1C*, *NR1I2* **Metabolic genes** **Substrate:***CYP3A4*, *CYP3A5* **Inhibitor:***ABCB1*, *CYP2C8*, *CYP2C9*, *CYP2D6*, *CYP3A4*, *CYP3A5*
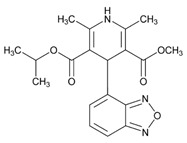	**Name: ISRADIPINE** **IUPAC Name:** 3,5-Pyridinedicarboxylic acid, 4-(4-benzofurazanyl)-1,4-dihydro-2,6-dimethyl-, methyl 1-methylethyl ester, (±)-; isopropyl methyl (±)-4-(4-benzofurazanyl)-1,4-dihydro-2,6-dimethyl-3,5-pyridinedicarboxylate **Molecular Formula:** C_19_H_21_N_3_O_5_ **Molecular Weight:** 371.39 **Mechanism:** Inhibits transmembrane influx of extracellular calcium ions across membranes of myocardial cells and vascular smooth muscle cells, without changing serum calcium concentrations. Increases myocardial oxygen delivery in vasospastic angina. **Effect:** Management of hypertension (alone or in combination with other classes of antihypertensive agents).	**Mechanistic genes:***CACNA1C*, *NR1I2* **Metabolic genes** **Substrate:***CYP3A4*, *CYP3A5* **Inhibitor:***CYP3A4*, *CYP3A5*
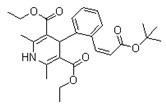	**Name: LACIDIPINE** **IUPAC Name:** 3,5-Pyridinedicarboxylic acid, 4-[2-[3-(1,1-dimethylethoxy)-3-oxo-1-propenyl]phenyl]-1,4-dihydro-2,6-dimethyl-, diethyl ester, I-; 4-I(E)-2-carboxyvinyl]-phenyl]-1,4-dihydro-2,6-dimethyl-3,5-pyridinedicarboxylic acid, 4-tert-butyl diethyl ester **Molecular Formula:** C_26_H_33_NO_6_ **Molecular Weight:** 455.54 **Mechanism:** A specific and potent calcium antagonist with predominant selectivity for calcium channels in vascular smooth muscle. Its main action is to dilate peripheral arterioles, reducing peripheral vascular resistance and lowering blood pressure. **Effect:** Indicated for treatment of hypertension either alone or in combination with other antihypertensive agents, including β-adrenoceptor antagonists, diuretics, and ACEIs.	**Mechanistic genes:***CACN* **Metabolic genes** **Substrate:** CYP3A4, CYP35 **Inhibitor:** CYP3A4, CYP35
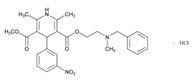	**Name: NICARDIPINE** **IUPAC Name:** 3,5-Pyridinedicarboxylic acid, 1,4-dihydro-2,6-dimethyl-4-(3-nitrophenyl)-, methyl 2-[methyl(phenylmethyl)amino]ethyl ester, monohydrochloride; 2-(benzylmethylamino)ethyl methyl 1,4-dihydro-2,6-dimethyl-4-(m-nitrophenyl)-3,5-pyridinedicarboxylate monohydrochloride **Molecular Formula:** C_26_H_29_N_3_O_6_ HCl **Molecular Weight:** 515.99 **Mechanism:** Inhibits calcium ions from entering “slow channels” or select voltage-sensitive areas of vascular smooth muscle and myocardium during depolarization, producing relaxation of coronary vascular smooth muscle and coronary vasodilation. Increases myocardial oxygen delivery in vasospastic angina. **Effect:** Chronic stable angina. Management of hypertension.	**Mechanistic genes:***CACNA1C*, *CACNA1D*, *CACNA2D1*, *CACNB2* **Metabolic genes** **Substrate:***CYP1A1*, *CYP1A2*, *CYP2B6*, *CYP2C8*, *CYP2C9*, *CYP2D6*, *CYP2E1*, *CYP3A4*, *CYP3A5* **Inhibitor:***ABCB1*, *CYP1A1*, *CYP2A6*, *CYP2C8*, *CYP2C9*, *CYP2C19*, *CYP2D6*, *CYP3A4*, *CYP3A5* **Inducer:***CYP1A2* **Transporter genes:***ABCB1*
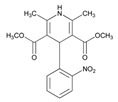	**Name: NIFEDIPINE** **IUPAC Name:** 3,5-Pyridinedicarboxylic acid, 1,4-dihydro-2,6-dimethyl-4-(2-nitrophenyl)-, dimethyl ester; dimethyl 1,4-dihydro-2,6-dimethyl-4-(o-nitrophenyl)-3,5-pyridinedicarboxylate **Molecular Formula:** C_17_H_18_N_2_O_6_ **Molecular Weight:** 346.33 **Mechanism:** Inhibits calcium ions from entering “slow channels” or select voltage-sensitive areas of vascular smooth muscle and myocardium during depolarization, producing relaxation of coronary vascular smooth muscle and coronary vasodilation. Increases myocardial oxygen delivery in vasospastic angina. **Effect:** Angina and hypertension. Pulmonary hypertension.	**Mechanistic genes:***ACE1*, *ACE2*, *CACNA1C*, *DRD2*, *FOS*, *MMP2*, *SCN5A* **Metabolic genes** **Substrate:***CYP2D6*, *CYP2C8*, *CYP11B2*, *CYP3A4*, *CYP3A5*, *POR* **Inhibitor:***ABCB1*, *CYP1A2*, *CYP2C9*, *CYP2D6*, *CYP2E1*, *CYP3A4*, *CYP3A5* **Transporter genes:***ABCB1*, *ABCC2*, *ABCC3*, *SLC14A2* **Pleiotropic genes:***TP53*
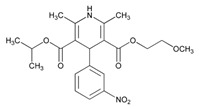	**Name: NIMODIPINE** **IUPAC Name:** 3,5-Pyridinedicarboxylic acid, 1,4-dihydro-2,6-dimethyl-4-(3-nitrophenyl)-, 2-methoxyethyl 1-methylethyl ester; isopropyl 2-methoxyethyl 1,4-dihydro-2,6-dimethyl-4-(m-nitrophenyl)-3,5-pyridinedicarboxylate **Molecular Formula:** C_21_H_26_N_2_O_7_ **Molecular Weight:** 418.44 **Mechanism:** Animal studies indicate that nimodipine has greater effect on cerebral arterials than other arterials; this increased specificity may be due to increased lipophilicity and cerebral distribution of the drug as compared to nifedipine. Inhibits calcium ions from entering “slow channels” or select voltage-sensitive areas of vascular smooth muscle and myocardium during depolarization. **Effect:** Spasm following subarachnoid hemorrhage from ruptured intracranial aneurysms regardless of patient’s postictus neurological condition.	**Mechanistic genes:***CACNA1C*, *DRD2* **Metabolic genes** **Substrate:***CYP3A4*, *CYP35* **Pleiotropic genes:***APP*
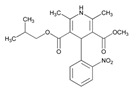	**Name: NISOLDIPINE** **IUPAC Name:** 3,5-Pyridinedicarboxylic acid, 1,4-dihydro-2,6-dimethyl-4-(2-nitrophenyl)-, methyl 2-methylpropyl ester, (±)-; (±)-isobutyl methyl 1,4-dihydro-2,6-dimethyl-4-(o-nitrophenyl)-3,5-pyridinedicarboxylate **Molecular Formula:** C_20_H_24_N_2_O_6_ **Molecular Weight:** 388.41 **Mechanism:** As a dihydropyridine calcium-channel blocker, structurally similar to nifedipine, nisoldipine impedes movement of calcium ions into vascular smooth muscle and cardiac muscle. Dihydropyridines are potent vasodilators and not as likely to suppress cardiac contractility and slow cardiac conduction as other calcium antagonists such as verapamil and diltiazem. Nisoldipine is 5–10-fold as potent vasodilator as nifedipine. **Effect:** Management of hypertension, alone or in combination with other antihypertensive agents.	**Mechanistic genes:***CACNA1C* **Metabolic genes** **Substrate:***CYP34*, *CYP3A5* **Inhibitor:***ABCB1*, *CYP1A2*, *CYP3A4*, *CYP3A5*
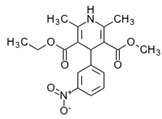	**Name: NITRENDIPINE** **IUPAC Name:** 3,5-Pyridinedicarboxylic acid, 1,4-dihydro-2,6-dimethyl-4-(3-nitrophenyl)-, ethyl methyl ester, (±)-; (±)-ethyl methyl 1,4dihydro-2,6-dimethyl-4-(m-nitrophenyl)-3,5-pyridinedicarboxylate **Molecular Formula:** C_18_H_20_N_2_O_6_ **Molecular Weight:** 360.36 **Mechanism:** Dihydropyridine calcium channel-blocking agent with actions similar to nifedipine. **Effect:** Management of hypertension.	**Mechanistic genes:***CACNA1C*, *CACNG1* **Metabolic genes** **Substrate:***CYP3A4*, *CYP3A5* **Inhibitor:***ABCB1*, *CYP3A4*, *CYP3A5* **Transporter genes:***ABCG2*
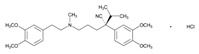	**Name: VERAPAMIL** **IUPAC Name:** Benzeneacetonitrile, α-[3-[[2-(3,4-dimethoxyphenyl)ethyl]methylamino]propyl]-3,4-dimethoxy-α-(1-methylethyl)-, monohydrochloride, (±)-; (±)-5-[(3,4-dimethoxyphenethyl)methylamino]-2-(3,4-dimethoxyphenyl)-2-isopropylvaleronitrile monohydrochloride **Molecular Formula:** C_27_H_38_N_2_O_4_ HCl **Molecular Weight:** 491.06 **Mechanism:** Inhibits calcium ions from entering “slow channels” or select voltage-sensitive areas of vascular smooth muscle and myocardium during depolarization. Produces relaxation of coronary vascular smooth muscle and coronary vasodilation. Increases myocardial oxygen delivery in vasospastic angina. Slows automaticity and conduction of the AV node. **Effect:** Orally for treatment of angina pectoris (vasospastic, chronic stable, and unstable) and hypertension. I.V. for supraventricular tachyarrhythmias (PSVT, atrial fibrillation, and atrial flutter).	**Mechanistic genes:***ADRB1*, *ADRB2*, *CACNA1C*, *CACNs*, *CFTR*, *KCNMB1*, *LDLR*, *NOS1AP*, *RET*, *TGFB1* **Metabolic genes** **Substrate:***CYP1A2*, *CYP2B6*, *CYP2C8*, *CYP2C9*, *CYP2C18*, *CYP2C19*, *CYP2E1*, *CYP2J2*, *CYP3A4*, *CYP3A5*, *CYP3A7*, *SOD2* **Inhibitor:***ABCB1*, *ABCC1*, *ABCC2*, *CYP1A2*, *CYP2C8*, *CYP2C9*, *CYP2D6*, *CYP3A4*, *CYP3A5*, *CYP3A7* **Inducer:***ABCB1* **Transporter genes:***ABCB1*, *ABCC3*, *SLCO1B1* **Pleiotropic genes:***TNF*
**Cardiotonic Agents**		
**Drug**	**Properties**	**Pharmacogenetics**
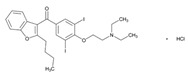	**Name: AMIODARONE** **IUPAC Name:** Methanone, (2-butyl-3-benzofuranyl)[4-[2-(diethylamino)ethoxy]-3,5-diiodophenyl]-; 2-butyl-3-benzofuranyl 4-[2-(diethylamino)ethoxy]-3,5-diiodophenyl ketone **Molecular Formula:** C_25_H_29_I_2_NO_3_ **Molecular Weight:** 645.31 **Mechanism:** Inhibits adrenergic stimulation (α- and β-blocking properties), affects sodium, potassium and calcium channels, and prolongs the action potential and refractory period in myocardial tissue. Decreases AV conduction and sinus node function. **Effect:** Management of recurrent ventricular fibrillation or hemodynamically-unstable ventricular tachycardia.	**Mechanistic genes:***ABL1*, *ACOX1*, *ADRA2A*, *ADRB1*, *ADRB2*, *CHRM2*, *FABP1*, *FMO1*, *FOS*, *ICAM1*, *KCNE1*, *KCNE2*, *KCNH2*, *KCNJ11*, *KCNQ1*, *PSEN1*, *SCN5A* **Metabolic genes** **Substrate:***CYP1A1*, *CYP1A2*, *CYP2C8*, *CYP2C9*, *CYP2C19*, *CYP2D6*, *CYP2J2*, *CYP3A4*, *CYP3A5* **Inhibitor:***ABCB1*, *CYP1A2*, *CYP2A6*, *CYP2B6*, *CYP2C8*, *CYP2C9*, *CYP2C19*, *CYP2D6*, *CYP3A4*, *CYP3A5* **Inducer:***CYP1A2* **Transporter genes:***ABCB1*, *ABCC6*, *ABCC8*, *SLC5A5*
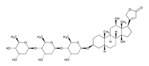	**Name: DIGOXIN** **IUPAC Name:** Card-20(22)-enolide, 3-[(O-2,6-dideoxy-β-D-ribo-hexopyranosyl-(1→4)-O-2,6-dideoxy-β-D-ribo-hexopyranosyl-(1→4)-2,6-dideoxy-β-D-ribo-hexopyranosyl)oxy]-12,14-dihydroxy-, (3β,5β,12β)-; 3β-[(O-2,6-dideoxy-β-D-ribo-hexopyranosyl-(1→4)-O-2,6-dideoxy-β-D-ribo-hexopyranosyl-(1→4)-2,6-dideoxy-β-D-ribo-hexopyranosyl)oxy]-12β,14-dihydroxy-5β-card-20(22)-enolide **Molecular Formula:** C_41_H_64_O_14_ **Molecular Weight:** 780.94 **Mechanism:** Digoxin is a cardiac glycoside with positive inotropic effects. In congestive heart failure it inhibits the sodium/potassium ATPase pump which acts to increase the intracellular sodium-calcium exchange to increase intracellular calcium, leading to increased contractility. In supraventricular arrhythmias: Direct suppression of the AV node conduction to increase effective refractory period and decrease conduction velocity; positive inotropic effect, enhanced vagal tone, and decreased ventricular rate to fast atrial arrhythmias. Atrial fibrillation may decrease sensitivity and increase tolerance to higher serum digoxin concentrations. **Effect:** Digitalization and maintenance therapy. Used principally in the prophylactic management and treatment of congestive heart failure and to control the ventricular rate in supraventricular tachyarrhythmias (e.g., atrial fibrillation or flutter). Used to improve left ventricular function in cardiogenic shock and atrial fibrillation or flutter with rapid ventricular rate. May be useful, especially in conjunction with a β-adrenergic blocking agent, in the treatment of angina pectoris when cardiomegaly and congestive heart failure are present.	**Mechanistic genes:***ATP1A1* **Metabolic genes** **Substrate:***CYP3A4*, *CYP3A5* **Transporter genes:***ABCB1*, *ABCB11*, *ABCG2*, *SLCO1B3*
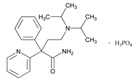	**Name: DISOPYRAMIDE** **IUPAC Name:** 2-Pyridineacetamide, α-[2-[bis(1-methylethyl)amino]ethyl]-α-phenyl-, (±)-, phosphate (1:1); (±)-α-[2-(diisopropylamino)ethyl]-α-phenyl-2-pyridineacetamide phosphate (1:1) **Molecular Formula:** C_21_H_29_N_3_O H_3_PO_4_ **Molecular Weight:** 437.47 **Mechanism:** Decreases myocardial excitability and conduction velocity. Reduces disparity in refractory period between normal and infarcted myocardium. Possesses anticholinergic, peripheral vasoconstrictive, and negative inotropic effects. **Effect:** Suppression and prevention of unifocal and multifocal ventricular premature complexes, coupled ventricular premature complexes and/or paroxysmal ventricular tachycardia in primary arrhythmias or arrhythmias secondary to coronary artery disease.	**Mechanistic genes:***ADRB1*, *ADRB2*, *CHRM2*, *KCNE1*, *KCNE2*, *KCNH2*, *KCNJ11*, *KCNQ1* **Metabolic genes** **Substrate:***CYP2D6*, *CYP3A4*, *CYP3A5* **Inhibitor:***CYP1A1*, *CYP1A2*, *CYP2C19*, *CYP3A4*, *CYP3A5* **Transporter genes:***ABCC8*
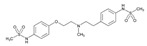	**Name: DOFETILIDE** **IUPAC Name:** Methanesulfonamide, N-[4-[2-[methyl[2-[4-[(methylsulfonyl)amino]phenoxy]ethyl]amino]ethyl]phenyl]-; β-[(p-methanesulfonamidophenethyl)methylamino]methanesulfono-p-phenetidide **Molecular Formula:** C_19_H_27_N_3_O_5_S_2_ **Molecular Weight:** 441.56 **Mechanism:** Class III antiarrhythmic agent. Blockade of the cardiac ion channel carrying the rapid component of the delayed rectifier potassium current. It increases the monophasic action potential duration due to delayed repolarization. The increase in the QT interval is a function of prolongation of both effective and functional refractory periods in the His–Purkinje system and the ventricles. **Effect:** Used for the maintenance of normal sinus rhythm in patients with atrial fibrillation/flutter of more than 1 week duration who have been converted to normal sinus rhythm. Additionally used for the conversion of atrial fibrillation and atrial flutter to normal sinus rhythm.	**Mechanistic genes:***ADRA2A*, *ADRB1*, *CHRM2*, *KCNE1*, *KCNE2*, *KCNH2*, *KCNJ11*, *KCNQ1* **Metabolic genes** **Substrate:***CYP2D6*, *CYP3A4*, *CYP3A5* **Transporter genes:***ABCC8*
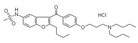	**Name: DRONEDARONE** **IUPAC Name:** N-[2-butyl-3-[4-[3-(dibutylamino)propoxy]benzoyl]-1-benzofuran-5-yl] methanesulfonamide, hydrochloride **Molecular Formula:** C_31_H_44_N_2_O_5_S HCl **Molecular Weight:** 593.22 **Mechanism:** Dronedarone has antiarrhythmic properties belonging to all four antiarrhythmic (Vaughan-Williams) classes, but the contribution of each of these activities to the clinical effect is unknown. Inhibits sodium (INa) and potassium (Ikr, IkS, Ik1, and Ik-ACh) channels resulting in prolongation of the action potential and refractory period in myocardial tissue without reverse-use-dependent effects. Decreases AV conduction and sinus node function through inhibition of calcium (ICa-L) channels and β1-receptor blocking activity. Similar to amiodarone, dronedarone also inhibits α1-receptor-mediated increases in blood pressure. **Effect:** Indicated to reduce the risk of hospitalization related to paroxysmal or persistent atrial fibrillation (AF) or atrial flutter (AFl) in patients with a recent episode of AF/AFl and associated cardiovascular risk factors (e.g., age >70 years, hypertension, diabetes, prior cerebrovascular accident, left atrial diameter ≥50 mm or left ventricular ejection fraction <40%), who are in normal sinus rhythm or will be cardioverted.	**Mechanistic genes:***KCNA5*, *KCNE2*, *KCNH2* **Metabolic genes** **Substrate:***CYP3A4*, *CYP3A5* **Inhibitor:***ABCB1*, *CYP2C9*, *CYP2D6*, *CYP3A4*, *CYP3A5*
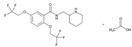	**Name: FLECAINIDE** **IUPAC Name:** Benzamide, N-(2-piperidinylmethyl)-2,5-bis(2,2,2-trifluoroethoxy)-, monoacetate **Molecular Formula:** C_17_H_20_F_6_N_2_O_3_ C_2_H_4_O_2_ **Molecular Weight:** 474.39 **Mechanism:** Flecainide is a local anesthetic-type class Ic antiarrhythmic agent. Slows conduction in cardiac tissue by altering transport of ions across cell membranes. Causes slight prolongation of refractory periods. Decreases rate of rise of action potential without affecting duration. Increases electrical stimulation threshold of ventricle, the His–Purkinje system. Possesses local anesthetic and moderate negative inotropic effects. **Effect:** Prevention and suppression of documented life-threatening ventricular arrhythmias (e.g., sustained ventricular tachycardia). Control of symptomatic, disabling supraventricular tachycardias in patients without structural heart disease where other agents fail.	**Mechanistic genes:***ADRA2A*, *CHRM2*, *FABP1*, *KCNE1*, *KCNE2*, *KCNH2*, *KCNJ11*, *KCNQ1*, *SCN5A* **Metabolic genes** **Substrate:***CYP1A2*, *CYP2D6* **Inhibitor:***CYP2D6* **Transporter genes:***ABCC8*
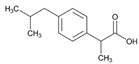	**Name: IBUPROFEN** **IUPAC Name:** Benzeneacetic acid, α-methyl-4-(2-methylpropyl), (±)-; (±)-p-isobutylhydratropic acid; (3)(±)-2-(p-isobutylphenyl)propionic acid **Molecular Formula:** C_13_H_18_O_2_ **Molecular Weight:** 206.28 **Mechanism:** Inhibits prostaglandin synthesis by decreasing activity of enzyme cyclooxygenase (COX/PTGS), which results in decreased formation of prostaglandin precursors. **Effect:** Inflammatory diseases and rheumatoid disorders including juvenile rheumatoid arthritis, mild-to-moderate pain, fever, dysmenorrhea. Ibuprofen lysine is for use in premature infants weighing between 500 and 1500 g and who are ≤32 weeks gestational age to induce closure of clinically significant patent ductus arteriosus (PDA) when usual treatments are ineffective. Management of pain and swelling.	**Mechanistic genes:***ACSL1*, *AGT*, *BCAR1*, *CCND1*, *CNR1*, *ERBB4*, *FOS*, *ICAM1*, *IFNG*, *IGF1*, *LTA*, *MMP3*, *NOS3*, *NQO1*, *NR3C1*, *PPARA*, *PPARG*, *PTGER3*, *PTGES*, *PTGIS*, *PTGS1*, *PTGS2*, *RB1*, *REN*, *SCARB1*, *SNCA*, *TBX21*, *TBXAS1*, *VCAM1*, *VEGFA* **Metabolic genes** **Substrate:***CYP1A2*, *CYP2B6*, *CYP2C8*, *CYP2C9*, *CYP2C19*, *CYP2D6*, *CYP2E1*, *CYP3A4*, *CYP3A5*, *CYP19A1*, *GSTT1*, *SOD2*, *UGT1A1*, *UGT1A3*, *UGT1A7*, *UGT1A9*, *UGT1A10*, *UGT2B7* **Inhibitor:***ACACA*, *CYP2C9*, *DDC*, *PTGS2*, *SLC5A8*, *SULT1A1*, *UGT2B15* **Inducer:***CYP19A1* **Transporter genes:***SLC15A1*, *SLC22A1*, *SLC22A6*, *SLC22A7*, *SLC22A8* **Pleiotropic genes:***APP*, *IL1B*, *IL1RN*, *IL4*, *IL6*, *IL10*, *TNF*
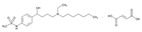	**Name: IBUTILIDE** **IUPAC Name:** Methanesulfonamide, N-[4-[4-(ethylheptylamino)-1-hydroxybutyl]phenyl]-, (±)-, (E)-2-butenedioate (2:1); (±)-4′-[4-(ethylheptylamino)-1-hydroxybutyl]methanesulfonanilide fumarate (2:1) **Molecular Formula:** (C_20_H_36_N_2_O_3_S)_2_ C_4_H_4_O_4_ **Molecular Weight:** 885.23 **Mechanism:** Prolongs action potential duration and effective refractory period in both atrial and ventricular cardiac tissue. Delays repolarization by activating a slow, predominantly sodium, inward current. Produces dose-related prolongation of QT interval, thought to be associated with antiarrhythmic activity. Negligible effects on heart rate, cardiac contractility, or blood pressure. Lacks β-adrenergic-blocking activity. **Effect:** Used for rapid conversion of recent-onset atrial flutter or fibrillation to sinus rhythm.	**Mechanistic genes:***ADRA2A*, *CACNA1C*, *CHRM2*, *KCNE1*, *KCNE2*, *KCNH2*, *KCNJ11*, *KCNQ1* **Metabolic genes** **Substrate:***CYP2D6* **Transporter genes:***ABCB1*, *ABCC8*
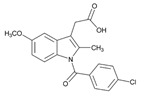	**Name: INDOMETHACIN** **IUPAC Name:** 1H-indole-3-acetic acid, 1-(4-chlorobenzoyl)-5-methoxy-2-methyl-; 1-(p-chlorobenzoyl)-5-methoxy-2-methylindole-3-acetic acid **Molecular Formula:** C_19_H_16_ClNO_4_ **Molecular Weight:** 357.79 **Mechanism:** Inhibits cyclooxygenase-1 (COX-1/PTGS1) and COX-2/PTGS2. Exhibits anti-inflammatory, analgesic, and antipyretic activity. Permits closure of ductus arteriosus in premature neonates by inhibiting prostaglandin synthesis. **Effect:** Symptomatic treatment of osteoarthritis, rheumatoid arthritis, and ankylosing spondylitis. Symptomatic relief of acute gout and acute painful shoulder (i.e., bursitis and/or tendinitis). Treatment of patent ductus arteriosus in premature neonates.	**Mechanistic genes:***AGTR1*, *BCAR1*, *CAT*, *CBR1*, *CBS*, *CCND1*, *CDK2*, *CDK4*, *COL1A1*, *DIO2*, *EDN1*, *EGFR*, *FOS*, *G6PD*, *GNAS*, *ICAM1*, *IFNG*, *LEP*, *LPL*, *LTA*, *MAPT*, *MMP2*, *MMP3*, *NOS3*, *NPPA*, *NPY*, *PARK2*, *PDGFRA*, *PDGFRB*, *PPARA*, *PPARD*, *PPARG*, *PTGER2*, *PTGES*, *PTGIS*, *PTGS1*, *PTGS2*, *RB1*, *TBXAS1*, *TGFB1*, *TGFBR1*, *THBD*, *TLR*, *UCP2*, *VEGFA*, *XDH* **Metabolic genes** **Substrate:***CYP1A1*, *CYP1A2*, *CYP1B1*, *CYP2B6*, *CYP2C8*, *CYP2C9*, *CYP2C19*, *CYP2D6*, *CYP2E1*, *CYP3A4*, *CYP3A5*, *CYP7A1*, *CYP19A1*, *CYP27A1*, *GSTM1*, *GSTT1*, *SOD2*, *UGT1A1*, *UGT1A3*, *UGT1A4*, *UGT1A7*, *UGT1A9*, *UGT1A10*, *UGT2B7* **Inhibitor:** *CYP2C9*, *CYP2C19*, *PTGS1*, *PTGS2*, *SLC22A7*, *SULT1A1* **Transporter genes:***ABCB1*, *ABCC1*, *ABCC2*, *ABCC3*, *ABCC6*, *ABCG2*, *SLC10A1*, *SLC19A1*, *SLC22A1*, *SLC22A2*, *SLC22A6*, *SLC22A7*, *SLC22A8* **Pleiotropic genes:***APC*, *APP*, *IL1B*, *IL1RN*, *IL2*, *IL4*, *IL6*, *IL10*, *IL12B*, *TNF*, *TNFRSF1A*, *TP53*
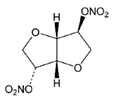	**Name: ISOSORBIDE DINITRATE** **IUPAC Name:** D-glucitol, 1,4:3,6-dianhydro-, dinitrate **Molecular Formula:** C_6_H_8_N_2_O_8_ **Molecular Weight:** 236.14 **Mechanism:** Stimulation of intracellular cyclic GMP results in vascular smooth muscle relaxation of both arterial and venous vasculature. **Effect:** Acute relief of angina pectoris, prophylactic management in situations likely to provoke angina attacks, and long-term prophylactic management of angina pectoris. Additionally used to relieve pain, dysphagia, and spasm in esophageal spasm with gastroesophageal reflux.	**Mechanistic genes:***ALDH3A1*, *EDN1*, *NOS3* **Metabolic genes** **Substrate:***CYP3A4*, *CYP3A5*, *CYP11B2*
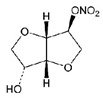	**Name: ISOSORBIDE MONONITRATE** **IUPAC Name:** D-glucitol, 1,4:3,6-dianhydro-, 5-nitrate; 1,4:3,6-dianhydro-D-glucitol 5-nitrate **Molecular Formula:** C_6_H_9_NO_6_ **Molecular Weight:** 191.14 **Mechanism:** Decreases preload as measured by pulmonary capillary wedge pressure and left ventricular end diastolic volume and pressure. This effect improves congestive symptoms in heart failure and improves myocardial perfusion gradient in coronary artery disease. **Effect:** Long-acting metabolite of vasodilator isosorbide dinitrate used for prophylactic treatment of angina pectoris.	**Mechanistic genes:***NOS3* **Metabolic genes** **Substrate:***CYP3A4*, *CYP3A5*, *CYP11B2*
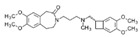	**Name: IVABRADINE** **IUPAC Name:** 3-[3-[[[(7S)-3,4-dimethoxybicyclo[4.2.0]octa-1,3,4,5-tetrahydro-7,8-dimethoxy-2H-3-benzazepin-2-one **Molecular Formula:** C_27_H_36_N_2_O_5_ **Molecular Weight:** 468.59 **Mechanism:** Ivabradine is a pure heart rate-lowering agent, acting by selective and specific inhibition of the cardiac pacemaker If current that controls the spontaneous diastolic depolarization in the sinus node and regulates heart rate. The cardiac effects are specific to the sinus node with no effect on intra-atrial, atrioventricular or intraventricular conduction times, nor on myocardial contractility or ventricular repolarization. **Effect:** Symptomatic treatment of chronic stable angina pectoris in patients with normal sinus rhythm, who have a contraindication or intolerance for β-blockers.	**Mechanistic genes:***HCN1*, *HCN4* **Metabolic genes** **Substrate:***CYP3A4*, *CYP3A5*
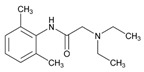	**Name: LIDOCAINE** **IUPAC Name:** Acetamide, 2-(diethylamino)-N-(2,6-dimethylphenyl)-; (2) 2-(diethylamino)-2′,6′-acetoxylidide **Molecular Formula:** C_14_H_22_N_2_O **Molecular Weight:** 234.34 **Mechanism:** Suppresses automaticity of conduction tissue, by increasing electrical stimulation threshold of ventricle, the His–Purkinje system, and spontaneous depolarization of ventricles during diastole by direct action on tissues. **Effect:** Local anesthetic and acute treatment of ventricular arrhythmias (such as from myocardial infarction or cardiac manipulation).	**Mechanistic genes:***CHRM2*, *FOS*, *KCNE2*, *KCNH2*, *KCNJ11*, *KCNQ1*, *SCN5A*, *TRPV1*, *VEGFA* **Metabolic genes** **Substrate:***CYP1A2*, *CYP2A6*, *CYP2B6*, *CYP2C9*, *CYP2D6*, *CYP3A4*, *CYP3A5* **Inhibitor:***ABCB1*, *CYP1A2*, *CYP2D6*, *CYP3A4*, *CYP3A5* **Transporter genes:***ABCC8*, *SLC22A16* **Pleiotropic genes:***IL6*
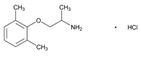	**Name: MEXILETINE** **IUPAC Name:** 2-Propanamine, 1-(2,6-dimethylphenoxy)-, hydrochloride, (±)-; (±)-1-methyl-2-(2,6-xylyloxy)ethylamine hydrochloride **Molecular Formula:** C_11_H_17_NO HCl **Molecular Weight:** 215.72 **Mechanism:** Inhibits inward sodium current, decreases rate of rise of phase 0, increases effective refractory period/action potential duration ratio. **Effect:** Management of serious ventricular arrhythmias. Suppression of premature ventricular contractions.	**Mechanistic genes:***CHRM2*, *KCNE1*, *KCNE2*, *KCNH2*, *KCNJ11*, *KCNQ1*, *SCN5A* **Metabolic genes** **Substrate:***CYP1A2*, *CYP2D6*, *CYP3A4*, *CYP3A5* **Inhibitor:***CYP1A2* **Transporter genes:***ABCC8*
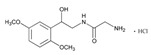	**Name: MIDODRINE** **IUPAC Name:** Acetamide, 2-amino-N-[2-(2,5-dimethoxyphenyl)-2-hydroxyethyl]-, monohydrochloride, (±)-; (±)-2-amino-N-(β-hydroxy-2,5-dimethoxyphenethyl)acetamide monohydrochloride **Molecular Formula:** C_12_H_18_N_2_O_4_ HCl **Molecular Weight:** 290.74 **Mechanism:** Midodrine forms an active metabolite, desglymidodrine, an α1 agonist. This agent increases arteriolar and venous tone resulting in a rise in standing, sitting, and supine systolic and diastolic blood pressure in orthostatic hypotension. **Effect:** Orphan drug: Treatment of symptomatic orthostatic hypotension.	**Mechanistic genes:***ADRA1A*, *ADRA1B* **Metabolic genes** **Substrate:***CYP2D6*
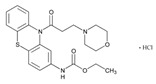	**Name: MORICIZINE** **IUPAC Name:** Carbamic acid, [10-[3-(4-morpholinyl)-l-oxopropyl]-10H-phenothiazin-2yl]-, ethyl ester, hydrochloride; ethyl 10-(3-morpholinopropionyl)phenothiazine-2-carbamate, hydrochloride **Molecular Formula:** C_22_H_25_N_3_O_4_S HCl **Molecular Weight:** 463.98 **Mechanism:** Reduces fast inward current carried by sodium ions, shortens phase I and phase II repolarization, resulting in decreased action potential duration and effective refractory period. **Effect:** Treatment of ventricular tachycardia and life-threatening ventricular arrhythmias.	**Mechanistic genes:***SCN5A* **Metabolic genes** **Substrate:***CYP3A4*, *CYP3A5* **Inducer:***CYP1A2*, *CYP3A4*, *CYP3A5*
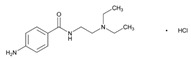	**Name: PROCAINAMIDE** **IUPAC Name:** Benzamide, 4-amino-N-[2-(diethylamino)ethyl]-, monohydrochloride; p-amino-N-[2-(diethylamino)ethyl]benzamide monohydrochloride **Molecular Formula:** C_13_H_21_N_3_O HCl **Molecular Weight:** 271.79 **Mechanism:** Decreases myocardial excitability and conduction velocity and may depress myocardial contractility, by increasing electrical stimulation threshold of ventricle, the His–Purkinje system and through direct cardiac effects. **Effect:** Treatment of ventricular tachycardia, premature ventricular contractions, paroxysmal atrial tachycardia, and atrial fibrillation. Prevention of recurrence of ventricular tachycardia, paroxysmal supraventricular tachycardia, atrial fibrillation or flutter.	**Mechanistic genes:***CHRM2*, *KCNE1*, *KCNE2*, *KCNQ1*, *KCNH2*, *KCNJ11*, *SCN5A* **Metabolic genes** **Substrate:***CYP2D6*, *CYP3A4*, *CYP3A5*, *NAT2* **Transporter genes:***ABCC8*, *SLC22A16*
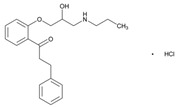	**Name: PROPAFENONE** **IUPAC Name:** 1-Propanone, 1-[2-[2-hydroxy-3-(propylamino)propoxy]phenyl]-3-phenyl-, hydrochloride; 2′-[2-hydroxy-3-(propylamino)propoxy]-3-phenylpropiophenone hydrochloride **Molecular Formula:** C_21_H_27_NO_3_ HCl **Molecular Weight:** 377.90 **Mechanism:** Possesses local anesthetic properties, blocks fast inward sodium current, and slows rate of increase of action potential. Prolongs effective refractory period, reduces spontaneous automaticity and exhibits some β-blockade activity. **Effect:** Treatment of life-threatening ventricular arrhythmias. Maintenance of normal sinus rhythm in symptomatic atrial fibrillation.	**Mechanistic genes:***ADRB*, *CHRM2*, *KCNE1*, *KCNE2*, *KCNH2*, *KCNJ11*, *KCNQ1* **Metabolic genes** **Substrate:***CYP1A1*, *CYP1A2*, *CYP2D6*, *CYP3A4*, *CYP3A5*, *UGTs* **Transporter genes:***ABCC8*
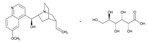	**Name: QUINIDINE** **IUPAC Name:** (8R,9S)-6′-Methoxycinchonan-9-ol **Molecular Formula:** C_20_H_24_N_2_O_2_ **Molecular Weight:** 324.42 **Mechanism:** Depresses phase 0 of action potential. Decreases myocardial excitability and conduction velocity, and myocardial contractility by decreasing sodium influx during depolarization and potassium efflux in repolarization. Additionally reduces calcium transport across cell membrane. Decreases conduction velocity in atria, ventricles, and the His–Purkinje system, and may decrease or cause no change in conduction velocity through the AV node. May suppress atrial fibrillation or flutter. May produce sinus tachycardia via its anticholinergic effects. Has direct negative inotropic effect, but therapeutic plasma concentrations of drug do not usually depress contractility in normal heart. May reduce peripheral resistance and blood pressure by blockade of α-adrenergic receptors and by effects on myocardial contractility. Acts principally as intraerythrocytic schizonticide (has little effect on sporozoites or pre-erythrocytic parasites). Gametocidal against Plasmodium vivax and P. malariae, but not P. falciparum. **Effect:** Prophylaxis after cardioversion of atrial fibrillation and/or flutter to maintain normal sinus rhythm. Prevent recurrence of paroxysmal supraventricular tachycardia, paroxysmal AV junctional rhythm, paroxysmal ventricular tachycardia, paroxysmal atrial fibrillation, and atrial or ventricular premature contractions. Has activity against Plasmodium falciparum malaria.	**Mechanistic genes:***CHRM2*, *CHRNA1*, *FGB*, *G6PD*, *KCNE1*, *KCNE2*, *KCNH2*, *KCNJ11*, *KCNQ1*, *LIPC*, *SCN5A*, *TNFRSF1A* **Metabolic genes** **Substrate:***CYP1A2*, *CYP2C9*, *CYP2D6*, *CYP2E1*, *CYP3A4*, *CYP3A5*, *GSTM1*, *GSTP1* **Inhibitor:***ABCB1*, *CYP2C9*, *CYP2D6*, *CYP3A4*, *CYP3A5* **Transporter genes:***ABCB1*, *ABCC1*, *ABCC8*
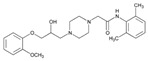	**Name: RANOLAZINE** **IUPAC Name:** 1-Piperazineacetamide, N-(2,6-dimethylphenyl)-4-[2-hydroxy-3-(2-methoxyphenoxy)propyl]-; (±)-N-(2,6-dimethylphenyl)-4-[2-hydroxy-3-(2-methoxyphenoxy)propyl]-1-piperazineacetamide **Molecular Formula:** C_24_H_33_N_3_O_4_ **Molecular Weight:** 427.54 **Mechanism:** Ranolazine exerts antianginal and anti-ischemic effects without changing hemodynamic parameters (heart rate or blood pressure). At therapeutic levels, ranolazine inhibits late phase of inward sodium channel (late INa) in ischemic cardiac myocytes during cardiac repolarization reducing intracellular sodium concentrations and thereby reducing calcium influx via Na+-Ca2+ exchange. Decreased intracellular calcium reduces ventricular tension and myocardial oxygen consumption. It is thought that ranolazine produces myocardial relaxation and reduces anginal symptoms through this mechanism although this is uncertain. At higher concentrations, ranolazine inhibits rapid delayed rectifier potassium current (IKr) thus prolonging ventricular action potential duration and subsequent prolongation of QT interval. **Effect:** Treatment of chronic angina.	**Mechanistic genes:***SCN5A* **Metabolic genes** **Substrate:***CYP2D6*, *CYP3A4*, *CYP3A5* **Inhibitor:***ABCB1*, *CYP2D6*, *CYP3A4*, *CYP3A5* **Transporter genes:***ABCB1*
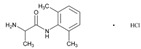	**Name: TOCAINIDE** **IUPAC Name:** Propanamide, 2-amino-N-(2,6-dimethylphenyl)-, hydrochloride, (±)-; (±)-amino-2′,6′-propionoxylidide hydrochloride **Molecular Formula:** C_11_H_16_N_2_O.HCl **Molecular Weight:** 228.72 **Mechanism:** Suppresses automaticity of conduction tissue, by increasing electrical stimulation threshold of ventricle, the His–Purkinje system, and spontaneous depolarization of ventricles during diastole by direct action on tissues. Blocks both initiation and conduction of nerve impulses by decreasing permeability to sodium ions of neuronal membrane, which results in inhibition of depolarization with resultant blockade of conduction. **Effect:** Suppression and prevention of symptomatic life-threatening ventricular arrhythmias.	**Mechanistic genes:***CHRM2*, *KCNE1*, *KCNE2*, *KCNH2*, *KCNJ11*, *KCNQ1*, *SCN5A* **Inhibitor:***CYP1A2* **Transporter genes:***ABCC8*
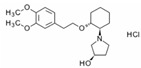	**Name: VERNAKALANT** **IUPAC Name:** (3R)-1-[(1R,2R)-2-[2-(3,4-dimethoxyphenyl)ethoxy]cyclo34enzazepineolidin-3-ol hydrochloride **Molecular Formula:** C_20_H_31_NO_4_ HCl **Molecular Weight:** 385.20 **Mechanism:** Vernakalant is an antiarrhythmic medicine that acts preferentially in the atria to prolong atrial refractoriness and to rate-dependently slow impulse conduction. These antifibrillatory actions on refractoriness and conduction are thought to suppress re-entry, and are potentiated in the atria during atrial fibrillation. The relative selectivity of vernakalant on atrial vs. ventricular refractoriness is postulated to result from the block of currents that are expressed in the atria, but not in the ventricles, as well as the unique electrophysiologic condition of the fibrillating atria. However, blockade of cationic currents, including hERG channels and cardiac voltage-dependent sodium channels present in the ventricles, has been documented. **Effect:** Rapid conversion of recent onset atrial fibrillation to sinus rhythm in adults (for non-surgery patients, atrial fibrillation ≤7 days duration; and for post-cardiac surgery patients, atrial fibrillation ≤3 days duration).	**Mechanistic genes:***KCNA5*, *KCNH2* **Metabolic genes** **Substrate:***CYP2D6*, *UGTs*
**Diuretics**		
**Drug**	**Properties**	**Pharmacogenetics**
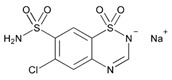	**Name: CHLOROTHIAZIDE** **IUPAC Name:** 2H-1,2,4-benzothiadiazine-7-sulfonamide, 6-chloro-, 1,1-dioxide, monosodium salt; 6-chloro-2H-1,2,4-benzothiadiazine-7-sulfonamide, 1,1-dioxide, monosodium salt **Molecular Formula:** C_7_H_5_ClN_3_NaO_4_S_2_ **Molecular Weight:** 317.71 **Mechanism:** Primary site of diuretic action appears to be the cortical diluting segment of the nephron. Enhances excretion of sodium, chloride, and water by interfering with the transport of sodium ions across the renal tubular epithelium. Enhances urinary excretion of potassium secondary to increased amount of sodium at distal tubular site of sodium–potassium exchange. Increases urinary bicarbonate excretion (although to a lesser extent than chloride excretion) but change in urinary pH is usually minimal (diuretic efficacy not affected by acid–base balance of patient). Increases calcium urinary excretion from a decrease in extracellular fluid volume, although calcium reabsorption in the nephron may be increased (also, slight or intermittent elevations in serum calcium concentration). Hypotensive activity in hypertensive patients (unknown mechanism; potential direct arteriolar dilation). **Effect:** Mild-to-moderate hypertension. Edema.	**Mechanistic genes:***ADD1*, *ADRB1*, *ADRB2*, *ACE1*, *AGT*, *GNB3*, *NOS3*, *SCNN1G*, *WNK1* **Metabolic genes** **Substrate:***CYP11B2*
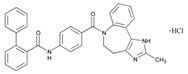	**Name: CONIVAPTAN** **IUPAC Name:** [1,1′-Biphenyl]-2-carboxamide, N-[4-[(4,5-dihydro-2-methylimidazo[4,535enzazepineazepin-6(1H)-yl)carbonyl]phenyl]-, monohydrochloride; 4′’-[(4,5-dihydro-2-methylimidazo[4,535enzazepineazepin-6(1H)-yl)carbonyl]-2-biphenylcarboxanilide monohydrochloride **Molecular Formula:** C_32_H_26_N_4_O_2_ HCl **Molecular Weight:** 535.04 **Mechanism:** Conivaptan is an arginine vasopressin (AVP) receptor antagonist with affinity for AVP receptor subtypes V1A and V2. The antidiuretic action of AVP is mediated through activation of the V2 receptor, which functions to regulate water and electrolyte balance at the level of the collecting ducts in the kidney. Antagonism of the V2 receptor by conivaptan promotes the excretion of free water (without loss of serum electrolytes) resulting in net fluid loss, increased urine output, decreased urine osmolality, and subsequent restoration of normal serum sodium levels. Blockade of vascular V1A receptors may cause splanchnic vasodilation, and thus hypotension or variceal bleeding in patients with cirrhosis (especially those with portal hypertension). **Effect:** Euvolemic and hypervolemic hyponatremia in hospitalized patients.	**Mechanistic genes:***AVPR1A* **Metabolic genes** **Substrate:***CYP3A4*, *CYP3A5* **Inhibitor:***CYP3A4*, *CYP3A5*
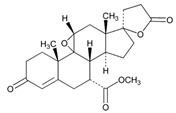	**Name: EPLERENONE** **IUPAC Name:** Pregn-4-ene-7,21-dicarboxylic acid, 9,11-epoxy-17-hydroxy-3-oxo-, γ-lactone, methyl ester, (7α,11α,17α)-; 9,11α-epoxy-17-hydroxy-3-oxo-17α-pregn-4-ene-7α,21-dicarboxylic acid, γ-lactone, methyl ester **Molecular Formula:** C_24_H_30_O_6_ **Molecular Weight:** 414.49 **Mechanism:** A relatively selective competitive mineralocorticoid (aldosterone) receptor antagonist. Binds selectively to mineralocorticoid receptors and has low (less than 1%) affinity for glucocorticoid, progesterone, and androgen receptors. It is a competitive antagonist of aldosterone at mineralocorticoid receptors in the kidney, myocardium, salivary gland, GI tract, brain, and vasculature, and has been shown to inhibit the physiologic effects of aldosterone in these organs. Some of the antihypertensive effects of eplerenone may be related to restoration of endothelial function by increasing the release of nitric oxide, which results in vasodilation. Has been shown to produce sustained increases in plasma renin and serum aldosterone concentrations, reflecting the inhibition of the negative feedback of aldosterone on renin secretion. Appears to have cardioprotective effects in congestive heart failure and left ventricular dysfunction following MI. The cardioprotective action mechanism appears to be related more to the ability of the drug to competitively inhibit the pathophysiologic effects of aldosterone on the myocardium than to its hypotensive effects. Eplerenone reduces coronary vascular inflammation, risk of subsequent development of interstitial myocardial and coronary perivascular fibrosis, cardiac hypertrophy, and/or ventricular remodeling. **Effect:** To reduce the risk of mortality following acute MI in clinically stable patients with left ventricular dysfunction (i.e., LVEF 40% or less) who have demonstrated clinical evidence of CHF. Used orally in the management of hypertension. May be used as monotherapy or in combination with other classes of antihypertensive agents (e.g., ACEIs, angiotensin II receptor antagonists, calcium-channel blocking agents, β-adrenergic blocking agents, and thiazide diuretics).	**Mechanistic genes:***NOS3*, *NPPA*, *NR3C2* **Metabolic genes** **Substrate:***CYP3A4*, *CYP3A5*, *CYP11B2*
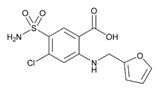	**Name: FUROSEMIDE** **IUPAC Name:** Benzoic acid, 5-(aminosulfonyl)-4-chloro-2-[(2-furanylmethyl)amino]-; 4-chloro-N-furfuryl-5-sulfamoylanthranilic acid **Molecular Formula:** C_12_H_11_ClN_2_O_5_S **Molecular Weight:** 330.74 **Mechanism:** Inhibits reabsorption of sodium and chloride in ascending loop of Henle and distal renal tubule, interfering with chloride-binding cotransport system, thus causing increased excretion of water, sodium, chloride, magnesium, and calcium. **Effect:** Management of edema associated with CHF, nephrotic syndrome, and hepatic cirrhosis. I.V. furosemide may also be used as an adjunct in treatment of acute pulmonary edema.	**Mechanistic genes:***COL1A1*, *FOS*, *GABRA6*, *IFNA1*, *IGF1*, *KDR*, *LTA*, *MMP2*, *NOS3*, *PDGFRA*, *PDGFRB*, *PTGER4*, *PTGS1*, *PTGS2*, *REN*, *SCN1B*, *SCNN1B*, *SCNN1G*, *TGFB1*, *TNFRSF1A*, *TNFRSF1B*, *VCAM1*, *VEGFA* **Metabolic genes** **Substrate:***UGT1A1*, *UGT1A3*, *UGT1A7*, *UGT1A10* **Transporter genes:***ABCC2*, *ABCC3*, *ABCC4*, *SLC12A1*, *SLC12A3*, *SLC22A6*, *SLC22A7* **Pleiotropic genes:***IL6*, *IL10*, *TNF*
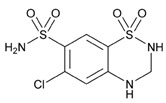	**Name: HYDROCHLOROTHIAZIDE** **IUPAC Name:** 2H-1,2,4-benzothiadiazine-7-sulfonamide, 6-chloro-3,4-dihydro-, 1,1-dioxide; 6-chloro-3,4-dihydro-2H-1,2,4-benzothiadiazine-7-sulfonamide 1,1-dioxide **Molecular Formula:** C_7_H_8_ClN_3_O_4_S_2_ **Molecular Weight:** 297.74 **Mechanism:** Inhibits sodium reabsorption in distal tubules causing increased excretion of sodium and water as well as potassium and hydrogen ions. **Effect:** Management of mild-to-moderate hypertension. Treatment of edema in congestive heart failure and nephrotic syndrome.	**Mechanistic genes:***ACE1*, *ACE2*, *ADD1*, *ADRB1*, *ADRB2*, *AGT*, *GNB3*, *GRIA3*, *NOS3*, *PTGS2*, *REN*, *SCNN1G*, *WNK1* **Metabolic genes** **Substrate:***CYP11B2* **Transporter genes:***ABCC4*, *SLC22A6*
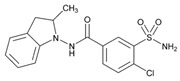	**Name: INDAPAMIDE** **IUPAC Name:** Benzamide, 3-(aminosulfonyl)-4-chloro-N-(2,3-dihydro-2-methyl-1H-indol-1-yl)-; 4-chloro-N-(2-methyl-1-indolinyl)-3-sulfamoylbenzamide **Molecular Formula:** C_16_H_16_ClN_3_O_3_S **Molecular Weight:** 365.83 **Mechanism:** Diuretic effect localized at proximal segment of distal tubule of nephron. Enhances sodium, chloride, and water excretion by interfering with transport of sodium ions across renal tubular epithelium. **Effect:** Management of hypertension, or of edema associated with CHF and nephrotic syndrome.	**Mechanistic genes:***KCNE1*, *KCNQ1* **Metabolic genes** **Substrate:***CYP3A4*, *CYP3A5*
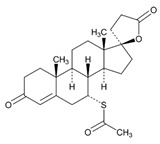	**Name: SPIRONOLACTONE** **IUPAC Name:** Pregn-4-ene-21-carboxylic acid, 7-(acetylthio)-17-hydroxy-3-oxo-, γ-lactone, (7α,17α)-; 17-hydroxy-7α-mercapto-3-oxo-17α-pregn-4-ene-21-carboxylic acid, γ-lactone acetate **Molecular Formula:** C_24_H_32_O_4_S **Molecular Weight:** 416.57 **Mechanism:** Synthetic steroid mineralocorticoid receptor antagonist (aldosterone antagonist). Exhibits magnesium- and potassium-sparing, natriuretic, diuretic, and hypotensive effects by competitively inhibiting physiologic effects of adrenocorticortical hormone aldosterone on distal renal tubules, myocardium, and vasculature. Does not generally cause potassium depletion or affect glucose metabolism or uric acid excretion. Androgen and progesterone receptor antagonist. **Effect:** Edema associated with excessive aldosterone excretion. Hypertension. Primary hyperaldosteronism. Hypokalemia. Cirrhosis accompanied by edema or ascites. Nephritic syndrome. Severe heart failure.	**Mechanistic genes:***ACE1*, *AR*, *NR3C2*, *SCNN1G* **Metabolic genes** **Substrate:***CYP2C8*, *CYP3A4*, *CYP3A5*, *CYP7A1*, *UGT1A1*, *UGT1A6* **Inhibitor:***CYP11B2* **Transporter genes:***ABCB1*, *ABCB11*, *ABCC2*, *ABCC3*
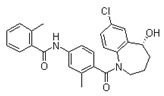	**Name: TOLVAPTAN** **IUPAC Name:** N-[4-(7-chloro-5-hydroxy-2,3,4,5-tetrahydro-1-benzazepine-1-carbonyl)-3-methylphenyl]-2-methylbenzamide; (±)-4′-[(7-chloro-2,3,4,5-tetrahydro-5-hydroxy-1H-1-benzazepin-1-yl)carbonyl]-o-tolu-m-toluidide **Molecular Formula:** C_26_H_25_ClN_2_O_3_ **Molecular Weight:** 448.94 **Mechanism:** Tolvaptan is a selective vasopressin V2-receptor antagonist with an affinity for the V2 receptor that is 1.8-fold that of native arginine vasopressin (AVP). Tolvaptan affinity for the V2 receptor is 29-fold greater than for the V1a receptor. Antagonism of the V2 receptor by tolvaptan promotes the excretion of free water (without loss of serum electrolytes) resulting in net fluid loss, increased urine output, decreased urine osmolality, and subsequent restoration of normal serum sodium levels. Tolvaptan metabolites have no or weak antagonist activity for human V2 receptors compared with tolvaptan. **Effect:** Treatment of clinically significant hypervolemic or euvolemic hyponatremia (associated with heart failure, cirrhosis or SIADH) with either a serum sodium <125 mEq/L or less marked hyponatremia that is symptomatic and resistant to fluid restriction.	**Mechanistic genes:***AVPR2*, *PKD1*, *PKD2* **Metabolic genes** **Substrate:***ABCB1*, *CYP3A4*, *CYP3A5* **Inhibitor:***ABCB1*
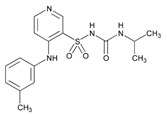	**Name: TORSEMIDE** **IUPAC Name:** 3-Pyridinesulfonamide, N-[[(1-methylethyl)amino]carbonyl]-4-[(3-methylphenyl)amino]-; 1-isopropyl-3-[(4-m-toluidino-3-pyridyl)sulfonyl]urea **Molecular Formula:** C_16_H_20_N_4_O_3_S **Molecular Weight:** 348.42 **Mechanism:** Inhibits reabsorption of sodium and chloride in ascending loop of Henle and distal renal tubule, interfering with chloride-binding cotransport system, thus causing increased excretion of water, sodium, chloride, magnesium, and calcium. **Effect:** Management of edema associated with congestive heart failure and hepatic or renal disease. Used alone or in combination with antihypertensives in treatment of hypertension. I.V. form indicated when rapid onset is desired.	**Mechanistic genes:***ADD1*, *SCNN1G* **Metabolic genes** **Substrate:***CYP2C8*, *CYP2C9*, *CYP11B2* **Inhibitor:***CYP2C19* **Transporter genes:***SLC12A1*, *SLC12A3*, *SLCO1B1*
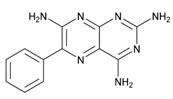	**Name: TRIAMTERENE** **IUPAC Name:** 2,4,7-Pteridinetriamine, 6-phenyl-; (2) 2,4,7-triamino-6-phenylpteridine **Molecular Formula:** C_12_H_11_N_7_ **Molecular Weight:** 253.26 **Mechanism:** Interferes with potassium/sodium exchange (active transport) in distal tubule, cortical collecting tubule and collecting duct by inhibiting sodium, potassium-ATPase. Decreases calcium excretion. Increases magnesium loss. **Effect:** Alone or in combination with other diuretics in treatment of edema and hypertension. Decreases potassium excretion caused by kaliuretic diuretics.	**Mechanistic genes:***SCNN1A*, *SCNN1B*, *SCNN1D*, *SCNN1G* **Metabolic genes** **Substrate:***CYP1A2*, *CYP2A6*, *CYP2D6*, *CYP3A4*, *CYP3A5* **Inducer:***CYP1A2*

***ABCA1*:** ATP-binding cassette, subfamily A, member 1; ***ABCB1*:** ATP-binding cassette, subfamily B, member 1; ***ABCB11*:** ATP-binding cassette, subfamily B, member 11; ***ABCC1*:** ATP-binding cassette, subfamily C, member 1; ***ABCC2*:** ATP-binding cassette, subfamily C, member 2; ***ABCC3*:** ATP-binding cassette, subfamily C, member 3; ***ABCC4*:** ATP-binding cassette, subfamily C, member 4; ***ABCC5*:** ATP-binding cassette, subfamily C, member 5; ***ABCC6*:** ATP-binding cassette, subfamily C, member 6; ***ABCC8*:** ATP-binding cassette, subfamily C, member 8; ***ABCC9*:** ATP-binding cassette, subfamily C, member 9; ***ABCG2*:** ATP-binding cassette, subfamily G, member 2; ***ABL1*:** ABL protooncogene 1, non-receptor tyrosine kinase; ***ACACA*:** Acetyl-CoA carboxylase-alpha; ***ACE1*:** Angiotensin I-converting enzyme; ***ACE2*:** Angiotensin I-converting enzyme 2; ***ACOX1*:** Acyl-CoA oxidase 1, palmitoyl; ***ACSL1*:** Acyl-CoA synthetase long chain family, member 1; ***ACSM1*:** Acyl-CoA synthetase medium chain family member 1; ***ACVRL1*:** Activin A receptor, type II-like 1; ***ADA*:** Adenosine deaminase; ***ADAMTS4*:** A disintegrin-like and metalloproteinase with thrombospondin type 1 motif, 4; ***ADD1*:** Adducin 1; ***ADIPOQ*:** Adipocyte-, C1q-, and collagen domain-containing; ***ADORA1*:** Adenosine A1 receptor; ***ADORA2A*:** Adenosine A2A receptor; ***ADORA2B*:** Adenosine A2b receptor; ***ADRA1A*:** Alpha-1A-adrenergic receptor; ***ADRA1B*:** Alpha-1B-adrenergic receptor; ***ADRA1D*:** Alpha-1D-adrenergic receptor; ***ADRA2A*:** Alpha-2A-adrenergic receptor; ***ADRA2B*:** Alpha-2B-adrenergic receptor; ***ADRA2C***: Alpha-2C-adrenergic receptor; ***ADRB1:*** Beta-1-adrenergic receptor; ***ADRB2***: Beta-2-adrenergic receptor; ***ADRB3*:** Beta-3-adrenergic receptor; ***AGPAT2*:** 1-Acylglycerol-3-phosphate o-acyltransferase 2; ***AGT*:** Angiotensinogen; ***AGTR1*:** Angiotensin II receptor, type 1; ***AGTR2*:** Angiotensin II receptor, type 2; ***AHR*:** Aryl hydrocarbon receptor; ***AKR1C1*:** *A*ldo-keto reductase family 1, member 1; ***AKR1C4*:** Aldo-keto reductase family 1, member C4; ***ALB*:** Albumin; ***ALDH2*:** Aldehyde dehydrogenase 2 family; ***ALDH3A1*:** Aldehyde dehydrogenase, family 3, subfamily A, member 1; ***ALOX5*:** Arachidonate 5-lipoxygenase; ***ANXA1*:** Annexin A1; ***ANXA2*:** Annexin A2; ***ANXA5*:** Annexin A5; ***APC*:** APC regulator of wnt signaling pathway; ***APOA1*:** Apolipoprotein A-I; ***APOA5*:** Apolipoprotein A-V; ***APOB*:** Apolipoprotein B; ***APOC3*:** Apolipoprotein C-III; ***APOE*:** Apolipoprotein E; ***APP*:** Amyloid beta A4 precursor protein; ***AR*:** Androgen receptor; ***ATP1A1*:** ATPase, Na+/K+ transporting, alpha-1 polypeptide; ***AVPR1A*:** Arginine vasopressin receptor 1A; ***AVPR2*:** Arginine vasopressin receptor 2; ***BCAR1*:** BCAR1 scaffold protein, CAS family member; ***BDKRB2*:** Bradykinin receptor B2; ***BMPR2*:** Bone morphogenetic protein receptor, type II; ***CA7*:** Carbonic anhydrase VII; ***CA12*:** Carbonic anhydrase XII; ***CA13*:** Carbonic anhydrase XIII; ***CACNA1C*:** Calcium channel, voltage-dependent, L type, alpha-1C subunit; ***CACNA1D*:** Calcium channel, voltage-dependent, L type, alpha-1D subunit; ***CACNA2D1*:** Calcium channel, voltage-dependent, alpha-2/delta subunit 1; ***CACNB2*:** Calcium channel, voltage-dependent, beta-2 subunit; ***CACNG1*:** Calcium channel, voltage-dependent, gamma-1 subunit; ***CALR*:** Calreticulin; ***CALU*:** Calumenin; **cAMP:** Cyclic adenosine monophosphate; ***CANX*:** Calnexin; ***CASP1*:** Caspase 1, apoptosis-related cysteine protease; ***CASP3*:** Caspase 3, apoptosis-related cysteine protease; ***CAT*:** Catalase; ***CBR1*:** Carbonyl reductase 1; ***CBS*:** Cystathionine beta-synthase; ***CCNA2*:** Cyclin A2; ***CCND1*:** Cyclin D1; ***CDK2*:** Cyclin-dependent kinase 2; ***CDK4*:** Cyclin-dependent kinase 4; ***CEL*:** Carboxyl-ester lipase; ***CES1*:** Carboxylesterase 1; ***CES2*:** Carboxylesterase 2; ***CETP*:** Cholesteryl ester transfer protein, plasma; ***CFH*:** Complement factor H; ***CFTR*:** Cystic fibrosis transmembrane conductance regulator; ***CHAT*:** Choline acetyltransferase; ***CHRM2*:** Cholinergic receptor, muscarinic, 2; ***CHRNA1*:** Cholinergic receptor, nicotinic, alpha polypeptide 1; ***CHRNA2*:** Cholinergic receptor, neuronal nicotinic, alpha polypeptide 2; ***CHRNA4*:** Cholinergic receptor, neuronal nicotinic, alpha polypeptide 4; ***CHST3*:** Carbohydrate sulfotransferase 3; ***CLEC3B*:** Tetranectin; ***CMBL*:** Carboxymethylenebutenolidase-like protein; ***CNP*:** Cyclic nucleotide phosphodiesterase; ***CNR1*:** Cannabinoid receptor 1; ***COL1A1*:** Collagen, type I, alpha-1; ***COMT*:** Catechol-O-methyltransferase; ***COX-1*:** Cycloxygenase-1; ***CPA1*:** Carboxypeptidase A1; ***CREB1*:** cAMP response element-binding protein 1; ***CRP*:** C-reactive protein; ***CRYZ*:** Crystallin, zeta; ***CXCL12*:** Chemokine, CXC motif, ligand 12; ***CYP1A1*:** Cytochrome P450, subfamily I, polypeptide 1; ***CYP1A2*:** Cytochrome P450, subfamily I, polypeptide 2; ***CYP1B1*:** Cytochrome P450, subfamily I, polypeptide 1; ***CYP2A6*:** Cytochrome P450, subfamily IIA, polypeptide 6; ***CYP2B6*:** Cytochrome P450, subfamily IIB, polypeptide 6; ***CYP2C8*:** Cytochrome P450, subfamily IIC, polypeptide 8; ***CYP2C9*:** Cytochrome P450, subfamily IIC, polypeptide 9; ***CYP2C18*:** Cytochrome P450, subfamily IIC, polypeptide 18; ***CYP2C19*:** Cytochrome P450, subfamily IIC, polypeptide 19; ***CYP2D6*:** Cytochrome P450, subfamily IID, polypeptide 6; ***CYP2E1*:** Cytochrome P450, subfamily IIE; ***CYP2J2*:** Cytochrome P450, subfamily IIJ, polypeptide 2; ***CYP3A4*:** Cytochrome P450, subfamily IIIA, polypeptide 4; ***CYP3A5*:** Cytochrome P450, subfamily IIIA, polypeptide 5; ***CYP3A7*:** Cytochrome P450, subfamily IIIA, polypeptide 7; ***CYP4F2*:** Cytochrome P450, family 4, subfamily F, polypeptide 2; ***CYP7A1*:** Cytochrome P450, subfamily VIIA, polypeptide 1; ***CYP11A1*:** Cytochrome P450, subfamily XIA, polypeptide 1; ***CYP11B1*:** Cytochrome P450, Subfamily XIB, polypeptide 1; ***CYP11B2*:** Cytochrome P450, subfamily XIB, polypeptide 2; ***CYP17A1*:** Cytochrome P450, family 17, subfamily A, polypeptide 1; ***CYP19A1*:** Cytochrome P450, family 19, subfamily A, polypeptide 1; ***CYP27A1*:** Cytochrome P450, subfamily XXVIIA, polypeptide 1; ***DDC*:** Dopa decarboxylase; ***DIO2*:** Deiodinase iodothyronine, type II; ***DRD1*:** Dopamine receptor D1; ***DRD2*:** Dopamine receptor D2; ***EDN1*:** Endothelin 1; ***EDNRA*:** Endothelin receptor, type A; ***EDNRB*:** Endothelin receptor, type B; ***EGFR*:** Epidermal growth factor receptor; ***EPHX1*:** Epoxide hydrolase 1; ***ERAP1*:** Endoplasmic reticulum aminopeptidase 1; ***ERBB2***: ERB-B2 receptor tyrosine kinase 2; ***ERBB4*:** ERB-B2 receptor tyrosine kinase 4; ***ESR1*:** Estrogen receptor 1; ***F2*:** Coagulation factor II; ***F2R*:** Coagulation factor II receptor; ***F5*:** Coagulation factor V; ***F7*:** Coagulation factor VII; ***F9*:** Coagulation factor IX; ***F10*:** Coagulation factor X; ***F13A1*:** Factor XIII, A1 subunit; ***FABP1*:** Fatty acid-binding protein 1; ***FABP2*:** Fatty acid-binding protein 2; ***FCGR2A*:** Fc fragment of IgG receptor IIa; ***FCGR2B*:** Fc fragment of IgG receptor IIb; ***FCGR3A*:** Fc fragment of IgG receptor IIIa; ***FGA*:** Fibrinogen, A alpha polypeptide; ***FGB*:** Fibrinogen, B beta polypeptide; ***FGF1*:** Fibroblast growth factor 1; ***FGF2*:** Fibroblast growth factor 2; ***FGF4*:** Fibroblast growth factor 4; ***FGF19*:** Fibroblast growth factor 19; ***FGFR1*:** Fibroblast growth factor receptor 1; ***FGFR2***: Fibroblast growth factor receptor 2; ***FGFR4*:** Fibroblast growth factor receptor 4; ***FKBP5*:** FK506-binding protein 5; ***FOS*:** FOS protooncogene, AP1 transcription factor subunit; ***G6PD*:** Glucose-6-phosphate dehydrogenase; ***GABRA6*:** Gamma-aminobutyric acid receptor, alpha-6; ***GGCX*:** Gamma-glutamyl carboxylase; ***GLUL*:** Glutamate-ammonia ligase; ***GLYAT*:** Glycine-N-acyltransferase; ***GNAS*:** GNAS complex locus; ***GNB3*:** Guanine nucleotide-binding protein, beta-3; ***GP1BA*:** Glycoprotein Ib platelet subunit Alpha; ***GP5*:** Glycoprotein V, platelet; ***GP6*:** Glycoprotein VI platelet; ***GRIA3*:** Glutamate receptor, ionotropic, AMPA 3; ***GRK5*:** G protein-coupled receptor kinase 5; ***GSTA1*:** Glutathione S-transferase, alpha-1; ***GSTK1*:** Glutathione S-transferase, kappa-1; ***GSTM1*:** Glutathione S-transferase, MU-1; ***GSTP1*:** Glutathione S-transferase, PI; ***GSTs*:** Glutathione S-transferase family; ***GSTT1*:** Glutathione S-transferase, theta-1; ***HBB*:** Hemoglobin-beta locus; ***HCII*:** Heparin cofactor II; ***HCN1*:** Hyperpolarization-activated cyclic nucleotide-gated potassium channel 1; ***HCN4*:** Hyperpolarization-activated cyclic nucleotide-gated potassium channel 4; ***HGF*:** Hepatocyte growth factor; ***HFE*:** Homeostatic iron regulator; ***HIF1A*:** Hypoxia-inducible factor 1, alpha subunit; ***HLA-A*:** Major histocompatibility complex, class I, A; ***HLA-B*:** Major histocompatibility complex, class I, B; ***HMGCR*:** 3-Hydroxy-3-methylglutaryl-CoA reductase; ***HNF4A*:** Hepatocyte nuclear factor 4-alpha; ***HPSE*:** *Heparanase; **HRH1:*** Histamine receptor H1; ***HRH2*:** Histamine receptor H2; ***HSPA5*:** *Heat-shock 70-kd protein 5; **HTR1A:*** 5-hydroxytryptamine receptor 1A; ***HTR1B*:** *5*-hydroxytryptamine receptor 1B; ***HTR3B*:** 5-Hydroxytryptamine receptor 3B; ***HTR3C*:** 5-Hydroxytryptamine receptor 3C; ***ICAM1*:** Intercellular adhesion molecule 1; ***IFNA1*:** Interferon, alpha-1; ***IFNG*:** Interferon, gamma; ***IGF1*:** Insulin-like growth factor I; ***IKBKB*:** *Inhibitor of nuclear factor kappa-B kinase*, *subunit beta; **IL1B:*** Interleukin 1-beta; ***IL1RN*:** Interleukin 1 receptor antagonist; ***IL2*:** Interleukin 2; ***IL4*:** Interleukin 4; ***IL6***: Interleukin 6; ***IL8RB*:** Interleukin 8 receptor, beta; ***IL10*:** Interleukin 10; ***IL12B*:** Interleukin 12B; ***ITGA2B*:** Integrin, alpha-2b; ***ITGA4*:** Integrin, alpha-4; ***ITGB3*:** Integrin, beta-3; ***KCNA5*:** Potassium channel, voltage-gated, shaker-related subfamily, member 5; ***KCNE1*:** Potassium channel, voltage-gated, isk-related subfamily, member 1; ***KCNE2*:** Potassium channel, voltage-gated, isk-related subfamily, member 2; ***KCNH2*:** Potassium channel, voltage-gated, subfamily H, member 2; ***KCNJ1*:** Potassium channel, inwardly rectifying, subfamily J, member 1; ***KCNJ5*:** Potassium channel, inwardly rectifying, subfamily J, member 5; ***KCNJ11*:** Potassium channel, inwardly rectifying, subfamily J, member 11; ***KCNMB1*:** Potassium channel, calcium-activated, large conductance, subfamily M, beta member 1; ***KCNQ1*:** Potassium channel, voltage-gated, KQT-like subfamily, member 1; ***KDR*:** Kinase insert domain receptor; ***KRT8*:** Keratin 8, type II; ***LDLR*:** Low-density lipoprotein receptor; ***LEP*:** Leptin; ***LIPA*:** Lipase A, lysosomal acid; ***LIPC*:** Lipase, hepatic; ***LMW*:** Low molecular weight; ***LPL*:** Lipoprotein lipase; ***LRP1*:** Low-density lipoprotein receptor-related protein 1; ***LRP2*:** Low-density lipoprotein receptor-related protein 2; ***LTA*:** Lymphotoxin-alpha; ***MAOA*:** Monoamine oxidase A; ***MAOB*:** Monoamine oxidase B; ***MAP2K4*:** Mitogen-activated protein kinase kinase 4; ***MAPT*:** Microtubule-associated protein tau; ***MGMT*:** Methylguanine-DNA methyltransferase; ***MMP2*:** Matrix metalloproteinase 2; ***MMP3*:** Matrix metalloproteinase 3; ***MMP12*:** Matrix metalloproteinase 12; ***MPO*:** Myeloperoxidase; ***MTHFR*:** 5,10-Methylenetetrahydrofolate reductase; ***MT-ND4*:** Complex I, subunit ND4; ***MTR*:** 5-Methyltetrahydrofolate-homocysteine S-methyltransferase; ***MTTP*:** Microsomal triglyceride transfer protein; ***MYC*:** MYC protooncogene, bHLH transcription factor; ***NAT2*:** N-acetyltransferase 2; ***NFKB1***: Nuclear factor kappa-B, subunit 1; ***NFKBIA*:** *Nuclear factor kappa-B inhibitor*, *alpha;* ***NID1*:** Nidogen 1; ***NNMT*:** Nicotinamide N-methyltransferase; ***NOS1AP*:** Nitric oxide synthase 1 (neuronal) adaptor protein; ***NOS2*:** Nitric oxide synthase 2; ***NOS3*:** Nitric oxide synthase 3; ***NOX1*:** NADPH oxidase 1; ***NPC1*:** NPC intracellular cholesterol transporter 1; ***NPC1L1*:** NPC1-like intracellular cholesterol transporter 1; ***NPPA*:** Natriuretic peptide precursor A; ***NPR1*:** Natriuretic peptide receptor a/guanylate cyclase A; ***NPR2*:** Natriuretic peptide receptor 2; ***NPR3*:** Natriuretic peptide receptor 3; ***NPY*:** Neuropeptide Y; ***NQO1*:** NAD(P)H dehydrogenase, quinone 1; ***NR1I2*:** Nuclear receptor subfamily 1, group I, member 2; ***NR1I3*:** Nuclear receptor subfamily 1, group I, member 3; ***NR3C1*:** Nuclear receptor subfamily 3, group C, member 1; ***NR3C2*:** Nuclear receptor subfamily 3, group C, member 2; ***ORM1*:** *Orosomucoid 1; **P2RY1***: Purinergic receptor P2Y, g protein-coupled, 1; ***P2RY12*:** *Purinergic receptor P2RY12; **PAF-1:** Plasminogen activator inhibitor-1; **PAR-1:** Protease-activated receptor 1; **PARK2:*** Parkin; ***PCNA*:** Proliferating cell nuclear antigen; ***PDE1C*:** Phosphodiesterase 1C; ***PDE3A*:** Phosphodiesterase 3A; ***PDE4A*:** Phosphodiesterase 4A; ***PDE4B*:** Phosphodiesterase 4B; ***PDE4C*:** Phosphodiesterase 4C; ***PDE4D*:** Phosphodiesterase 4D; ***PDE5A*:** Phosphodiesterase 5A; ***PDE10A*:** Phosphodiesterase 10A; ***PDGFR*:** Platelet-derived growth factor receptor; ***PDGFRA*:** Platelet-derived growth factor receptor, alpha; ***PDGFRB*:** Platelet-derived growth factor receptor, beta; ***PF4*:** Platelet factor 4; ***PGD2*:** Prostaglandin D2; ***PGE2*:** Prostaglandin E2; ***PGI2*:** Prostacyclin; ***PKA*:** Protein kinase A; ***PKD1*:** Polycystin 1; ***PKD2*:** Polycystin 2; ***PLA2G4A*:** Phospholipase A2, group IVA; ***PLA2s*:** Phospholipase A2 family; ***PLAT*:** Plasminogen activator, tissue; ***PLAU*:** Plasminogen activator, urinary; ***PLAUR*:** Plasminogen activator receptor, urokinase-type; ***PLG*:** Plasminogen; ***POMC*:** Proopiomelanocortin; ***PON1*:** Paraoxonase 1; ***POR*:** Cytochrome P450 oxidoreductase; ***PPARA*:** Peroxisome proliferator-activated receptor-alpha; ***PPARD*:** Peroxisome proliferator-activated receptor-delta; ***PPARG:*** Peroxisome proliferator-activated receptor-gamma; ***PPARGC1A*:** Peroxisome proliferator-activated receptor-gamma, coactivator 1, alpha; ***PRKRA*:** Protein kinase, interferon-inducible double-stranded MA-dependent activator; ***PRNP*:** Prion protein; ***PROC*:** Protein C; ***PROCR*:** Protein c receptor; ***PROS1*:** Protein S; ***PSEN1*:** Presenilin 1; ***PTGDR2*:** Prostaglandin D2 receptor 2; ***PTGER1*:** Prostaglandin E receptor 1; ***PTGER2*:** Prostaglandin E receptor 2, EP2 subtype; ***PTGER3*:** Prostaglandin E receptor 3, EP3 subtype; ***PTGER4*:** Prostaglandin E receptor 4, EP4 subtype; ***PTGES*:** Prostaglandin E synthase; ***PTGIR*:** Prostaglandin I2 receptor; ***PTGIS1*:** Prostaglandin-endoperoxide synthase 1; ***PTGIS*:** Prostaglandin I2 synthase; ***PTGIS2*:** Prostaglandin-endoperoxide synthase 2; ***PTGS1*:** Prostaglandin-endoperoxide synthase 1; ***PTGS2*:** Prostaglandin-endoperoxide synthase 2; ***RAP1B***: Ras family small GTP binding protein RAP1B; ***RB1*:** RB transcriptional corepressor 1; ***RCAN1*:** Regulator of calcineurin 1; ***REN*:** Renin; ***RET*:** RET protooncogene; ***RPS6KA3***: Ribosomal protein S6 kinase A3; ***RYR1*:** Ryanodine receptor 1; ***SCARB1*:** Scavenger receptor class B, member 1; ***SCN1B*:** Sodium voltage-gated channel, beta subunit 1; ***SCN5A*:** Sodium voltage-gated channel, alpha subunit 5; ***SCN10A*:** Sodium voltage-gated channel, alpha subunit 10; ***SCNN1A*:** Sodium channel, epithelial 1, alpha subunit; ***SCNN1B*:** Sodium channel, epithelial 1, beta subunit; ***SCNN1D*:** Sodium channel, epithelial 1, delta subunit; ***SCNN1G*:** Sodium channel, epithelial 1, gamma subunit; ***SELP*:** Selectin P; ***SERPINA5*:** Serpin peptidase inhibitor, clade A, member 5; ***SERPINA6*:** Serpin peptidase inhibitor, clade A, member 6; ***SERPINA7***: Serpin peptidase inhibitor, clade A, member 7; ***SERPINB2***: Serpin peptidase inhibitor, clade B (ovalbumin), member 2; ***SERPINB6***: Serpin peptidase inhibitor, clade B (ovalbumin), member 6; ***SERPINC1***: Serpin peptidase inhibitor, clade C (antithrombin), member 1; ***SERPIND1***: Heparin cofactor II; ***SERPINE1***: Serpin peptidase inhibitor, clade E (nexin, plasminogen activator inhibitor type 1), member 1; ***SHBG*:** Sex hormone-binding globulin; ***SLC5A5*:** Solute carrier family 5 (sodium iodide symporter), member 5; ***SLC5A8*:** Solute carrier family 5 (iodide transporter), member 8; ***SLC6A2*:** Solute carrier family 6 (neurotransmitter transporter, noradrenaline), member 2; ***SLC6A3*:** Solute carrier family 6 (neurotransmitter transporter, noradrenaline), member 3; ***SLC10A1*:** Solute carrier family 10 (sodium/bile acid cotransporter family), member 1; ***SLC12A1*:** Solute carrier family 12 (sodium/potassium/chloride transporter), member 1; ***SLC12A3*:** Solute carrier family 12 (sodium/chloride transporter), member 3; ***SLC14A2*:** Solute carrier family 14 (urea transporter), member 2; ***SLC15A1*:** Solute carrier family 15 (oligopeptide transporter), member 1; ***SLC18A2*:** Solute carrier family 18 (vesicular monoamine), member 2; ***SLC19A1*:** Solute carrier family 19 (folate transporter), member 1; ***SLC22A1*:** Solute carrier family 22 (organic cation transporter), member 1; ***SLC22A2*:** Solute carrier family 22 (organic cation transporter), member 2; ***SLC22A6*:** Solute carrier family 22 (organic anion transporter), member 6; ***SLC22A7*:** Solute carrier family 22 (organic anion transporter), member 7; ***SLC22A8*:** Solute carrier family 22 (organic anion transporter), member 8; ***SLC22A16*:** Solute carrier family 22 (organic cation transporter), member 16; ***SLCO1A2*:** Solute carrier organic anion transporter family, member 1A2; ***SLCO1B1*:** Solute carrier organic anion transporter family, member 1B1; ***SLCO1B2*:** Solute carrier organic anion transporter family, member 1B2; ***SLCO1B3*:** Solute carrier organic anion transporter family, member 1B3; ***SLCO2A1*:** Solute carrier organic anion transporter family, member 2A1; ***SLCO2B1:*** Solute carrier organic anion transporter family, member 2B1; ***SLCO3A1*:** Solute carrier organic anion transporter family, member 3A1; **S*NCA*:** Synuclein, alpha; ***SOD2*:** Superoxide dismutase 2; ***ST14*:** Suppression of tumorigenicity 14; ***STAT3*:** Signal transducer and activator of transcription 3; ***SULT1A1*:** Sulfotransferase family 1A, cytosolic, phenol-preferring, member 1; ***SULT1A3*:** Sulfotransferase family 1A, cytosolic, phenol-preferring, member 3; ***SULT1E1*:** Sulfotransferase family 1E, estrogen-preferring, member 1; ***TBX21*:** T-box transcription factor 21; ***TBXA2R*:** Thromboxane A2 receptor; ***TBXAS1*:** Thromboxane A synthase 1; ***TCF20*:** Transcription factor 20; ***TFPI*:** Tissue factor pathway inhibitor; ***TGFB1*:** Transforming growth factor, beta-1; ***TGFBR1*:** Transforming growth factor-beta receptor, type I; ***THBD*:** Thrombomodulin; ***TNF***: Tumor necrosis factor; ***TNFAIP6*:** Tumor necrosis factor-alpha-induced protein 6; ***TNFRSF1A*:** Tumor necrosis factor Receptor superfamily, member 1A; ***TNFRSF1B*:** Tumor necrosis factor receptor subfamily, member 1B; ***TP53*:** Tumor protein p53; ***TPMT*:** Thiopurine s-methyltransferase; ***TRAP*:** Thrombin receptor agonist peptide; ***TRPV1*:** Transient receptor potential cation channel, subfamily v, member 1; ***UCP2*:** Uncoupling protein 2; ***UGT1A1*:** UPD-glycosyltransferase 1 family, polypeptide A1; ***UGT1A3*:** UPD-glycosyltransferase 1 family, polypeptide A3; ***UGT1A4*:** UDP-glycosyltransferase 1 family, polypeptide A4; ***UGT1A5*:** UDP-glycosyltransferase 1 family, polypeptide A5; ***UGT1A6*:** UDP-glycosyltransferase 1 family, polypeptide A6; ***UGT1A7*:** UDP-glycosyltransferase 1 family, polypeptide A7; ***UGT1A9*:** UDP-glycosyltransferase 1 family, polypeptide A9; ***UGT1A10*:** UDP-glycosyltransferase 1 family, polypeptide A10; ***UGT2B4*:** UDP glucuronosyltransferase family 2, member B4; ***UGT2B7*:** Uridine diphosphate glycosyltransferase 2 family, member B7; ***UGT2B15*:** Uridine diphosphate glycosyltransferase 2 family, member B15; ***UGT2B17*:** Uridine diphosphate glycosyltransferase 2 family, member B17; ***UGTs*:** UDP glucuronosyltransferase family; ***USP5*:** Ubiquitin-specific protease 5; ***VCAM1*:** Vascular cell adhesion molecule 1*; **VEGFA:*** Vascular endothelial growth factor A; ***VKORC1*:** Vitamin K epoxide reductase complex subunit 1; ***VTN*:** Vitronectin; ***VWF*:** von Willebrand factor; ***WNK1*:** Protein kinase, lysine-deficient 1; ***XDH*:** Xanthine dehydrogenase.

**Table 2:** Pharmacogenetics of lipid-modifying agents.

Lipid-Modifying Agents		
Drug	Properties	Pharmacogenetics
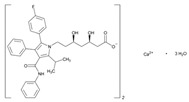	**Name: ATORVASTATIN** **IUPAC Name:** 1H-Pyrrole-1-heptanoic acid, 2-(4-fluorophenyl)-β,δ-dihydroxy-5-(1-methylethyl)-3-phenyl-4-[(phenylamino)carbonyl]-, calcium salt (2:1), [R-(R*,R*)]-; calcium (βR,δR)-2-(p-fluorophenyl)-β,δ-dihydroxy-5-isopropyl-3-phenyl-4-(phenylcarbamoyl)pyrrole-1-heptanoate (1:2) **Molecular Formula:** C_66_H_68_CaF_2_N_4_O_10_ **Molecular Weight:** 1155.34 **Mechanism:** Inhibits 3-hydroxy-3-methylglutaryl coenzyme A (HMG-CoA) reductase, resulting in a compensatory increase in the expression of LDL receptors on hepatocyte membranes and a stimulation of LDL catabolism. **Effect:** Treatment of dyslipidemias or primary prevention of cardiovascular disease (atherosclerotic).	**Mechanistic genes:** *APOA5*, *APOB*, *APOC3*, *ACE1*, *APOA1*, *APOE*, *CETP*, *CRP*, *ESR1*, *FGB*, *GNB3*, *HTR3B*, *ITGB3*, *LDLR*, *LIPC*, *MMP3*, *MTTP*, *NOS3*, *PON1*, *USP5* **Metabolic genes** **Substrate:** *CYP2C8*, *CYP2C9*, *CYP3A4*, *CYP3A5*, *CYP7A1*, *CYP11B2*, *UGT1A1*, *UGT1A3* **Inhibitor:** *ABCB1*, *CYP2B6*, *CYP2C9*, *CYP2C19*, *CYP2D6*, *CYP3A4*, *CYP3A5*, *HMGCR* **Inducer:** *CYP2B6* **Transporter genes:** *ABCA1*, *ABCB1*, *ABCB11*, *ABCC1*, *ABCC2*, *ABCC3*, *ABCG2*, *SLC16As*, *SLCO1B1*, *SLCO1B3* **Pleiotropic genes:** *IL6*, *IL10*, *TNF*
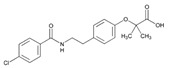	**Name: BEZAFIBRATE** **IUPAC Name:** Propanoic acid, 2-[4-[2-[(4-chlorobenzoyl)amino]ethyl]phenoxy]-2-methyl-; 2-[p-[2-(p-chlorobenzamido)ethyl]phenoxy]-2-methylpropionic acid **Molecular Formula:** C_19_H_20_ClNO_4_ **Molecular Weight:** 361.82 **Mechanism:** Mechanism not established. May increase VLDL catabolism by increasing lipoprotein and hepatic triglyceride lipase activities. May decrease triglyceride biosynthesis by inhibition of acetyl-CoA carboxylase. May decrease cholesterol biosynthesis by inhibition of 3-hydroxy-3-methyglutaryl-coenzyme A reductase. **Effect:** Adjunct to diet and other therapeutic measures for treatment of type IIa and IIb mixed hyperlipidemia, to regulate lipid and apoprotein levels (reduce serum triglycerides, LDL-C and apolipoprotein B, increase HDL-C and apolipoprotein A). Treatment of adult patients with high to very high triglyceride levels (hypertriglyceridemia)(Fredrickson classification type IV and V hyperlipidemias) who are at high risk of complications from their dyslipidemia.	**Mechanistic genes:***ACSL1*, *ACOX1*, *APOA5*, *APOB*, *APOC3*, *APOE*, *CETP*, *LDLR*, *LIPC*, *MGMT*, *PPARA*, *SCARB1* **Metabolic genes** **Substrate:***CYP1A1*, *CYP3A4*, *CYP3A5*, *CYP7A1*, *UGT1A1* **Inhibitor:***ACACA*, *CYP2C8*, *HMGCR* **Inducer:***CYP3A4*, *CYP3A5*, *UGT1A1* **Transporter genes:***ABCB11*
	**Name: COLESTIPOL** **IUPAC Name:** Colestipol hydrochloride; copolymer of diethylenetriamine and 1-chloro-2,3-epoxypropane, hydrochloride **Molecular Formula:** N.A. **Molecular Weight:** N.A. **Mechanism:** Binds to bile acids in the intestine and forms a non-absorbable complex. Thus, bile acids are partially removed from enterohepatic circulation and conversion of cholesterol to bile acids in the liver is increased. This enhanced demand for cholesterol in liver cells causes a compensatory increase in hepatic uptake (and thus systemic clearance) of circulating LDL-C. Serum triglyceride concentrations may remain unchanged or increase slightly (5–10%). Antilipemic effects are additive when used with lovastatin or niacin. **Effect:** Primary hypercholesterolemia. Arteriolosclerosis. Pruritus associated with elevated levels of bile acids. To decrease plasma half-life of digoxin in toxicity.	**Mechanistic genes:***APOE*, *LIPC* **Metabolic genes** **Substrate:***CYP7A1*
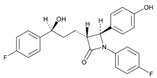	**Name: EZETIMIBE** **IUPAC Name:** 2-Azetidinone, 1-(4-fluorophenyl)-3-[3-(4-fluorophenyl)-3-hydroxypropyl]-4-(4-hydroxyphenyl)-, [3R-[3α(S*),4β]]-; (2)(3R,4S)-1-(p-fluorophenyl)-3-[(3S)-3-(p-fluorophenyl)-3-hydroxypropyl]-4-(p-hydroxyphenyl)-2-azetidinone **Molecular Formula:** C_24_H_21_F_2_NO_3_ **Molecular Weight:** 409.43 **Mechanism:** Inhibits absorption of cholesterol at the brush border of the small intestine via the sterol transporter, Niemann–Pick C1-Like 1 (NPC1L1). This leads to a decreased delivery of cholesterol to the liver, reduction in hepatic cholesterol stores and increased clearance of cholesterol from the blood. Decreases total cholesterol, LDL-C, APOB, and triglycerides while increasing HDL-C. **Effect:** Used in combination with dietary therapy for treatment of primary hypercholesterolemia (as monotherapy or in combination with HMG-CoA reductase inhibitors). Homozygous sitosterolemia. Homozygous familial hypercholesterolemia (in combination with atorvastatin or simvastatin). Mixed hyperlipidemia (in combination with fenofibrate).	**Mechanistic genes:***APOE*, *APOA1*, *APOA5*, *APOB*, *APOC3*, *CETP*, *NPC1*, *NPC1L1*, *SCARB1* **Metabolic genes** **Substrate:***CYP7A1*, *UGT1A1*, *UGT1A3*, *UGT2B7*, *UGT2B15* **Transporter genes:***ABCB1*, *ABCA1*, *ABCC2*, *SLCO1B1*, *SLCO1B3*
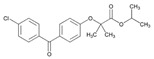	**Name: FENOFIBRATE** **IUPAC Name:** Isopropyl 2-[p-(p-chlorobenzoyl)phenoxy]-2-methylpropionate **Molecular Formula:** C_20_H_21_ClO_4_ **Molecular Weight:** 360.83 **Mechanism:** Fenofibric acid is believed to increase VLDL catabolism by enhancing the synthesis of lipoprotein lipase. As a result of a decrease in VLDL levels, total plasma triglycerides are reduced by 30%–60%. Modest increase in HDL occurs in some hypertriglyceridemic patients. **Effect:** Adjunct to dietary therapy to decrease elevated serum total and LDL-C, triglyceride, and apolipoprotein B (APOB) concentrations, and to increase HDL-C concentrations in the management of primary hypercholesterolemia and mixed dyslipidemia, including heterozygous familial hypercholesterolemia and other causes of hypercholesterolemia. Additive antilipemic effects when used concomitantly with other antilipemic agents (e.g., colesevelam and ezetimibe). Adjunct to dietary therapy in management of hypertriglyceridemia.	**Mechanistic genes:***ACE1*, *ACOX1*, *APOA1*, *APOA5*, *APOB*, *APOE*, *FABP1*, *FABP2*, *LPL*, *PPARA*, *SCARB1* **Metabolic genes** **Substrate:***CYP2C8*, *CYP3A4*, *CYP3A5*, *UGT1A1* **Inhibitor:***CYP2A6*, *CYP2C8*, *CYP2C9*, *CYP2C19*, *CYP4F2* **Inducer:***CYP2C8*, *CYP3A4*, *CYP3A5*, *UGT1A1* **Transporter genes:***ABCA1*, *ABCB1* **Pleiotropic genes:***APP*, *IL6*
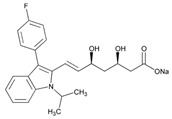	**Name: FLUVASTATIN** **IUPAC Name:** 6-Heptenoic acid, 7-[3-(4-fluorophenyl)-1-(1-methylethyl)-1H-indol-2-yl]-3,5-dihydroxy-, monosodium salt, [R*,S*-(E)]-(±)-; sodium (±)-(3R*,5S*,6E)-7-[3-(p-fluorophenyl)-1-isopropylindol-2-yl]-3,5-dihydroxy-6-heptenoate **Molecular Formula:** C_24_H_25_FNNaO_4_ **Molecular Weight:** 433.45 **Mechanism:** Acts by competitively inhibiting 3-hydroxyl-3-methylglutaryl-coenzyme A (HMG-CoA) reductase (HMGCR), the enzyme that catalyzes reduction in HMG-CoA to mevalonate. HDL is increased while total, LDL and VLDL cholesterols, apolipoprotein B, and plasma triglycerides are decreased. **Effect:** Used as a component of multiple risk factor intervention in patients at risk for atherosclerosis vascular disease due to hypercholesterolemia.	**Mechanistic genes:***ACE1*, *APOA1*, *APOA5*, *APOB*, *APOC3*, *APOE*, *CETP*, *LDLR*, *LIPC*, *LPL*, *NOS3*, *NR1I2*, *NR1I3*, *PPARD*, *PON1*, *USP5* **Metabolic genes** **Substrate:***CYP1A1*, *CYP2B6*, *CYP2C8*, *CYP2C9*, *CYP2D6*, *CYP3A4*, *CYP3A5*, *CYP7A1*, *UGT1A3* **Inhibitor:***CYP1A2*, *CYP2C8*, *CYP2C9*, *CYP2C19*, *CYP2D6*, *CYP3A4*, *CYP3A5*, *HMGCR* **Transporter genes:***ABCA1*, *ABCB1*, *ABCB11*, *ABCC2*, *ABCG2*, *SLC15A1*, *SLC22A8*, *SLCO1B1*, *SLCO1B3*, *SLCO2B1*
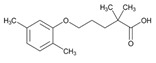	**Name: GEMFIBROZIL** **IUPAC Name:** Pentanoic acid, 5-(2,5-dimethylphenoxy)-2,2-dimethyl-; 2,2-dimethyl-5-(2,5-xylyloxy)valeric acid **Molecular Formula:** C_15_H_22_O_3_ **Molecular Weight:** 250.33 **Mechanism:** Gemfibrozil can inhibit lipolysis and decrease subsequent hepatic fatty acid uptake as well as inhibit hepatic secretion of VLDL. Together, these actions decrease serum VLDL levels. Increases HDL-C. **Effect:** Gemfibrozil is used to reduce risk of developing coronary heart disease (CHD) in patients with type IIb hyperlipoproteinemia without clinical evidence of CHD (primary prevention) who have inadequate response to dietary management, weight loss, exercise, and drugs known to reduce LDL-C and increase HDL-C and who have low HDL-C concentrations in addition to elevated LDL-C and triglycerides. In addition, gemfibrozil is used for treatment of hypertriglyceridemia in types IV and V hyperlipidemia for patients at greater risk for pancreatitis and who have not responded to dietary intervention.	**Mechanistic genes:***ACE1*, *ACOX1*, *AHR*, *APOE*, *CES2*, *CETP*, *CFTR*, *LPL*, *MMP3*, *NR1I2*, *PPARA*, *SCARB1* **Metabolic genes** **Substrate:***CYP2D6*, *CYP2C8*, *CYP3A4*, *CYP3A5*, *CYP7A1*, *UGT1A1*, *UGT1A3* **Inhibitor:***CYP1A2*, *CYP2C8*, *CYP2C9*, *CYP2C19*, *CYP3A4*, *CYP3A5* **Inducer:***CYP2C8*, *UGT1A1* **Transporter genes:***ABCB1*, *SLCO1B1*, *SLCO1B2* **Pleiotropic genes:***IL1B*, *IL12B*, *TNF*
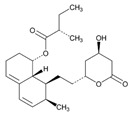	**Name: LOVASTATIN** **IUPAC Name:** Butanoic acid, 2-methyl-, 1,2,3,7,8,8a-hexahydro-3,7-dimethyl-8-[2-(tetrahydro-4-hydroxy-6-oxo-2H-pyran-2-yl)ethyl]-1-naphthalenyl ester, [1S-[1α(R*),3α,7β,8β(2S*,4S*),8aβ]]-; (S)-2-methylbutyric acid, 8-ester with (4R,6R)-6-[2-[(1S,2S,6R,8S,8aR)-1,2,6,7,8,8a-hexahydro-8-hydroxy-2,6-dimethyl-1-naphthyl]ethyl]tetrahydro-4-hydroxy-2H-pyran-2-one **Molecular Formula:** C_24_H_36_O_5_ **Molecular Weight:** 404.54 **Mechanism:** Acts by competitively inhibiting 3-hydroxyl-3-methylglutaryl-coenzyme A (HMG-CoA) reductase, enzyme which catalyzes rate-limiting step in cholesterol biosynthesis. **Effect:** Adjunct to dietary therapy to decrease elevated serum total and LDL-C concentrations in primary hypercholesterolemia. Primary prevention of coronary artery disease (patients without symptomatic disease with average to moderately elevated total and LDL-C and below average HDL-C). Slow progression of coronary atherosclerosis in coronary heart disease. Adjunct to dietary therapy in adolescent patients (10–17 years of age, females >1 year postmenarche) with heterozygous familial hypercholesterolemia having LDL>189 mg/dL, or LDL>160 mg/dL with positive family history of premature cardiovascular disease, or LDL>160 mg/dL with presence of at least two other CVD risk factors.	**Mechanistic genes:***APOA1*, *APOA5*, *APOB*, *APOC3*, *CETP*, *KCNH2*, *LDLR*, *LIPC*, *LPL*, *USP5* **Metabolic genes** **Substrate:***CYP2C8*, *CYP3A4*, *CYP3A5*, *UGT1A3* **Inhibitor:***ABCB1*, *CYP2C9*, *CYP2C19*, *CYP2D6*, *CYP3A4*, *CYP3A5*, *HMGCR* **Inducer:***CYP2B6*, *CYP7A1* **Transporter genes:***ABCA1*, *ABCB1*, *ABCB11*, *ABCC2*, *ABCG2*, *SLCO1B1*, *SLCO1B3* **Pleiotropic genes:***TP53*
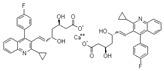	**Name: PITAVASTATIN** **IUPAC Name:** Calcium (E,3R,5S)-7-[2-cyclopropyl-4-(4-fluorophenyl)quinolin-3-yl]-3,5-dihydroxyhept-6-enoate; (+)monocalcium bis{(3R, 5S, 6E)-7-[2-cyclopropyl-4-(4-fluorophenyl)-3-quinolyl]-3,5-dihydroxy-6-heptenoate} **Molecular Formula:** C_50_H_46_CaF_2_N_2_O_8_ **Molecular Weight:** 880.98 **Mechanism:** Pitavastatin competitively inhibits HMG-CoA reductase, which is a rate-determining enzyme involved with biosynthesis of cholesterol, in a manner of competition with the substrate so that it inhibits cholesterol synthesis in the liver. As a result, the expression of LDL receptors followed by the uptake of LDL from blood to liver is accelerated and then the plasma total cholesterol (TC) decreases. Furthermore, sustained inhibition of cholesterol synthesis in the liver decreases levels of very low-density lipoproteins. **Effect:** Adjunct to dietary therapy to reduce elevations in TC, LDL-C, apolipoprotein B (Apo B), and triglycerides (TG), and to increase low HDL-C in patients with primary hyperlipidemia and mixed dyslipidemia.	**Metabolic genes** **Substrate:***CYP2C8*, *CYP2C9*, *CYP3A4*, *CYP3A5*, *UGT1A3*, *UGT2B7* **Inhibitor:***HMGCR* **Transporter genes:***ABCB1*, *ABCG2*, *SLCO1B1*
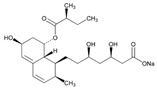	**Name: PRAVASTATIN** **IUPAC Name:** 1-Naphthaleneheptanoic acid, 1,2,6,7,8,8a-hexahydro-β,δ,6-trihydroxy-2-methyl-8-(2-methyl-1-oxobutoxy)-, monosodium salt, [1S-[1α(βS*,δS*),2α,6α,8β(R*),8aα]]-; sodium (+)-(βR,δR,1S,2S,6S,8S,8aR)-1,2,6,7,8,8a-hexahydro-β,δ,6,8-tetrahydroxy-2-methyl-1-naphthaleneheptanoate, 8-[(2S)-2-methylbutyrate] **Molecular Formula:** C_23_H_35_NaO_7_ **Molecular Weight:** 446.51 **Mechanism:** A competitive inhibitor of 3-hydroxy-3-methylglutaryl coenzyme A (HMG-CoA) reductase. **Effect:** Primary prevention of coronary events (reduction in cardiovascular morbidity (myocardial infarction, coronary revascularization procedures) and mortality). Secondary prevention of cardiovascular events in established coronary heart disease (slowing of progression of coronary atherosclerosis, reduction in cardiovascular morbidity (myocardial infarction, coronary vascular procedures) and reduction in mortality, reduction in risk of stroke and transient ischemic attacks). Hyperlipidemias (reduction in elevations in total cholesterol, LDL-C, apolipoprotein B, and triglycerides). Heterozygous familial hypercholesterolemia.	**Mechanistic genes:***ACE1*, *ALDH1A1*, *APOA1*, *APOA5*, *APOB*, *APOC3*, *APOE*, *CBS*, *CETP*, *CRP*, *FGB*, *HMGCR*, *HTR3B*, *ITGB3*, *LDLR*, *LEP*, *LIPC*, *LPL*, *MMP2*, *MMP3*, *MTHFR*, *NOS3*, *PON1*, *USP5* **Metabolic genes** **Substrate:***CYP1A1*, *CYP1A2*, *CYP2C8*, *CYP2E1*, *CYP3A4*, *CYP3A5*, *CYP7A1*, *UGT1A3* **Inhibitor:***ABCB1*, *CYP2C9*, *CYP2C19*, *CYP2D6*, *CYP3A4*, *CYP3A5*, *HMGCR* **Inducer:***CYP2B6* **Transporter genes:***ABCA1*, *ABCB1*, *ABCB11*, *ABCC2*, *ABCG2*, *SLC22A8*, *SLCO1A2*, *SLCO1B1*, *SLCO1B3*, *SLCO2B1* **Pleiotropic genes:***IL1B*, *IL6*, *IL10*, *TP53*
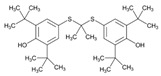	**Name: PROBUCOL** **IUPAC Name:** Phenol, 4,4′-[(1-methylethylidene)bis(thio)]bis[2,6-bis(1,1-dimethylethyl)-; acetone bis(3,5-di-tert-butyl-4-hydroxyphenyl) mercaptole **Molecular Formula:** C_31_H_48_O_2_S_2_ **Molecular Weight:** 516.84 **Mechanism:** Increases fecal loss of bile acid-bound low-density lipoprotein cholesterol, decreases synthesis of cholesterol and inhibits enteral cholesterol absorption. **Effect:** Adjunct to dietary therapy to decrease elevated serum total and LDL-C concentrations in primary hypercholesterolemia.	**Mechanistic genes:***APOB*, *APOC3*, *KCNH2*, *SCARB1* **Metabolic genes** **Substrate:***CYP2D6*, *CYP3A4*, *CYP3A5* **Pleiotropic genes:***TNF*
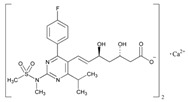	**Name: ROSUVASTATIN** **IUPAC Name:** 6-Heptenoic acid, 7-[4-(4-fluorophenyl)-6-(1-methylethyl)-2-[methyl(methylsulfonyl)amino]-5-pyrimidinyl]-3,5-dihydroxy-, calcium salt (2:1), (3R,5S,6E)-; [S-[R*,S*-(E)]]-7-[4-(4-fluorophenyl)-6-(1-methylethyl)-2-[methyl(methylsulfonyl)amino]-5-pyrimidinyl]-3,5-dihydroxy-6-heptenoic acid, calcium salt (2:1) **Molecular Formula:** [C_22_H_27_FN_3_O_6_S]_2_Ca **Molecular Weight:** 1001.14 **Mechanism:** Inhibitor of 3-hydroxy-3-methylglutaryl coenzyme A (HMG-CoA) reductase, rate-limiting enzyme in cholesterol synthesis. This results in compensatory increase in expression of LDL receptors on hepatocyte membranes and stimulation of LDL catabolism. **Effect:** Used with dietary therapy for hyperlipidemias to reduce elevations in total cholesterol (TC), LDL-C, apolipoprotein B, non-HDL-C, and triglycerides in primary hypercholesterolemia (elevations of 1 or more components present in Fredrickson type IIa, IIb, and IV hyperlipidemias). Treatment of primary dysbetalipoproteinemia (Fredrickson type III hyperlipidemia). Treatment of homozygous familial hypercholesterolemia. To slow progression of atherosclerosis as adjunct to TC- and LDL-C-lowering diet.	**Mechanistic genes:***ACE1*, *APOA1*, *APOA5*, *APOB*, *APOC3*, *APOE*, *CETP*, *FGB*, *ITGB3*, *LDLR*, *LIPC*, *LPL*, *NOS3*, *TCF20*, *USP5* **Metabolic genes** **Substrate:***CYP2C8*, *CYP2C9*, *CYP2C19*, *CYP2D6*, *CYP3A4*, *CYP3A5*, *CYP7A1*, *UGT1A3* **Inhibitor:***CETP*, *HMGCR*, *SLCO1B1* **Inducer:***CYP2B6*, *CYP2C9*, *CYP3A4*, *CYP3A5* **Transporter genes:***ABCA1*, *ABCB1*, *ABCB11*, *ABCC1*, *ABCC4*, *ABCG2*, *SLC10A1*, *SLCO1A2*, *SLCO1B1*, *SLCO1B3*, *SLCO2B1*, *SLC22A8*
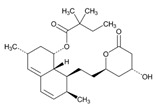	**Name: SIMVASTATIN** **IUPAC Name:** Butanoic acid, 2,2-dimethyl-, 1,2,3,7,8,8a-hexahydro-3,7-dimethyl-8-[2-(tetrahydro-4-hydroxy-6-oxo-2H-pyran-2-yl)ethyl]-1-naphthalenyl ester, [1S-[1α,3α,7β,8β(2S*,4S*),8aβ]]-; 2,2-dimethylbutyric acid, 8-ester with (4R,6R)-6-[2-[(1S,2S,6R,8S,8aR)-1,2,6,7,8,8a-hexahydro-8-hydroxy-2,6-dimethyl-1-naphthyl]ethyl]tetrahydro-4-hydroxy-2H-pyran-2-one **Molecular Formula:** C_25_H_38_O_5_ **Molecular Weight:** 418.57 **Mechanism:** Prodrug requiring hydrolysis in vivo for activity. Inhibits HMG-CoA reductase, causing subsequent reduction in hepatic cholesterol synthesis. Reduces serum concentrations of total cholesterol, LDL-C, Apo B, and triglycerides. Statins may slow progression and/or induce regression of atherosclerosis in coronary and/or carotid arteries, modulate blood pressure in hypercholesterolemic patients with hypertension, and possess anti-inflammatory activity. **Effect:** Secondary prevention of cardiovascular events in hypercholesterolemic patients with established coronary heart disease or at high risk for coronary heart disease. Hyperlipidemias (primary hypercholesterolemia, homozygous familial hypercholesterolemia, heterozygous familial hypercholesterolemia).	**Mechanistic genes:***APOA1*, *APOA5*, *APOB*, *APOC3*, *APOE*, *CETP*, *F2*, *FGB*, *GNB3*, *HMGCR*, *HTR3B*, *LDLR*, *LIPC*, *LPL*, *NOS3*, *PRNP*, *USP5*, *VCAM1* **Metabolic genes** **Substrate:***CYP2C8*, *CYP2C9*, *CYP2C19*, *CYP3A4*, *CYP3A5*, *CYP7A1*, *POR*, *UGT1A3* **Inhibitor:***CYP2C8*, *CYP2C9*, *CYP2C19*, *CYP2D6*, *CYP3A4*, *CYP3A5*, *HMGCR* **Inducer:***CYP2B6* **Transporter genes:***ABCA1*, *ABCB1*, *ABCB11*, *ABCC2*, *ABCC3*, *ABCG2*, *SLCO1B1*, *SLCO1B3* **Pleiotropic genes:***IL6*, *TNF*

**Table 3:** Pharmacogenomics of antithrombotic drugs.

Antithrombotic Drugs		
Vitamin K Antagonists		
Drug	Properties	Pharmacogenetics
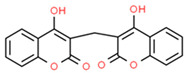	**Name: Dicoumarol** **IUPAC Name:** 4-hydroxy-3-[(4-hydroxy-2-oxo-2H-chromen-3-yl)methyl]-2H-chromen-2-one. **Molecular Formula:** C_19_H_12_O_6_ **Molecular Weight:** 336.295 Da. **Mechanism:** Inhibits vitamin K reductase, depletes vitamin KH2, cofactor for vitamin K-dependent protein-carboxylation, limits gamma-carboxylation, and activates vitamin K-dependent coagulant proteins. Inhibits synthesis of vitamin K-dependent coagulation factors II, VII, IX, and X and anticoagulant proteins C and S. Depresses vitamin K-dependent coagulation factors II, VII, and X, lowers prothrombin levels and the amount of fibrin-bound thrombin, reducing thrombogenicity. **Effect:** Antithrombotic agents. Vitamin K antagonists.	**Mechanistic genes:***CRYZ*, *F2*, *F7*, *F9*, *F10*, *NQO1*, *PROC*, *PROS1*, *VKORC1* **Metabolic genes** **Substrate:***CYP2C9* **Inhibitor:***CYP2C6*, *CYP2C11* **Transporter genes:***ALB*
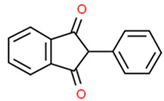	**Name: Phenindione** **IUPAC Name:** 2-phenyl-2,3-dihydro-1H-indene-1,3-dione. **Molecular Formula:** C_15_H_10_O_2_ **Molecular Weight:** 222.24 Da. **Mechanism:** Similar mode of action as Dicoumarol. **Effect:** Antithrombotic agents. Vitamin K antagonists.	**Mechanistic genes:***ANXA5*, *F2*, *F7*, *F9*, *F10*, *PROC*, *PROS1*, *VKORC1*
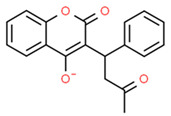	**Name:Warfarin** **IUPAC Name**, (1) 2H-1-benzopyran-2-one, 4-hydroxy-3-(3-oxo-1-phenylbutyl)-, sodium salt, (2) 3-(α-acetonylbenzyl)-4-hydroxycoumarin sodium salt **Molecular Formula:** C_19_H_15_NaO_4_ **Molecular Weight:** 330.31 Da **Mechanism:** Competitively inhibits subunit-1 of multi-unit VKOR complex, depleting vitamin K reserves. Antithrombogenic effects occur after functional coagulation factors IX and X are diminished. Phytonadione (vitamin K1) reverses anticoagulant effect. Slightly affects platelet-rich arterial thrombi-adherence to abnormal vessel wall. **Effect:** Antithrombotic agents, Anticoagulants, Coumarin Derivatives. Vitamin K Antagonist	**Mechanistic genes:** F2, F5, F7, F9, F10, NR1I2, PROC, VKORC1 **Metabolic genes** **Substrate:** CALU, CYP1A2, CYP2C8, CYP2C9, CYP2C18, CYP2C19, CYP3A4, CYP3A5, EPHX1, GGCX **Inhibitor:** *CYP2C9*, *CYP2C19*, *VKORC1* **Inducer:** *CYP2C9*, *CYP3A4* **Transporter genes:** ABCB1, ALB, ORM1 **Pleiotropic genes:** APOE
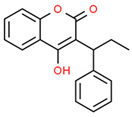	**Name: Phenprocoumon** **IUPAC Name:** 4-hydroxy-3-(1-phenylpropyl)-2H-chromen-2-one. **Molecular Formula:** C_18_H_16_O_3_ **Molecular Weight:** 280.32 Da. **Mechanism:** as per Dicoumarol **Effect:** Antithrombotic agents. Vitamin K antagonists.	**Mechanistic genes:***F2*, *F7*, *F9*, *F10*, *PROC*, *PROS1*, *VKORC1* **Metabolic genes** **Substrate:***CYP2C8*, *CYP2C9*, *CYP3A4* **Transporter genes:***ALB*, *ORM1*
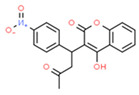	**Name:Acenocoumarol** **IUPAC Name**, (1) 2H-1-benzopyran-2-one, 4-hydroxy-3-[1-(4-nitrophenyl)-3-oxobutyl]-, (2) 3-(α-acetonyl-p-nitrobenzyl)-4-hydroxycoumarin **Molecular Formula:** C_19_H_15_NO_6_ **Molecular Weight:** 353.33 Da **Mechanism:** Interferes with hepatic synthesis of vitamin K-dependent coagulation factors II, VII, IX, X. **Effect:** Antithrombotic agents. Vitamin K antagonists.	**Mechanistic genes:***CALU*, *F2*, *F7*, *F9*, *F10*, *VKORC1* **Metabolic genes** **Substrate:***CYP1A2*, *CYP2C9*, *CYP2C18*, *CYP2C19*, *CYP3A4* **Transporter genes:***ABCB1*, *ALB*, *ORM* Pleiotropic gens: *APOE*
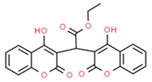	**Name: Ethyl biscoumacetate** **IUPAC Name:** ethyl 2,2-bis(4-hydroxy-2-oxochromen-3-yl)acetate. **Molecular Formula:** C_22_H_16_O_8_ **Molecular Weight:** 408.4 Da. **Mechanism:** Anticoagulant, mode of action similar to that of warfarin. **Effect:** Antithrombotic agents. Vitamin K antagonists.	**Mechanistic genes:***F2*, *F7*, *F9*, *F10*, *VKORC1* **Metabolic genes** **Substrate:***CYP3A4* **Inhibitor:***GLUL*
**Heparins**		
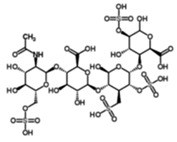	**Name: Heparin** **IUPAC Name:** (2S,3S,4R,5R,6R)-3-({(2R,3R,4R,5S,6R)-3-(acetylamino)-4,5-dihydroxy-6-[(sulfooxy)methyl]tetrahydro-2H-pyran-2-yl}oxy)-6-{[(2S,3S,4S,5R,6S)-6-{[(2R,3S,4S,5R)-2-carboxy-4,6-dihydroxy-5-(sulfooxy)tetrahydro-2H-pyran-3-yl]oxy}-2-hydroxy-4-(sulfomethyl)-5-(sulfooxy)tetrahydro-2H-pyran-3-yl]oxy}-4,5-dihydroxytetrahydro-2H-pyran-2-carboxylic acid. **Molecular Formula:** C_26_H_41_NO_34_S_4_. **Molecular Weight:** 1039.85 Da **Mechanism:** Potentiates antithrombin III activity; inactivates thrombin, coagulation factors IX, X, XI, XII, and plasmin; prevents conversion of fibrinogen to fibrin. **Effect:** Antithrombotic agents. Heparin group.	**Mechanistic genes:***F9*, *F10*, *F11*, *F12*, *FCGR2A*, *FCGR3A*, *FGF1*, *FGF19*, *FGF2*, *FGF4*, *FGFR1*, *FGFR2*, *FGFR4*, *HGF*, *ITGB3*, *LIPA*, *PF4*, *PROC*, *SELP*, *SERPINA5*, *SERPINC1*, *VWF* **Metabolic genes** **Substrate:***HPSE* **Transporter genes:***ABCC1*, *SERPINA7* **Pleiotropic genes:***APP*
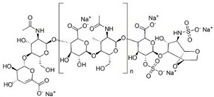	**Name: Enoxaparin** **Mechanism:** Enhances the inhibition rate of clotting proteases by antithrombin III, impairing normal hemostasis; strongly inhibits factor Xa. **Effect:** Antithrombotic agents. Heparin group.	**Mechanistic genes:***ACE*, *F2*, *F5*, *F10*, *FCGR3A*, *IL1RN*, *ITGB3*, *MPO*, *SERPINC1*, *THBD* **Metabolic genes** **Substrate:***HPSE* **Transporter genes:***SERPINA7*
**Platelet aggregation inhibitors**		
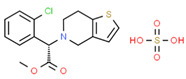	**Name:Clopidogrel** **IUPAC Name:** (1) Thieno[3,2-c]pyridine-5(4H)-acetic acid, α-(2-chlorophenyl)-6,7-dihydro-, methyl ester, (S)-, sulfate (1:1), (2) Methyl (+)-(S)-α-(o-chlorophenyl)-6,7-dihydrothieno[3,2-c]pyridine-5(4H)-acetate, sulfate (1:1) **Molecular Formula:** C_16_H_16_ClNO_2_S **Molecular Weight:** 419.90 Da **Mechanism:** Platelet inhibitor; irreversibly binds to P2Y_12_ ADP receptors on platelets preventing ADP binding to same receptors, activating the glycoprotein GPII_b_/III_a_ complex, and reducing platelet aggregation. Inhibits ADP-mediated release of platelet dense granule (e.g., ADP, Ca^2+^, and serotonin) and α-granule (e.g., fibrinogen and thrombospondin) contents that augment platelet aggregation. **Effect:** Antithrombotic agents. Platelet aggregation inhibitors excl. heparin.	**Mechanistic genes:***ITGA2B*, *ITGB3*, *P2RY12* **Metabolic genes** **Substrate:***CES1*, *CYP1A2*, *CYP2B6*, *CYP2C9*, *CYP2C19*, *CYP3A4*, *CYP3A5* **Inhibitor:***CYP2B6*, *CYP2C8*, *CYP2C9*, *CYP2C19* **Transporter genes:***ABCB1*, *SLC22A1*, *SLC22A2*
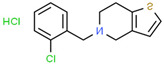	**Name:Ticlopidine** **IUPAC Name**, (1) Thieno[3,2-c]pyridine, 5-[(2-chlorophenyl)methyl]-4,5,6,7-tetrahydro-, hydrochloride, (2) 5-(o-chlorobenzyl)-4,5,6,7-tetrahydrothieno-[3,2-c]pyridine hydrochloride **Molecular Formula:** C_14_H_14_ClNS **Molecular Weight:** 300.25 Da **Mechanism:** Irreversibly blocks P2Y_12_ receptors as an active metabolite, preventing GPII_b_/III_a_ receptor complex activation, reducing platelet aggregation. **Effect:** Antithrombotic agents. Platelet aggregation inhibitors excl. heparin.	**Mechanistic genes:***ITGB3*, *P2RY12* **Metabolic genes** **Substrate:***CYP2B6*, *CYP2C19*, *CYP2D6*, *CYP3A4*, *MPO* **Inhibitor:***CYP1A2*, *CYP2B6*, *CYP2C8*, *CYP2C9*, *CYP2C19*, *CYP2D6*, *CYP2E1*, *CYP3A4* **Pleiotropic genes:***HLA-B*
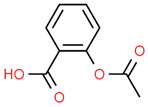	**Name:Acetylsalicylic acid** **IUPAC Name:** Benzoic acid, 2-(acetyloxy)-, (2) salicylic acid acetate **Molecular Formula:** C_9_H_8_O_4_ **Molecular Weight:** 180.16 Da **Mechanism:** Inhibits prostaglandin synthesis; acts on the preoptic area of the anterior hypothalamus to reduce fever. Blocks prostaglandin synthetase activity, preventing thromboxane A_2_ formation. **Effect:** Antithrombotic agents. Platelet aggregation inhibitors excl. heparin.	**Mechanistic genes:***AKR1C1*, *CASP1*, *CASP3*, *CCNA2*, *CCND1*, *EDNRA*, *GP1BA*, *GP6*, *HSPA5*, *IKBKB*, *MAP2K4*; *MYC*, *NFKBIA*, *PCNA*, *PRKAs*, PTGER1, PTGER2, PTGER3, PTGER4, PTGES, PTGIR, PTGS1, PTGS2, *RPS6KA3*, TBX21, TBXA2R, TNFAIP6, TP53 **Metabolic genes** **Substrate:***ACSM1*, *CYP2C9*, *CYP3A4*, *GLYAT*, *NAT2*, *UGT1A1*, *UGT1A10*, *UGT1A3*, *UGT1A6*, *UGT1A7*, *UGT1A9*, *UGT2B4*, *UGT2B7* **Inhibitor:***CYP19A1*, *PTGS1*, *PTGS2* **Inducer:***CYP2C19*, *CYP2E1* Transporter genes, *ABCB1*, *SLC22A6*, *SLC22A8*
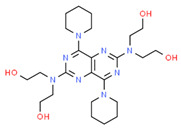	**Name:Dipyridamole** **IUPAC Name:** Ethanol, 2,2′,2′’,2′’’-[(4,8-di-1-piperidinylpyrimido[5,4-d]pyrimidine-2,6-diyl)dinitrilo]tetrakis-, (2) 2,2′,2′’,2′’-[(4,8-sipiperidinopyrimido[5,4-d]pyrimidine-2,6-diyl)dinitrilo]tetraethanol **Molecular Formula:** C_24_H_40_N_8_O_4_ **Molecular Weight:** 504.63 Da **Mechanism:** Non-nitrate coronary vasodilator; inhibits adenosine deaminase and phosphodiesterase activity, inducing accumulation of adenosine, adenine nucleotides, and cAMP which inhibit platelet aggregation, causing vasodilation. May stimulate prostacyclin or PGD_2_ activity. **Effect:** Antithrombotic agents. Platelet aggregation inhibitors excl. heparin.	**Mechanistic genes:***ADA*, *ORM1*, *PDE10A*, *PDE4A*, *PDE5A*, *PTGDR2*, *RCAN1* **Transporter genes:***ABCB1*, *ABCB11*, *ABCC4*, *ABCC5*, *SLCO1B1*, *SLCO1B3*, *SLCO2B1*
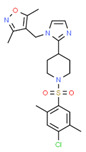	**Name:Abciximab** **IUPAC Name:** (1) Immunoglobulin G1, anti-(human integrin αIIbβ3) Fab fragment (human-mouse monoclonal c7E3 clone p7E3VHhCγ1 γ1-chain), disulfide with human-mouse monoclonal c7E3 clone p7E3VκhCκ κ-chain. **Molecular Formula:** C_22_H_27_ClN_4_O_3_S **Molecular Weight:** 462.993 Da **Mechanism:** Monoclonal antiglycoprotein II_b_/III_a_ receptor antibody; GPII_b_/III_a_ is the major platelet surface receptor in platelet aggregation. Blocks vitronectin receptor-mediated cell adhesion, and Mac-1 receptor on monocytes and neutrophils, inhibiting adhesion to monocyte cells. **Effect:** Antithrombotic agents. Platelet aggregation inhibitors excl. heparin. Glycoprotein IIb/IIIa Inhibitor.	**Mechanistic genes:***FCGR2A*, *FCGR2B*, *ITGA2B*, *ITGB3*, *P2RY1*, *VTN* **Metabolic genes** **Substrate:***CYP1A2*, *CYP2C19*
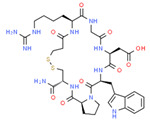	**Name:Eptifibatide** **IUPAC Name:** (1) N6-amidino-N2-(3-mercaptopropionyl)-L-lysylglycyl-L-α-aspartyl-L-tryptophyl-L-prolyl-L-cysteinamide, cyclic (1–6)-disulfide. **Molecular Formula:** C_35_H_49_N_11_O_9_S_2_ **Molecular Weight:** 831.96 Da **Mechanism:** Blocks the platelet GPII_b_/III_a_ receptor, reversibly inhibiting platelet aggregation, preventing thrombosis. **Effect:** Antithrombotic agents, platelet aggregation inhibitors, and glycoprotein IIb/IIIa Inhibitors.	**Mechanistic genes:***IL6*, *ITGA2B*, *ITGB3*, *P2RY1*, *TBXAS1* **Metabolic genes** **Substrate:***CYP1A2*, *CYP2C19*
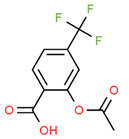	**Name: Triflusal** **IUPAC Name:** 2-(acetyloxy)-4-(trifluoromethyl)benzoic acid. **Molecular Formula:** C_10_H_7_F_3_O_4_ **Molecular Weight:** 248.15 Da **Mechanism:** irreversible COX-1 inhibitor in platelets; spares the arachidonic acid pathway in endothelial cells; favors nitric oxide production. **Effect:** Antithrombotic agents. Platelet aggregation inhibitors excl. heparin.	**Mechanistic genes:***NFKB1*, *NOS2*, *PDE10A*, *PTGS1* **Metabolic genes** **Substrate:***CYP2C8* **Transporter genes:***ALB*
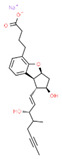	**Name: Beraprost** **IUPAC Name:** sodium 4-[(2S,3R,4R,6S)-4-hydroxy-3-[(1E,3S)-3-hydroxy-4-methyloct-1-en-6-yn-1-yl]-7-oxatricyclo[6.4.0.0^{2,6}]dodeca-1(8),9,11-trien-9-yl]butanoate. **Molecular Formula:** C_24_H_29_NaO_5_ **Molecular Weight:** 420.47 Da **Mechanism:** Binds prostacyclin membrane receptors, inhibiting Ca^2+^ release from intracellular stores, promoting vasodilation. **Effect:** Antithrombotic agents. Platelet aggregation inhibitors excl. heparin.	**Mechanistic genes:** *PTGIR* **Metabolic genes** **Substrate:** *CYP2C8*
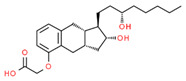	**Name: Treprostinil** **IUPAC Name:** 2-{[(1R,2R,3aS,9aS)-2-hydroxy-1-[(3S)-3-hydroxyoctyl]-1H,2H,3H,3aH,4H,9H,9aH-cyclopenta[b]naphthalen-5-yl]oxy}acetic acid. **Molecular Formula:** C_23_H_34_O_5_ **Molecular Weight:** 390.51 Da **Mechanism:** Prostacyclin vasodilator; binds to the prostacyclin receptor inducing vasodilation of pulmonary and systemic arterial vascular beds, inhibits platelet aggregation and inflammatory cytokine production. **Effect:** Antithrombotic agents. Platelet aggregation inhibitors excl. heparin.	**Mechanistic genes:***P2RY12*, *PPARD*, *PTGIR* **Metabolic genes** **Substrate:***CYP2C9*
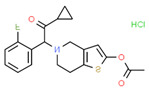	**Name,Prasugrel** **IUPAC Name:** (1) 5-[(1RS)-2-cyclopropyl-1-(2-fluorophenyl)-2-oxoethyl]-4,5,6,7-tetrahydrothieno[3,2-c]pyridin-2-yl acetate hydrochloride. (2) 2-[2-(Acetyloxy)-6,7-dihydrothieno[3,2-c]pyridin-5(4H)-yl]-1-cyclopropyl-2-(2-fluorophenyl)ethanone hydrochloride **Molecular Formula:** C_20_H_21_ClFNO_3_S **Molecular Weight:** 409.90 Da **Mechanism:** P2Y_12_ platelet inhibitor; impairs ADP-mediated activation of the GPII_b_/III_a_ complex. **Effect:** Antithrombotic agents. Platelet aggregation inhibitors excl. heparin.	**Mechanistic genes:***P2RY12* **Metabolic genes** **Substrate:***CES1*, *CES2*, *CYP2B6*, *CYP2C9*, *CYP2C19*, *CYP2D6*, *CYP3A4*, *GSTs*, *POR* **Inhibitor:***CYP2B6*, *CYP2C9*, *CYP2C19*, *CYP2D6*, *CYP3A4*, *CYP3A5* **Transporter genes:***ABCB1*, *ALB*
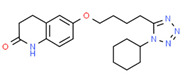	**Name:Cilostazol** **IUPAC Name:** (1) 2(1H)-quinolinone, 6-[4-(1-cyclohexyl-1H-tetrazol-5-yl)butoxy]-3,4-dihydro-, (2) 6-[4-(1-cyclohexyl-1H-tetrazol-5-yl)butoxy]-3,4-dihydrocarbostyril **Molecular Formula:** C_20_H_27_N_5_O_2_ **Molecular Weight:** 369.46 Da **Mechanism:** Antiplatelet agent and vasodilator; inhibits PDE_3_ activation, increasing cAMP concentrations in platelets and blood vessels and mediating arterial vasodilation and inhibition of platelet aggregation. Reduces plasma triglyceride, but increases high-density lipoprotein cholesterol, levels. **Effect:** Antithrombotic agents. Platelet aggregation inhibitors excl. heparin. Phosphodiesterase Enzyme Inhibitor.	**Mechanistic genes:***PDE3A* **Metabolic genes** **Substrate:***CYP1A2*, *CYP1B1*, *CYP2C8*, *CYP2C19*, *CYP2D6*, *CYP3A4*, *CYP3A5*, *CYP3A7* **Transporter genes:***ABCB1*
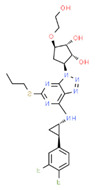	**Name:Ticagrelor** **IUPAC Name:** (1S,2S,3R,5S)-3-[7-[[(1R,2S)-2-(3,4-difluorophenyl)cyclopropyl]amino]-5-propylsulfanyltriazolo[4,5-d]pyrimidin-3-yl]-5-(2-hydroxyethoxy)cyclopentane-1,2-diol **Molecular Formula:** C_23_H_28_F_2_N_6_O_4_S **Molecular Weight:** 522.57 Da **Mechanism:** P2Y_12_ platelet inhibitor; couples with Gα_i2_ and other G_i_ proteins to inhibit adenylyl cyclase. Activates PI3K, Akt, Rap1b, and K^+^ channels, mediating hemostasis and platelet aggregation. P2Y_12_ receptor blockade reduces development of occlusive thromboses, risk of MI and ischemic stroke. **Effect:** Antithrombotic agents. Platelet aggregation inhibitors excl. heparin. Selective adenosine diphosphate (ADP) receptor antagonist.	**Mechanistic genes:***P2RY12* **Metabolic genes** **Substrate:***CYP2C19*, *CYP3A4*, *CYP3A5*, *UGTs* **Inhibitor:***ABCB1*, *CYP1A2*, *CYP2C9*, *CYP3A4* **Inducer:***CYP2B6*, *CYP2C9* **Transporter genes:***ABCB1*, *ALB*
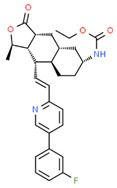	**Name: Vorapaxar** **IUPAC Name:** ethyl N-[(1R,3aR,4aR,6R,8aR,9S,9aS)-9-[(1E)-2-[5-(3-fluorophenyl)pyridin-2-yl]ethenyl]-1-methyl-3-oxo-dodecahydronaphtho[2,3-c]furan-6-yl]carbamate. **Molecular Formula:** C_29_H_33_FN_2_O_4_ **Molecular Weight:** 492.58 Da **Mechanism:** Reversible PAR-1 antagonist, inhibits thrombin- and TRAP-induced platelet aggregation. Reduces thrombotic cardiovascular events in patients with a history of MI or PAD. **Effect:** Antithrombotic agents. Platelet aggregation inhibitors excl. heparin.	**Mechanistic genes:***F2R* **Metabolic genes** **Substrate:***CYP2J2*, *CYP3A4* **Transporter genes:***ABCB1*
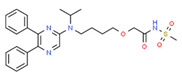	**Name: Selexipag** **IUPAC Name:** 2-{4-[(5,6-diphenylpyrazin-2-yl)(propan-2-yl)amino]butoxy}-N-methanesulfonylacetamide. **Molecular Formula:** C_26_H_32_N_4_O_4_S **Molecular Weight:** 496.63 Da **Mechanism:** Selective PGI_2_ receptor agonist. Potent vasodilator with antiproliferative, anti-inflammatory, and antithrombotic effects. **Effect:** Antithrombotic agents. Platelet aggregation inhibitors excl. heparin.	**Mechanistic genes:***PTGIR* **Metabolic genes** **Substrate:***CES1*, *CYP2C8*, *CYP3A4* **Transporter genes:***ABCB1*, *SLCO1B1*, *SLCO1B3*
**Direct thrombin inhibitors**		
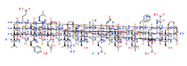	**Name: Desirudin** **Molecular Formula:** C_287_H_440_N_80_O_110_S_6_ **Molecular Weight:** 6963.52 Da **Mechanism:** Direct, highly selective thrombin inhibitor. Reversibly binds to the active thrombin site of free and clot-associated thrombin. Inhibits fibrin formation, activation of coagulation factors V, VII, and XIII, and thrombin-induced platelet aggregation. **Effect:** Antithrombotic agents. Direct thrombin inhibitors.	**Mechanistic genes:***F2*, *F5*, *F7*, *F13A1* **Metabolic genes** **Substrate:***CPA1*
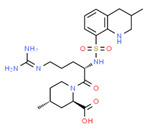	**Name:Argatroban** **IUPAC Name:** (1) (2R,4R)-1-{N5-(Diaminomethylene)-N2-[(3-methyl-1,2,3,4-tetrahydro-8-quinolinyl)sulfonyl]-L-ornithyl}-4-methyl-2-piperidinecarboxylic acid **Molecular Formula:** C_23_H_36_N_6_O_5_S **Molecular Weight:** 508.634 Da **Mechanism:** Direct thrombin inhibitor; Reversibly binds to the active thrombin site of free and clot-associated thrombin. Inhibits fibrin formation, activation of coagulation factors V, VIII, and XIII, protein C, and platelet aggregation. **Effect:** Antithrombotic agents. Direct thrombin inhibitors.	**Mechanistic genes:***F2*, *F5*, *F8*, *F13*, *PROC* **Metabolic genes** **Substrate:***CYP3A4*, *CYP3A5*
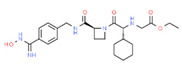	**Name: Ximelagatran** **IUPAC Name:** ethyl 2-{[(1R)-1-cyclohexyl-2-[(2S)-2-[({4-[(Z)-N’-hydroxycarbamimidoyl]phenyl}methyl)carbamoyl]azetidin-1-yl]-2-oxoethyl]amino}acetate. **Molecular Formula:** C_24_H_35_N_5_O_5_ **Molecular Weight:** 473.56 Da **Mechanism:** Bioconverted to the active moiety, melagatran, which inhibits clot-bound thrombin. **Effect:** Antithrombotic agents. Direct thrombin inhibitors.	**Mechanistic genes:** *F2* **Metabolic genes** **Substrate:** *CYP2C9*
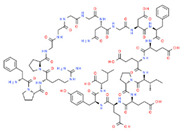	**Name:Bivalirudin** **IUPAC Name:** 1) L-Leucine, D-phenylalanyl-L-prolyl-L-arginyl-L-prolylglycylglycylglycylglycyl-L-asparaginylglycyl-L-α-aspartyl-L-phenylalanyl-L-α-glutamyl-L-α-glutamyl-L-isoleucyl-L-prolyl-L-α-glutamyl-L-α-glutamyl-L-tyrosyl-, (2) D-phenylalanyl-L-prolyl-L-arginyl-L-prolylglycylglycylglycylglycyl-L-asparaginylglycyl-L-α-aspartyl-L-phenylalanyl-L-α-glutamyl-L-α-glutamyl-L-isoleucyl-L-prolyl-L-α-glutamyl-L-α-glutamyl-L-tyrosyl-L-leucine **Molecular Formula:** C_98_H_138_N_24_O_33_ **Molecular Weight:** 2180.29 Da **Mechanism:** Reversible direct thrombin inhibitor for heparin-induced thrombocytopenia. Inhibits thrombin by binding to its catalytic and anion-binding exosite, preventing thrombin-mediated cleavage of fibrinogen to fibrin, activation of factors V, VIII, and XIII, conversion of fibrinogen to fibrin, and platelet activation and aggregation. **Effect:** Antithrombotic agents. Direct thrombin inhibitors.	**Mechanistic genes:***F2*, *F5*, *F8*, *F13*, *FGA* **Metabolic genes** **Inhibitor:***MPO*
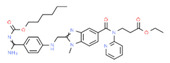	**Name:Dabigatran** **IUPAC Name:** Ethyl 3-[[[2-[[[4-[[[(hexyloxy)carbonyl]amino]iminomethyl]phenyl]amino]methyl]-1-methyl-1H-benzimidazol-5-yl]carbonyl](pyridin-2-yl)amino]propanoate **Molecular Formula:** C_34_H_41_N_7_O_5_ **Molecular Weight:** 627.73 Da **Mechanism:** Inhibits coagulation by preventing thrombin-mediated effects, including cleavage of fibrinogen to fibrin monomers, activation of factors V, VIII, XI and XIII, and inhibition of thrombin-induced platelet aggregation. **Effect:** Antithrombotic agents. Direct thrombin inhibitors.	**Mechanistic genes:***F2*, *F5*, *F8*, *F11*, *F13*, *FGA* **Metabolic genes** **Substrate:***CES1* **Transporter genes:***ABCB1*
**Direct factor Xa inhibitors**		
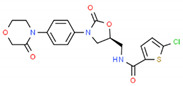	**Name: Rivaroxaban** **IUPAC Name:** 5-chloro-N-{[(5S)-2-oxo-3-[4-(3-oxomorpholin-4-yl)phenyl]-1,3-oxazolidin-5-yl]methyl}thiophene-2-carboxamide. **Molecular Formula:** C_19_H_18_ClN_3_O_5_S **Molecular Weight:** 435.88 Da **Mechanism:** Anticoagulant, irreversibly inhibits free and clot bound factor Xa; treating DVT and PE. **Effect:** Antithrombotic agents. Direct Factor Xa inhibitors.	**Mechanistic genes:***F2*, *F10* **Metabolic genes** **Substrate:***CYP2J2*, *CYP3A4*, *CYP3A5* **Transporter genes:***ABCB1*, *ABCG2*
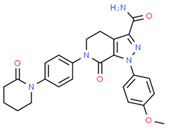	**Name: Apixaban** **IUPAC Name:** 1-(4-methoxyphenyl)-7-oxo-6-[4-(2-oxopiperidin-1-yl)phenyl]-1H,4H,5H,6H,7H-pyrazolo[3,4-c]pyridine-3-carboxamide. **Molecular Formula:** C_25_H_25_N_5_O_4_ **Molecular Weight:** 459.50 Da **Mechanism:** Inhibits factor Xa, independent of antithrombin III. Inhibits prothrombin, preventing thrombus formation. **Effect:** Antithrombotic agents. Direct Factor Xa inhibitors.	**Mechanistic genes:***F2*, *F5*, *F10* **Metabolic genes** **Substrate:***CYP2C8*, *CYP2C19*, *CYP2C9*, *CYP1A2*, *CYP2J2*, *CYP3A4*, *CYP3A5* **Inhibitor:***CYP2C19* **Transporter genes:***ABCB1*, *ABCG2*
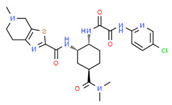	**Name: Edoxaban** **IUPAC Name:** N’-(5-chloropyridin-2-yl)-N-[(1S,2R,4S)-4-(dimethylcarbamoyl)-2-{5-methyl-4H,5H,6H,7H-[1,3]thiazolo[5,4-c]pyridine-2-amido}cyclohexyl]ethanediamide. **Molecular Formula:** C_24_H_30_ClN_7_O_4_S **Molecular Weight:** 548.06 Da **Mechanism:** Selective Factor Xa inhibitor. **Effect:** Antithrombotic agents. Direct Factor Xa inhibitors.	**Mechanistic genes:** *F10* **Transporter genes:** *ABCB1*
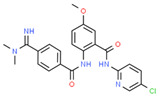	**Name: Betrixaban** **IUPAC Name:** N-(5-chloropyridin-2-yl)-2-[4-(N,N-dimethylcarbamimidoyl)benzamido]-5-methoxybenzamide. **Molecular Formula:** C_23_H_22_ClN_5_O_3_ **Molecular Weight:** 451.91 Da **Mechanism:** Cofactor-independent direct inhibitor of free and prothrombinase-bound Factor Xa. **Effect:** Antithrombotic agents. Direct Factor Xa inhibitors.	**Mechanistic genes:***F2*, *F10* **Transporter genes:***ABCB1*, *KCNH2*

**Table 4:** COVID-19 pharmacogenetics.

	*ABCB1*			*ABCC2*			*CYP2C9*			*CYP2C19*			*CYP2D6*			*CYP3A4*			*CYP3A5*			*NAT2*			*SLCO1B1*		
**ANTIVIRALS**																											
CHLOROQUINE														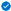			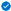			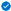							
HYDROXYCHLOROQUINE														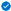			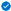			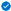							
LOPINAVIR		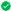			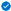			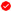			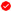			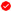			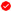			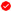						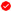	
REMSEDIVIR		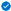						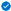						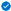			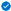			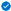						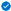	
RITONAVIR		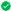			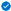			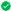			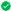			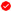			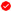			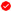						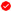	
**ANTIPYRETICS**																											
PARACETAMOL	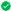	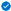			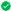			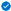					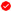	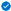		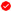	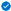			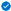			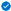				
**CORTICOSTEROIDS FOR SYSTEMIC USE**																											
ALDOSTERONE	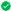	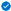															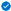			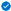							
BECLOMETASONE		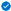															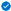			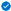							
BETAMETASONE		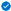															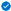			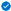							
BUDESONIDE		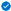														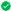	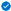	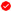	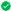	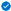	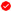						
CORTISONE		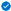															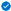			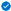							
CICLESONIDE		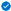												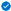			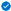			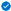							
DEFLAZACORT																	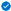			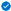							
DEXAMETASONE	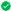	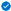	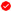		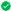			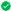			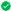			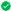		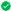	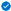	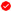	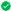	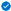	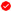						
FLUNISOLIDE		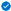												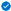			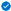			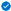							
FLUTICASONE		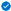												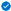			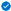			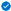							
HYDROCORTISONE		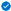															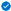			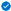			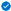				
METHYLPRENISOLONE		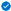															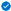	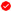		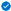	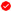						
MOMETASONE		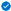															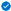			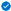							
PREDNISOLONE	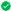	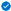	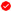													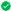	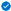	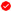	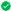	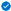	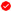						
RIMEXOLONE		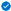																									
TIXOCORTOL		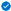																									
TRIAMCINOLONE		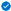																									
**NON-STEROIDAL ANTIINFLAMMATORY PRODUCTS**																											
ACECLOFENAC								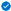																			
BENZYDAMINE											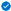			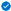			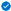			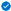							
BENOXAPROFEN																											
CELECOXIB		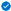						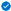						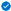	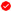		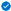			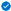							
DEXIBUPROFEN											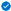																
DICLOFENAC	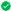	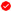			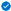			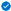	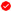		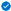			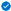		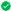	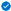	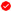	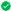	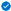	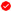					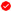	
ETODOLAC								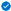	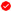								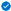			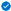							
ETORICOXIB								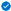			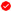			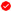		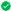	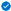	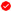	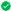	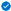	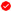						
FENOPROFEN								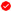																			
FLURBIPROFEN								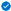	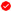					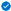			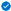			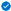							
IBUPROFEN		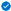	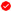					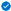	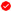		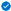			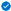			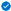			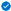							
INDOMETHACIN	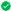	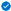	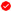		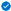	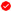		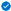	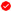		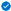	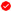		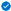			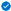			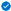						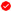	
INDOPROFEN																											
KETOPROFEN								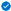	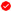																		
KETOROLAC																											
LORNOXICAM								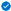						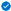			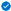			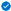							
LUMIRACOXIB								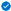			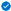						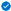			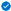							
MEFENAMIC ACID								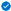	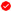																		
MELOXICAM		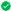						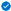	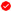								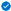			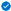							
NABUMETONE								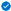			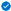			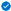			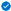			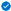							
NAPROXEN		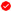						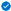																			
NIFLUMIC ACID																											
NIMESULIDE		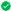																									
OXAPROZIN								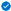																			
PENICILLAMINE																										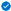	
PIROXICAM								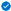	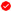								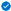			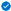							
ROFECOXIB								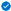																			
SULINDAC		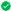						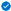									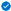			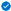							
SUPROFEN								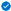																			
TENOXICAM								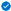	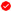								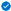			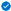							
VALDECOXIB								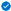									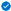			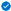							
**ANTIHYPERTENSIVE AGENTS**																											
AMLODIPINE		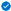	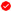					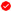						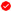			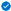	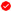		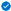	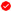						
ATENOLOL														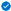													
BISOPROLOL		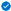												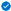			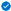			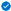							
CANDESARTAN		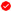						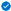	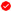																		
CARVEDILOL		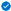	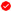					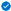			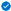			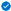			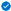			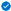							
ENALAPRIL																	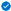			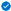							
HYDROCHLOROTIAZIDE																											
INDAPAMIDE																	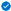			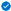							
IRBESARTAN		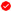						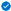	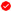					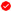			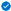	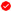		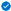	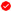						
ISOSORBIDE																	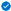			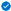							
LERCANIDIPINE																											
LISINOPRIL		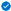															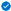			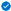							
LOSARTAN		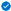						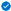	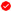		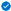	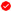					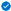	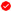		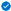	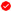						
METOPROLOL	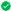	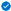									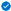			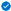	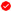												
NEBIVOLOL											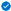			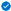													
NIFEDIPINE	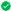	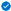	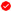					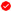						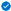	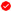		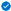	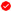		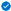	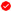						
OLMERSARTAN		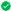		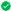	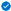			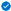																		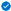	
PERINDOPRIL																											
RAMIPRIL																											
TELMISARTAN		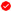			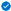	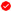		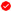			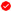						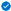			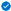							
VALSARTAN								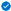	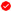																	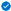	
**DIURETICS**																											
AMILORIDE		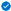	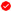																								
CONIVAPTAN																	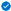	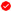		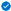	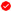						
FUROSEMIDE					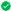																						
INDAPAMIDE																	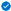			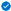							
MONTELUKAST		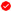						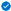	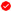								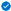			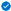							
TERBUTALINE																											
TOLVAPTAN		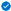	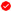														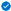			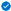							
TORASEMIDE								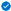																		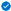	
TRIAMTERENE																	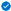			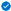							

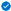
 Substrate; 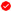 Inhibitor; 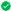 Inducer.
